# Supplementary material for: Agricultural non-point source pollution and health of the elderly in rural China
Source: PLoS One. 2022 Oct 14;17(10):e0274027. doi: 10.1371/journal.pone.0274027 (PMC9565375; doi:10.1371/journal.pone.0274027)
Supplement: S1 Fig — (DOCX) [file pone.0274027.s001.docx]

**S1 Fig. Fertilizer input and fertilizer loss in China.**
