# Supplementary figures and images for: Agricultural non-point source pollution and health of the elderly in rural China
Source: PLoS One. 2022 Oct 14;17(10):e0274027. doi: 10.1371/journal.pone.0274027 (PMC9565375; doi:10.1371/journal.pone.0274027)

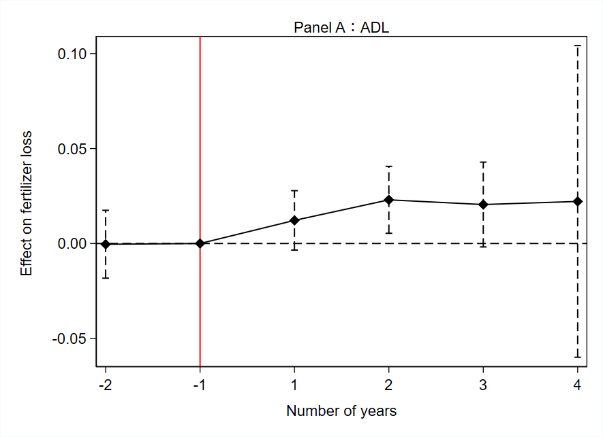

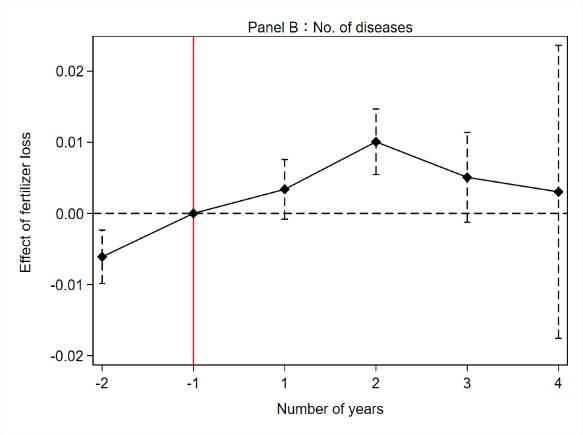

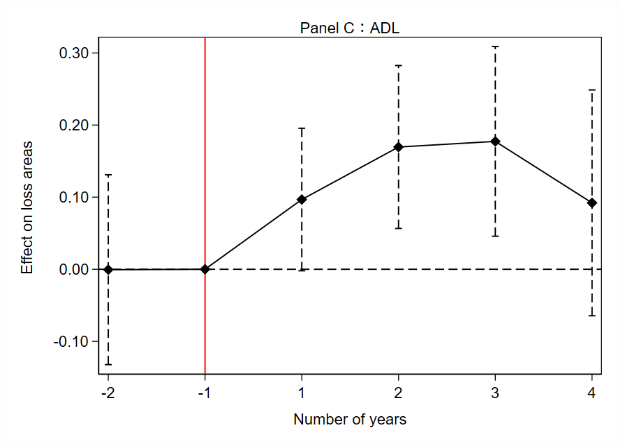

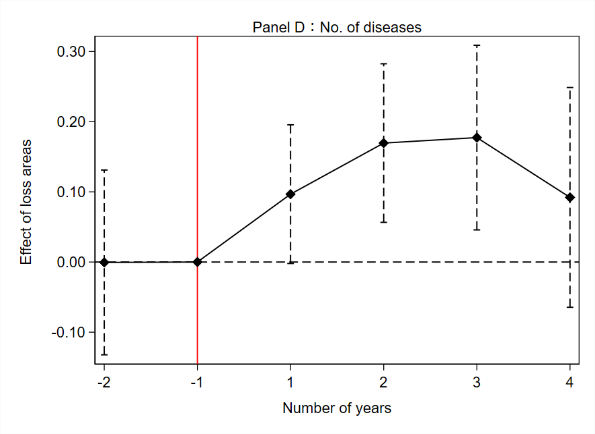


**S2 Fig. Parallel trend test.**

Supplement: S2 Fig — (DOCX) [file pone.0274027.s002.docx]

**
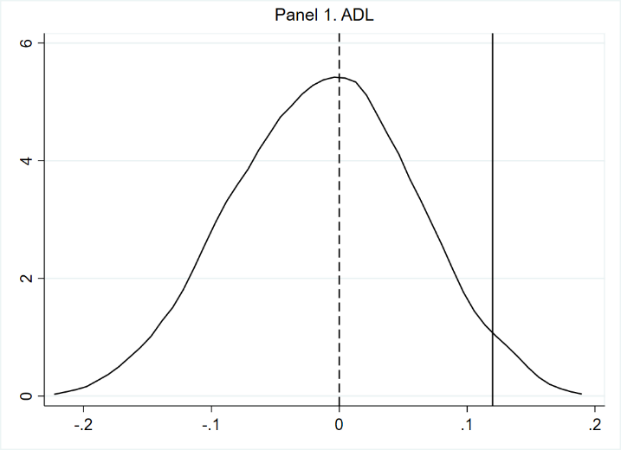
** **
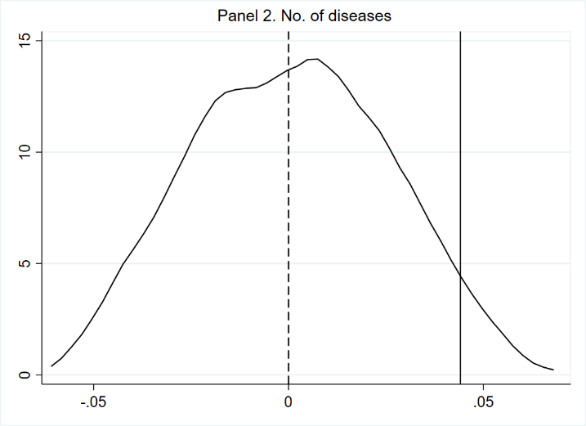
**

**S3 Fig. Placebo test.**

Supplement: S3 Fig — Notes: Panel 1 is the result of the ADL index placebo test; Panel 2 is the result of the placebo test of the number of patients. The X-axis represents the estimated coefficients from 1000 random assignments. The curve is the estimated kernel density distribution. The vertical type is a true estimate in columns (2) and (4) of Table 3. (DOCX) [file pone.0274027.s003.docx]
