# Supplementary material for: Agricultural non-point source pollution and health of the elderly in rural China
Source: PLoS One. 2022 Oct 14;17(10):e0274027. doi: 10.1371/journal.pone.0274027 (PMC9565375; doi:10.1371/journal.pone.0274027)
Supplement: S1 File — (DOCX) [file pone.0274027.s004.docx]

**S1 Table. Basica regression results without control variables.**

|  | ADL | | No. of diseases | |
| --- | --- | --- | --- | --- |
|  | (1) | (2) | (3) | (4) |
| Fertilizerloss*Time | 0.0175*** |  | 0.0052*** |  |
|  | (0.0057) |  | (0.0016) |  |
| Higharea*Time |  | 0.1608*** |  | 0.0441*** |
|  |  | (0.0432) |  | (0.0124) |
| Control | NO | NO | NO | NO |
| Year FE | YES | YES | YES | YES |
| ID FE | YES | YES | YES | YES |
| Observations | 32,302 | 32,302 | 32,467 | 32,467 |
| R-squared | 0.5474 | 0.5475 | 0.5597 | 0.5597 |

Notes:

*, **, *** are significant at the level of 10%, 5% and 1% respectively.

**S2 Table. Estimates of PSM-DID.**

|  | Nearest neighbor | | Radius | | Kernel | |
| --- | --- | --- | --- | --- | --- | --- |
|  | ADL | No. of diseases | ADL | No. of diseases | ADL | No. of diseases |
| Fertilizerloss*Time | 0.0695** | 0.0143** | 0.0267*** | 0.0065*** | 0.0267*** | 0.0065*** |
|  | (0.0273) | (0.0064) | (0.0085) | (0.0023) | (0.0085) | (0.0023) |
| Control | YES | YES | YES | YES | YES | YES |
| Year FE | YES | YES | YES | YES | YES | YES |
| ID FE | YES | YES | YES | YES | YES | YES |
| Observations | 4532 | 4532 | 17801 | 17801 | 17801 | 17801 |
| R-squared | 0.5927 | 0.7152 | 0.5828 | 0.7049 | 0.5828 | 0.7049 |

Notes:

*, **, *** are significant at the level of 10%, 5% and 1% respectively.

**S3 Table. Heterogeneous estimation of younger olds and oldest olds.**

|  | (1) | (2) | (3) | (4) |
| --- | --- | --- | --- | --- |
|  | ADL | | No. of diseases | |
|  | Younger olds | Oldest olds | Younger olds | Oldest olds |
| Fertilizerloss*Time | -0.0006 | 0.0356*** | 0.0033** | 0. 0086*** |
|  | (0.0038) | (0.0074) | (0.0014) | (0.0114) |
| Control | YES | YES | YES | YES |
| Year FE | YES | YES | YES | YES |
| ID FE | YES | YES | YES | YES |
| Observations | 20536 | 22367 | 20620 | 22431 |
| R-squared | 0.0761 | 0.1359 | 0.3418 | 0.2951 |
| p | 0.0008*** | | 0.0204** | |

Notes:

*, **, *** are significant at the level of 10%, 5% and 1% respectively.

**S4 Table. Heterogeneous estimation of younger olds, middle olds and oldest olds.**

|  | (1) | (2) | (3) | (4) | (5) | (6) |
| --- | --- | --- | --- | --- | --- | --- |
|  | ADL | | | No. of diseases | | |
|  | Younger olds | Middle olds | Oldest olds | Younger olds | Middle olds | Oldest olds |
| Fertilizerloss*Time | 0.0027 | 0.0000 | 0.0334*** | 0. 0018 | 0.0039* | 0. 0074*** |
|  | (0.0040) | (0.0061) | (0.0071) | (0. 0022) | (0. 0021) | (0.0114) |
| Control | YES | YES | YES | YES | YES | YES |
| Year FE | YES | YES | YES | YES | YES | YES |
| ID FE | YES | YES | YES | YES | YES | YES |
| Observations | 8332 | 10719 | 23852 | 8360 | 10769 | 23922 |
| R-squared | 0.0548 | 0.0885 | 0.1287 | 0.3416 | 0.3398 | 0.2954 |
| p | P^12^=0.7163 | | | P^45^=0.4969 | | |
|  | P^23^=0.0039*** | | | P^56^=0.1936 | | |
|  | P^13^=0.0029*** | | | P^46^=0.0419** | | |

Notes:

*, **, *** are significant at the level of 10%, 5% and 1% respectively.

**S5 Table. Robustness checks of fertilizer input and fertilizer input areas.**

|  | ADL | | No. of diseases | |
| --- | --- | --- | --- | --- |
|  | (1) | (2) | (3) | (4) |
| Fertilizer input*Time | 0.0010*** |  | 0.0001* |  |
|  | (0.0003) |  | (0.0001) |  |
| Higharea*Time |  | 0.2168*** |  | 0.0061 |
|  |  | (0.0547) |  | (0.0148) |
| Control | YES | YES | YES | YES |
| Year FE | YES | YES | YES | YES |
| ID FE | YES | YES | YES | YES |
| Observations | 32443 | 32278 | 32443 | 32443 |
| R-squared | 0.6905 | 0.5646 | 0.6905 | 0.6903 |

Notes:

*, **, *** are significant at the level of 10%, 5% and 1% respectively.

**S6 Table. Additional robustness checks of fertilizer loss in different areas.**

|  | Major Agri-producing areas | | Major Rice-producing areas | | Water resource rich areas | |
| --- | --- | --- | --- | --- | --- | --- |
|  | ADL index | No. of diseases | ADL index | No. of diseases | ADL index | No. of diseases |
| Fertilizerloss*Time | 0.0283*** | 0.00200 | 0.0203*** | 0.00500*** | 0.0274 | 0.0161*** |
|  | (0.0082) | (0.0021) | (0.0066) | (0.0017) | (0.0175) | (0.0043) |
| Control Variables | YES | YES | YES | YES | YES | YES |
| Year FE | YES | YES | YES | YES | YES | YES |
| ID FE | YES | YES | YES | YES | YES | YES |
| Observations | 16880 | 16959 | 21528 | 21603 | 20526 | 20604 |
| R-squared | 0.572 | 0.695 | 0.549 | 0.677 | 0.533 | 0.667 |

Notes:

*, **, *** are significant at the level of 10%, 5% and 1% respectively.

**S7 Table. Additional robustness checks of fertilizer loss areas in different areas in different areas.**

|  | Major Agri-producing areas | | Major Rice-producing areas | | Water resource rich areas | |
| --- | --- | --- | --- | --- | --- | --- |
|  | ADL index | No. of diseases | ADL index | No. of diseases | ADL index | No. of diseases |
| Higharea*Time | 0.255*** | 0.0389** | 0.163*** | 0.0452*** | 0.144** | 0.0339** |
|  | (0.0677) | (0.0171) | (0.0567) | (0.0141) | (0.0613) | (0.0158) |
| Control Variables | YES | YES | YES | YES | YES | YES |
| Year FE | YES | YES | YES | YES | YES | YES |
| ID FE | YES | YES | YES | YES | YES | YES |
| Observations | 16880 | 16959 | 21528 | 21603 | 20526 | 20604 |
| R-squared | 0.571 | 0.695 | 0.549 | 0.677 | 0.532 | 0.667 |

Notes:

*, **, *** are significant at the level of 10%, 5% and 1% respectively.

1. The fertilizer loss is a continuous variable, and we dichotomized fertilizer loss by its pre-policy mean to compare the health effect in different regions. The average value of fertilizer loss is the national average of fertilizer loss; it is equal to 5.36kg/ha. High loss areas include Anhui, Shandong, Jiangsu, Guangdong, Hebei, Henan, Zhejiang, Hubei and Fujian, while low loss areas include Chongqing, Sichuan, Shaanxi, Guangxi, Hunan, Jiangxi, Heilongjiang, Jilin, Liaoning and Shanxi. In this paper, data from Beijing, Tianjin and Shanghai are deleted because of the small sample size and the small agricultural planting area.

2. Data source of loss coefficient: technical report of the first general survey of pollution sources.

3. The major agricultural-producing areas are Hebei Province, Inner Mongolia Autonomous Region, Heilongjiang Province, Jiangsu Province, Anhui Province, Shandong Province, Henan Province, Hubei Province, Hunan Province and Sichuan Province.

The major rice-producing areas are Heilongjiang Province, Jiangsu Province, Anhui Province, Jiangxi Province, Hubei Province, Hunan Province, Guangdong Province, Guangxi Zhuang Autonomous Region, Chongqing and Sichuan Province.

The water resource rich areas are Heilongjiang Province, Zhejiang Province, Anhui Province, Fujian Province, Jiangxi Province, Hubei Province, Hunan Province, Guangdong Province, Guangxi Zhuang Autonomous Region and Sichuan Province.
