# Supplementary material for: Agricultural non-point source pollution and health of the elderly in rural China
Source: PLoS One. 2022 Oct 14;17(10):e0274027. doi: 10.1371/journal.pone.0274027 (PMC9565375; doi:10.1371/journal.pone.0274027)
Supplement: Attachment — Submitted filename: PONE-D-21-34489_R1_reviewer.pdf [file pone.0274027.s007.pdf]

PLOS ONE

Agricultural Nonpoint-source Pollution and Health of the Elderly in Rural China

--Manuscript Draft--

**Manuscript Number:**	PONE-D-21-34489R1
**Article Type:**	Research Article
**Full Title:**	Agricultural Nonpoint-source Pollution and Health of the Elderly in Rural China
**Short Title:**	none short title
**Corresponding Author:**	YING WANG<br>Nanjing Agricultural University College of Economics and Management<br>Nanjing, CHINA
**Keywords:**	chemical fertilizer loss, aging, health risk, difference-in-differences
**Abstract:**	Large input and high loss of chemical fertilizer are the main causes of agricultural non-point source pollution in China. Employing fertilizer loss and micro-health data, this paper analyzes the effects of chemical fertilizer loss on the health of rural elderly and the medical cost in China. Results of the difference-in-differences (DID) method indicate that one kg/ha increase in fertilizer loss alters a key medical disability index (Activities of Daily Living) by 0.0147 and the number of diseases by 0.0057 for rural residents 65 and older. This is equivalent to CNY 316 million (USD 45 million) in national medical cost. Furthermore, the age of onset is younger in regions with higher fertilizer loss. One kg/ha increase of fertilizer loss advances the age of onset by 0.267 year, which will cause long term effect on public health. Our results are robust to variety of robustness checks.
**Order of Authors:**	Ying WANG
	Hang XIONG
	Chao CHEN
**Response to Reviewers:**	Response to Editor<br>Dear Dr. Qiu,<br><br>Thank you for coordinating, reviewing, and extending the invitation to revise our manuscript. We have treated your as well as the reviewers' comments seriously and have significantly modified portions of the manuscript. For your advice, we have paid specific attention to meeting PLOS ONE's style and reporting associations in our manuscript. We summarize revisions we have implemented following the two reviewers' comments. Point by point responses are in separate files addressing to them respectively.<br><br>For Reviewer1, we have:<br>1.Improved writing and readability by simplifying some sentences, proving a better expression, and adding additional reading aid.<br>2.Added a series of publications in introduction and strengthened our contributions by comparing closely related literatures.<br>3.Enriched the content of empirical model of by adding regression results.<br><br>For Reviewer2, we have:<br>1.Compared the heterogeneous health risk of environmental exposure by adding the empirical analysis of different age groups and discussion in more detail.<br>2.Explained the hypotheses more clearly and explained the agricultural support polices in more detail.<br>3.Revised the description of the regression coefficients.<br>4.Offered the parallel trend assumption and discussed consistent estimation of Difference in Differences with a Continuous Treatment.<br>5.Improved writing and readability by including an additional figure, citing more recent publications, offering better variable definitions, and adding additional reading aid.<br><br>We hope you find our revised manuscript acceptable for publication in PLOS ONE.

Powered by Editorial Manager® and ProduXion Manager® from Aries Systems Corporation

Sincerely,

Ying Wang

Reviewer: 1
Thank you for the constructive comments on our manuscript. We have taken your comments seriously and substantially modified portions of the manuscript. We believe our work has been significantly improved as a result of your comments. Please find below our response to each point you made with your original comments in italic.

This paper focuses on an important and interesting topic of fertilizer exposure/application and human health. Although many efforts are put into this work, the paper still needs some improvement regarding methodology and organization/formatting of the paper. The writing and language should also be improved significantly before being published.

General comments
1. At the beginning of the introduction, a paper usually starts with background information and introduces the issue addressed by the paper. The first paragraph is not clear and interesting enough by listing some findings.
Response：Following your advice, we have significantly revised the first paragraph. First we introduced the background of agricultural non-point source pollution and fertilizer use in China. Second, added more publications to explain health effect of the agricultural non-point pollution Third, we explained the elders would be more susceptible, and the background of aging in China. This was the reason we focused on the elderly residences in rural China. Last, we have strengthened our contributions by comparing with two works most closely related to our study. The first paragraph was clearer and more logical.

2. Line 80 The contribution of the paper should be backed up with a thorough literature review. Please add more references and discussion on previous studies that are closely relevant to your study.
Response: Thank you very much for your suggestion. We have added some references about agricultural non-point pollution on public health, i.e. pesticide and straw burning in Line41-42, Line52-53. We have cited previous studies relevant to our study to back up the contribution of this paper in Line54-59.

3. Line 98 Can you have a descriptive map showing the provinces and use different color shades to show the levels of fertilizer application/exposure or heath level?
Response: Thank you very much for your suggestion. We dichotomized fertilizer loss by its pre-policy mean. The national average value of fertilizer loss is 5.36 kg/ha. The red parts in Figure 1 represent regions with high intensity of fertilizer loss and the pink parts represent are for regions with low intensity of fertilizer loss.

Fig 1 Fertilizer Loss in China

4. Line 105 What are other regional variables?
Response: Thank you very much for your suggestion. The other regional variables contain fertilizer use, organic fertilizer use, pesticide use, industrial pollution index, numbers of hospital and provincial Gross Domestic Product. GDP measures general economy and wealth conditions. The number of hospital controls for the level of medical service. Industrial pollutions control for pollution from industrial activities. All the regional variables are described in section “Provincial variables” in Section 2.1.3 “Provincial variables”.

5. Line 152 Why use the average of the past three years instead of using data from the same year? Is there a possibility of lagged effects?
Response: The consideration is as following. First, The individual health data from the

Powered by Editorial Manager® and ProduXion Manager® from Aries Systems Corporation

Chinese Longitudinal Healthy Longevity Survey. We observed were once every three years. Second, as mentioned in our article, most studies indicated a lagged effects of environmental exposure on public health(e.g. Wang and Luo 2021; Hu et al. 2015) Therefore, we follow the literature and also use the average of the past three year considering the lagged effect as well as the consistency of health data.

6. Line 155 I suggest adding min. and max to the table.
Response: Thank you for the advice. We have added the min. and max in Table1.

7. Table 1 Provincial variables. Would there be some city-level factors omitted such as city-level subsidies that will influence both fertilizer application and human health?
Response: Thank you very much for your suggestion. This study explores the health effect of fertilizer loss at the provincial level and we have controlled the individual effects, so we did not control the city-level factors. We did not control the city-level subsidies because the samples are rural residents, not only the farmers who receive the health damage via direct exposure. We hold the fertilizer loss is exogenous.

8. Line 165 “Lines 1 and 2”, do you mean rows?
Response: Thank you for the suggestion. “Line1 and 2” means the rows of ADL index and No. of diseases, respectively. “Rows or Lines” maybe nor clearly enough, so we reinterpret the results of Table 2 in Section 2.2.2.

9. Line 171 What are the average values of fertilizer loss?
Response: The fertilizer loss is continuous variable, and we dichotomized fertilizer loss by its pre-policy mean to compare the health effect in different regions. The average value of fertilizer loss is the national average of fertilizer loss; it is equal to 5.36kg/ha. We explained the details in section “Supporting information”.

10. Line 165-Line 176 Please give more background information about the agricultural subsidy policy. What it is about and how does it impact fertilizer loss?
Response: Thank you for the suggestion. We have added the background about agricultural subsidy policy and the effect of fertilizer loss on environment and public health in Section 2.2.2.

11. Could you have a graph showing how the intensity changes over the years for both the high areas and low areas?
Response: Thank you for the suggestion. The fertilizer loss intensity and intensity variations are shown in Fig 2. Fertilizer loss increase per hectare in high loss regions is roughly 2times larger than low loss areas. The variations of fertilizer input intensity, fertilizer loss and fertilizer loss intensity were showed in Table2 in the revised manuscript.

Fig 2 Fertilizer Loss Amount and Variation

12. This comparison of high and low is not exactly a difference-in-differences comparison because they both have the policy in place, and they are both “treated”. This is more a two-way fixed effects methodology.
Response: Thank you for the suggestion. As you pointed out, all the samples are treated by agricultural support policies. A difference-in-differences is still applicable, but with two conditions need to be satisfied. First, the impacts are different between “experience group” and “control group”. Second, the parallel trend assumption is need be hold. Following publications adopted DID to explain the “policy effect”, however all samples were treated by the “policy”: the bill of strengthening the protection of mortgage claims will have great impact on enterprises with high proportion of tangible assets, because the tangible assets are easier to be used as collateral, so Vig(2013) constructed the experimental group and the control group according to the proportion of tangible assets of enterprises. Campello &Larrain (2016) constructed the experimental group and control group according to the difference of enterprises'

Powered by Editorial Manager® and ProduXion Manager® from Aries Systems Corporation

demand for movable property. In our study, we constructed the experimental group and control group by fertilizer loss levels. First, the parallel trend assumption holds. Second, most of the high loss areas are in the developed regions and is the primary rice production regions in China. Agricultural subsidies of per unit of field are different between provinces, such as the subsidies in Jilin province is four and two times lower than Guangdong and Zhejiang respectively (Shu et al.,2013). The developed provinces had a larger subsides, and this will cause a larger fertilizer loss after agricultural support policies. The Green Revolution lead to the extensive use of chemical fertilizer, particular in rice production. We dichotomized fertilizer loss by its pre-policy mean. The variation of fertilizer loss in high loss areas is 2 times larger than low loss areas. Also, we used a continuous measure of intensity of treatment (i.e. fertilizer loss) and thereby captured more variation in the data (Nunn and Qian, 2011; Lai, 2017). As your suggestion, we have estimate the health effect of fertilizer loss using TWFE, and we have added the estimated results in column (3) and column (6) of Table3.

13. Line 179 Table notes should be self-explanatory. The readers could understand without referring too much to the paper.
Response: Thank you for your advice. We have deleted the table notes.

14. Equation (4) There is no subscript for X. Will heavy metals from organic fertilizer also impact human health? Will that bias your estimates?
Response: Thank you for the suggestion. We have added the subscript for X in Equation (4). Chemical fertilizer was most widely used in China and the supply proportion of chemical fertilizer nutrients exceeds seventy percent. The use of organic fertilizer was far less than that of chemical fertilizer, and the organic fertilizer use was concentrated in some regions. So we did not consider the health effect of organic fertilizer. But following your suggestion, we have controlled the organic fertilizer use in our revised manuscript.

15. Line 216 How old is defined as rural elderly and “younger elderly”? Which part of the results is based on equation (4) and which is from equation (5)?
Response: Thank you for the suggestion. According to the previous studies, an elderly person over 80 years or 85 years is defined as the oldest olds. The oldest olds were wildly discussed because they had greater risk of falling and hospitalization (Rockwood et al. 1999; Speechley &Tinetti, 1991; Winograd,1991; Brettschneider et al. 2019). We divided the samples by age into the younger elderly samples (<80years) and the oldest olds samples (≥80 years). The coefficient of equation (4) represented the post-agricultural policies of fertilizer loss. health effect of fertilizer loss and the   of equation (5) captured the post-agricultural policies effect of high loss areas relative to low loss areas. Estimated coefficients of   were reported in column (1) and column (3) of Table3, and estimated coefficients of   were reported in column (2) and column (4) of Table3. We described the result of Table 3 in section 3.1.1.

16. Line 232 Policy implications can be put in the conclusion section.
Response: Thank you for this advice. We have deleted the policy implications Line229-235, Line261-257 of original manuscript. And we discussed the policy implications in conclusion section.

17. Table 3 What are the coefficients of the covariates (control variables)? Do they show the expected sign? What are the results without control variables?
Response: We have added the results without control variables in S1 Table in section “Support information” in our revise manuscript. The results with covariates are shown in Table 1 below.
Table 1 Basic Estimates ADL index and Diseases
ADLNo. of diseases
(1)(3)(2)(4)
Fertilizerloss*Time0.0147**0.0057***
(0.0061)(0.0014)
Higharea*Time0.1222***0.0487***
(0.0474)(0.0116)
Floss0.05700.0659-0.0148-0.0124

Powered by Editorial Manager® and ProduXion Manager® from Aries Systems Corporation

(0.0417)(0.0416)(0.0103)(0.0103)
Pinpur-94.2811-125.0235-47.6648-65.0932**
(116.5155)(118.8320)(30.3544)(31.1875)
OFinput 0.00100.00100.0009***0.0009***
(0.0010)(0.0010)(0.0002)(0.0002)
Age0.02730.03230.01130.0109
(0.0318)(0.0317)(0.0081)(0.0081)
Male -0.0103-0.0373-0.0029-0.0042
(0.2373)(0.2385)(0.0426)(0.0427)
Co-residence0.1617***0.1647***-0.0074-0.0077
(0.0256)(0.0256)(0.0066)(0.0066)
Income_cost0.0354*0.0271-0.0024-0.0023
(0.0200)(0.0199)(0.0046)(0.0046)
Illness1.5128***1.4911***3.5079***3.5068***
(0.1930)(0.1923)(0.0580)(0.0581)
Hospitalization0.1171***0.1170***0.0126***0.0126***
(0.0183)(0.0183)(0.0038)(0.0038)
Smoke-0.0784-0.07220.0369*0.0366*
(0.0856)(0.0851)(0.0201)(0.0201)
Drink -0.0330-0.0264-0.0104-0.0103
(0.0663)(0.0662)(0.0167)(0.0167)
Exercise-0.2192***-0.2181***-0.0123-0.0123
(0.0489)(0.0488)(0.0127)(0.0127)
Meat0.01960.00440.00560.0054
(0.0140)(0.0184)(0.0044)(0.0044)
Fish0.0245*0.0132-0.0014-0.0015
(0.0131)(0.0184)(0.0045)(0.0045)
Egg -0.0140-0.02470.00240.0025
(0.0116)(0.0183)(0.0046)(0.0046)
Salt_veg-0.0435***-0.0406***-0.0075***-0.0075***
(0.0089)(0.0089)(0.0024)(0.0024)
Boild water-0.0836*-0.0934**-0.0042-0.0050
(0.0456)(0.0457)(0.0107)(0.0107)
Water0.00280.0027-0.0152-0.0147
(0.0823)(0.0822)(0.0175)(0.0175)
Hospnum0.0712***0.0706***-0.0155***-0.0159***
(0.0153)(0.0154)(0.0041)(0.0041)
LnGDP-0.2306-0.23790.09200.0996*
(0.2207)(0.2185)(0.0585)(0.0576)

Indpolltion0.06620.04950.02910.0279
(0.1085)(0.1081)(0.0253)(0.0253)
Constant4.88445.0608-1.6139*-1.6166*
(3.3262)(3.3242)(0.8570)(0.8543)
Observations32,16532,16532,32932,329
R-squared0.55700.55820.69060.6907
Year FEYESYESYESYES
ID FEYESYESYESYES

robust standard errors for clustering at the individual level are reported in brackets; *, **, *** are significant at the level of 1%, 5% and 10% respectively

18. Line 248 What do you mean by “reduces the age of the elderly”? Please pay attention to how to interpret the results.
Response: Thank you for the advice. “Reduces the age of elderly” means the fertilizer loss will lead the age of illness onset younger. As your suggestions, we have deleted this confusing expression and interpreted the results of Table 4 in section 3.1.3.

19. Line 276 How are the costs of fertilizer application compared to the cost of this pesticide use?
Response: Thank you for the suggestion. This study focused on the health effect and medical cost loss of fertilizer. We have estimated the medical cost of fertilizer loss. An

Powered by Editorial Manager® and ProduXion Manager® from Aries Systems Corporation

increase of one unit (1kg/ha) of fertilizer loss would add CNY 3.74 (USD 0.53) to medical cost, which would increase 316 million yuan (45 million dollars) in total for the rural elderly populations in China. For you advise, we added medical cost of fertilizer input in Line290-293. One more unit fertilizer input will increase medical cost by CNY 0.22. For the elderly population in rural China, the total medical cost will increase CNY 21.5 (USD 3.1) million.

20. Line 301 Do you mean equations (4) and (5)?
Response: Sorry about these errors. We have corrected these errors.

21. Line 317 What are these rice-producing areas. Could you be more specific?
Response: Thank you for the suggestion. The details about major agricultural-producing areas, major rice-producing areas and water resource-rich areas are in section supporting information.

22. Formatting:
Introduction. Each Line ends with a half-word. I suggest checking the composition/distribution of paraphs.
Response: Thank you for this suggestion. We have revised it in introduction and other parts of our manuscript.

23. "This is table X legend." What is this for? Considering removing. Please also check the table adjustment and Line spacing.
Response: Thank you for the suggestion. "This is table X legend" is the template format. We have adjusted the single spacing of the tables.

24. Grammar mistakes and typos:
There are grammar mistakes and typos. Please also check the uses of some phrases. Examples are seen in the following, but not limited to:
First paragraph in the introduction, "stats, surmise"
Line 56 Plication
Line 100 "the data of 1998…."
Line 127 "rejuvenation of disease", "income station
Line 146 should this n be h?
Line 155 Indicaos should be indicators
Line 165 "after the implementation" duplicated phrases
Line 201 medic should be medical
Line 202 than should be then
Some sentences can be cleaned and simplified. I encourage the authors to have some native or fluent speakers read through and help with the language of the paper.
Response: Sorry for these errors. In our revise manuscript, we have carefully checked the grammar and revised the sentences.

Reviewer: 2
Thank you for the constructive comments on our manuscript. We have taken your comments seriously and substantially modified portions of the manuscript. We believe our work has been significantly improved as a result of your comments. Please find below our response to each point you made with your original comments in italic.

This paper focuses on an important and interesting topic of fertilizer exposure/application and human health. Although many efforts are put into this work, the paper still needs some improvement regarding methodology and organization/formatting of the paper. The writing and language should also be improved significantly before being published. At the beginning of the introduction, a paper usually starts with background information and introduces the issue addressed by the paper. The first paragraph is not clear and interesting enough by listing some findings.

Powered by Editorial Manager® and ProduXion Manager® from Aries Systems Corporation

General comments

1. Although it is important to examine the regional inequality in the medical outcome, the most important question is: why aged people? Previous studies show that fertilizer loss damages residents, then the natural steps should be who are more subjected to harm from fertilizer loss. Therefore, citing a study to show the difference between elder residents and younger residents is appreciated.

Response：Thank you for the suggestion. China has the most serious aging, 70% of elderly live in rural areas. Agricultural non-point pollutions had been more serious according to the overuse of agrochemicals. Under the background of aging and non-point pollution, this study focused on health effect of fertilizer loss on elderly in rural areas. The elder residents have different vulnerabilities to environmental contaminants. We have cited several studies in Line 44-48.

2. Therefore, hypothesis 1 needs transition. If fertilizer loss is harmful to health, in general, is known, why do we have to explore its effect on aged residents, before showing whether there is a difference between elder and younger residents? Are they more vulnerable or more resilient than the younger group? This problem can be solved by citing studies showing fertilizer overuse has a different effect on elder and younger people.

Response: Several studies had reported that the elder residents were more vulnerable than the younger groups. A higher susceptibility and higher mortality rates could be observed among the elder residents (We cited the studies in Introduction(Line44-45). We have added more references to explain the reasons of heterogeneity effect in younger and older population in Line 45-48. We further explored heterogeneous health effect of fertilizer loss by splitting the samples in various ages. First, we divided the samples at age into the younger age samples (<80years) and the oldest olds samples (≥80 years). Then, we also divided the samples into younger olds (65years-75years), middle olds (75-85years) and oldest olds (≥85years) according to Monti et al. 2021 and Lau et al. 2021. The heterogeneous effects are in section 3.1.2.

3. For hypothesis 2, you might want to add more explanation on how the outcome variables are correlated with the "rejuvenation of disease". How do you define the term?

Response: We have rewired the hypothesis 2. Because of the physical function, fertilizer loss may lead the samples to be ill younger. The fertilizer loss caused the several diseases, which in Line76-83. And in Figure 2, the elderly under 80 years of age, there was a significant difference in the age of illness onset in the areas with high chemical fertilizer loss, and the age of illness onset in the elderly in the areas with high chemical fertilizer loss was lower than that in the areas with low chemical fertilizer loss.

4. Hypothesis 3 makes two points, and should be separated; their contents are also made sense by common wisdom, but the literature in the manuscript seems not to correlate with them.

Response: Thank you for the suggestion. We revised the hypothesis 3 by "Due to the health damages, the medical cost will be higher in high-loss regions comparing the regions with lower fertilizer loss". In this section, we explored the economic results by translating estimated effects into expected monetary loss.

5. The linear regression coefficients are correlations, not "impact". The impact has a causal implication.

Response: Thank you for the suggestion. We have revised the expressions by using "lead to, be associated with".

6. This research uses DID, but the research did not check the parallel trends before 2004. In this study, the main analysis uses data after 2004 and separates the provinces into high-loss and low-loss. However, as far as I am aware, there is no mention of how the health outcomes trend before 2004. For DID, parallel trends assumption is essential. I randomly search for a blog from the world bank that could help the authors to have the idea

Powered by Editorial Manager® and ProduXion Manager® from Aries Systems Corporation

	(https://blogs.worldbank.org/impactevaluations/revisiting-difference-differences-parallel-trends-assumption-part-i-pre-trend). Your placebo test does not correlate with this problem.<br>Response: Thank you for the suggestion. We added the parallel trends in section 3.3.1. We divided the samples to six sections: the second period before the policy implementation, the first period before the policy implementation, the second period after policy, the second two periods after the policy implementation to the end of sample period. Thus, six indicator variables pre_2, pre_1, post_1, post_2, post_3 and post_4 were constructed. The set of pre_1 coefficient were statistically indistinguishable from zero, but there was a sharp, statistically significant and sustained change after the policy implementation.<br><br><br>7. Even if the parallel trend assumption holds, another problem is how to support the assumption that all the residents are treated to high fertilizer overuse randomly. High fertilizer loss could also imply a lack of management or facility, which also leads to high medical loss. For example, it can be argued that the aged person exposed to extra use of fertilizer tend to live in provinces with lower medical resources (agriculture province such as Hubei are also subjected to fertilizer overuse). In short, the author should consider blocking potential causal paths.<br>Response: Thank you for the suggestion. We had considered the randomization of fertilizer loss. A major concern was that the factors that caused the different fertilizer loss between provinces may be correlated with health outcomes. These types of confounding factors vary across provinces and individuals but are fixed over time. A common method of controlling for time-invariant unobserved heterogeneity is to use fixed effects. We had fixed the individual effect and time effect. And we also had controlled the medical resources and economic developments.<br><br><br>8. Another problem that comes with DID is the treatment effect. In formula (4), it seems the treatment effect is continuous. However, the traditional DID does not allow for the continuous treatment effect and could miscalculate S.D and perform the wrong t-test. I suggest reading this paper and reconsidering this part:<br>Callaway, B., Goodman-Bacon, A., & Sant'Anna, P. H. C. (2021). Difference-in-Differences with a Continuous Treatment. ArXiv:2107.02637 [Econ]. http://arxiv.org/abs/2107.02637<br>Response: Thank you for this advice and thank you very much for recommending the paper. We have learned the paper you recommended carefully, the paper provided a good job in the front of theoretical basis research of Difference-in-Differences. Unfortunately to date a package that allows researchers to implement this approach in Stata has not been released. And sorry for that we are not capable of empirical research following Callaway's theory. However, if differences in treatment intensity are not correlated with heterogeneity in the treatment effect at a given treatment intensity or there is no selection of outcome variables, the traditional DiD can be used (Callagher, et al. 2021; Kindsgrab, 2022; Battiston et al.2021). Callaway argued that the magnitude of coefficient with continuous treatment from DiDs should be interpreted with caution as the interaction coefficient identifies a weighted average of the "average causal response" of the treatment along different levels of the treatment. In our setting, we are interested in causal parameter the average treatment effect to fertilizer loss level . When we want to compare the magnitudes of across different levels of health damage (i.e. Equation4 and Equation6), we need a stronger parallel trends assumption that the restricts the potential outcomes of individuals. Specifically, we must assume that the average potential outcomes for individuals are the same at each level of fertilizer loss, or that (on average) there is no selection into a particular level of fertilizer loss. The stronger parallel trends assumption is likely to be satisfied in our setting. In addition, we followed some literatures by adopting a binary treatment as robustness check (Gong et al.2022; Motohashi,2022).<br><br><br>Minor problems are listed as follows:<br>9. Replacing the word "rejuvenation of disease". Many definitions can be found on the internet, so it might be helpful to address this word.<br>Response: Thank you for thesuggestion. In this study, "rejuvenation of disease" means that the residences may be ill in younger ages. We have changed the expression by

Powered by Editorial Manager® and ProduXion Manager® from Aries Systems Corporation

"be ill younger" and "be sick in younger age".

10. The research can be more specific on the unit of analysis. "Matches micro-level individual health data and provincial-level chemical fertilizer" is confusing.
Response: Thank you for the advice. The analysis was accomplished using difference in differences method with the national dataset Chinese Longitudinal Healthy Longevity Survey (CLHLS) and publicly published yearbooks. We have revised the explanation in first paragraph of Section 2.1 "Data Collection and Variables". We have introduced the CLHLS database and how did the individual variables defined in section 2.1.1 and section 2.1.2. We also explained the provincial variables in section 2.1.3.

11. No need to discuss policy implications in basic regression results of health. I was referring to the line(L)229-L235, L261-257,
Response: Thank you for the suggestion. We have deleted the discussion of policy implications.

12. When you use "1% level is significant", do you mean "significant at 99% CI"?
Response: Thank you for the suggestion. Yes, "1% level is significant" means "significant at 99% CI". "1% level is significant" is usually used in economics.

13. L316-L319: Are you referring to table 6 or table 7?
Response: Sorry for these errors. Line316-319 is the descriptions of Table 7. And we have moved Table7 to section "Supporting information", in S3 Table and S4 Table.

14. Results of the placebo test, the Effect of fertilizer input on health, and the Effect in different areas can be moved to the appendix.
Response: Thank you for the suggestion. We have moved the robustness check to the Supporting information.

To sum up, in its current form, this manuscript is not ready to publish. However, if the author is willing to address these mentioned problems, then the topic is interesting and contributes to the existing literature.

Reference

1.Battiston D, Espinosa M, Liu S. Talent poaching and job rotation. SSRN. 2021; 3778068,
2.Brettschneider C, Hajek A, Rohr S, Fuchs A, Weeg D, et al. Determinants of health-care costs in the oldest-old in Germany. The Journal of the Economics of Ageing. 2019; 14: 100200.
3.Campello M, Larrain M. Enlarging the contracting space: Collateral menus, access to credit, and economic activity. The Review of Financial Studies. 2016; 29(2): 349-383.
4.Gallagher J, Hartley D, Rohlin S. Weathering an unexpected financial shock: the role of federal disaster assistance on household finance and business survival. 2021.
5.Gong D, Yan A, Yu J. Cost of zero-covid: effects of anti-contagious policy on labor market outcomes in china[J]. SSRN.2022; 4037688.
6.He GJ, Liu T, Zhou MG. Straw burning, PM 2.5, and death: evidence from China. Journal of Development Economics. 2020;145: 102468.
7.Hu R, Huang X, Huang J, Li Y, Zhang C, et al. Long- and short-term health effects of pesticide exposure: a cohort study from China. PLOS One. 2015; 10(6): e0128766.
8.Kindsgrab P. Do higher income taxes on top earners trickle down? A local labor markets approach. A Local Labor Markets Approach, 2022.
9.Lai WY. Pesticide use and health outcomes: evidence from agricultural water pollution in China. Journal of Environmental Economics and Management. 2017; 86: 93-120.

Powered by Editorial Manager® and ProduXion Manager® from Aries Systems Corporation

10.Motohashi K. Unintended consequences of sanitation: externalities on water quality and health in India.2022.
11.Nunn N, Qian N, The potato's contribution to population and urbanization: evidence from a historical experiment. The Quarterly Journal of Economics. 2011;126(2): 593-650.
12.Rockwood K, Stadnyk K, MacKnight C, McDowell I. A brief clinical instrument to classify frailty in elderly people. The Lancet. 1999; 353:205-206.
13.Shu KL, Yang S, Wang HL, Guo QH. Analysis on evolution of Jilin grain production investment mechanism and its relevant policy effect. Research of Agricultural Modernization. 2013;34(03): 257-262.
14.Speechley M, Tinetti M. Falls and injuries in frail and vigorous community elderly persons. Journal of the American Geriatrics Society. 1991; 39:46-52.
15.Wang Y Z, Luo N S. Air pollution, health depreciation and medical costs: Research based on the three perspectives of physical health, mental health and social adaptability[J]. Economic Research Journal, 2020, 55(12): 80-97.
16.Winograd CH. Targeting Strategies: An overview of criteria and outcomes. Journal of the American Geriatrics Society. 1991; 39:25-35.
17.Vig V. Access to collateral and corporate debt structure: evidence from a natural experiment. The Journal of Finance. 2013; 68(3): 881-928.

Additional Information:	
**Question**	**Response**
**Financial Disclosure**<br><br>Enter a financial disclosure statement that describes the sources of funding for the work included in this submission. Review the submission guidelines for detailed requirements. View published research articles from *PLOS ONE* for specific examples.<br><br>This statement is required for submission and **will appear in the published article** if the submission is accepted. Please make sure it is accurate.	NO - Include this sentence at the end of your statement: The funders had no role in study design, data collection and analysis, decision to publish, or preparation of the manuscript.

**Unfunded studies**<br>Enter: *The author(s) received no specific funding for this work.*<br><br>**Funded studies**<br>Enter a statement with the following details:<br>• Initials of the authors who received each award<br>• Grant numbers awarded to each author<br>• The full name of each funder<br>• URL of each funder website<br>• Did the sponsors or funders play any role in the study design, data collection and analysis, decision to publish, or preparation of the manuscript?<br>• **NO** - Include this sentence at the end of your statement: *The funders had no role in study design, data collection and analysis, decision to publish, or preparation of the manuscript.*<br>• **YES** - Specify the role(s) played.<br><br>* typeset	
**Competing Interests**<br><br>Use the instructions below to enter a competing interest statement for this submission. On behalf of all authors, disclose any competing interests that could be perceived to bias this work—acknowledging all financial support and any other relevant financial or non-financial competing interests.<br><br>This statement is required for submission and **will appear in the published article** if the submission is accepted. Please make sure it is accurate and that any funding sources listed in your Funding Information later in the submission form are also declared in your Financial Disclosure statement.<br><br>View published research articles from *PLOS ONE* for specific examples.	The authors have declared that no competing interests exist.

**NO authors have competing interests**<br><br>Enter: *The authors have declared that no competing interests exist.*<br><br>**Authors with competing interests**<br><br>Enter competing interest details beginning with this statement:<br><br>*I have read the journal's policy and the authors of this manuscript have the following competing interests: [insert competing interests here]*<br><br>* typeset	
**Ethics Statement**<br><br>Enter an ethics statement for this submission. This statement is required if the study involved:<br><br>• Human participants<br>• Human specimens or tissue<br>• Vertebrate animals or cephalopods<br>• Vertebrate embryos or tissues<br>• Field research<br><br>Write "N/A" if the submission does not require an ethics statement.<br><br>General guidance is provided below. Consult the submission guidelines for detailed instructions. **Make sure that all information entered here is included in the Methods section of the manuscript.**	N/A. The individual informations come from a national survey. The survey is about the elderly in 22 provinces of China. The respindents are willing to accept the survey, and the survey data can be used pubicly. The private information that can identify the respondents are hidden.

**Format for specific study types**<br><br>**Human Subject Research (involving human participants and/or tissue)**<br>• Give the name of the institutional review board or ethics committee that approved the study<br>• Include the approval number and/or a statement indicating approval of this research<br>• Indicate the form of consent obtained (written/oral) or the reason that consent was not obtained (e.g. the data were analyzed anonymously)<br><br>**Animal Research (involving vertebrate animals, embryos or tissues)**<br>• Provide the name of the Institutional Animal Care and Use Committee (IACUC) or other relevant ethics board that reviewed the study protocol, and indicate whether they approved this research or granted a formal waiver of ethical approval<br>• Include an approval number if one was obtained<br>• If the study involved *non-human primates*, add *additional details* about animal welfare and steps taken to ameliorate suffering<br>• If anesthesia, euthanasia, or any kind of animal sacrifice is part of the study, include briefly which substances and/or methods were applied<br><br>**Field Research**<br><br>Include the following details if this study involves the collection of plant, animal, or other materials from a natural setting:<br>• Field permit number<br>• Name of the institution or relevant body that granted permission	
**Data Availability**<br><br>Authors are required to make all data underlying the findings described fully available, without restriction, and from the time of publication. PLOS allows rare exceptions to address legal and ethical concerns. See the PLOS Data Policy and FAQ for detailed information.	Yes - all data are fully available without restriction

A Data Availability Statement describing where the data can be found is required at submission. Your answers to this question constitute the Data Availability Statement and **will be published in the article**, if accepted.<br><br>**Important:** Stating 'data available on request from the author' is not sufficient. If your data are only available upon request, select 'No' for the first question and explain your exceptional situation in the text box.<br><br>Do the authors confirm that all data underlying the findings described in their manuscript are fully available without restriction?	
**Describe where the data may be found in full sentences. If you are copying our sample text, replace any instances of XXX with the appropriate details.**<br><br>• If the data are **held or will be held in a public repository**, include URLs, accession numbers or DOIs. If this information will only be available after acceptance, indicate this by ticking the box below. For example: *All XXX files are available from the XXX database (accession number(s) XXX, XXX.).*<br>• If the data are all contained **within the manuscript and/or Supporting Information files**, enter the following: *All relevant data are within the manuscript and its Supporting Information files.*<br>• If neither of these applies but you are able to provide **details of access elsewhere**, with or without limitations, please do so. For example:<br><br>*Data cannot be shared publicly because of [XXX]. Data are available from the XXX Institutional Data Access / Ethics Committee (contact via XXX) for researchers who meet the criteria for access to confidential data.*<br><br>*The data underlying the results presented in the study are available from (include the name of the third party*	The data underlying the results presented in the study are available from https://opendata.pku.edu.cn/dataverse/CHADS;jsessionid=6a16c8c5d8c20925c51b93da42fc.

and contact information or URL).<br>• This text is appropriate if the data are owned by a third party and authors do not have permission to share the data.<br><br>* typeset	
Additional data availability information:	

Powered by Editorial Manager® and ProduXion Manager® from Aries Systems Corporation

Revised Manuscript Click here to access/download;Manuscript;Manuscript.doc

Agricultural Nonpoint-source Pollution and Health of the Elderly in Rural China

Ying WANG[1], Hang XIONG[2], Chao CHEN[3*]

[1] College of Economics and Management, Nanjing Agricultural University, Nanjing, Jiangsu, P.R.China

[2] College of Economics and Management, Nanjing Agricultural University, Nanjing, Jiangsu, P.R.China; China Center for Food Security Studies, Nanjing Agricultural University, Nanjing, Jiangsu, P.R.China

[3] College of Economics and Management, Nanjing Agricultural University, Nanjing, Jiangsu, P.R.China; China Center for Food Security Studies, Nanjing Agricultural University, Nanjing, Jiangsu, P.R.China

* Corresponding author

E-mail: cchen@njau.edu.cn.

Funding Information：This article was sponsored by the Priority Academic Program Development of Jiangsu Higher Education Institutions (PAPD)

Abstract

Large input and high loss of chemical fertilizer are the main causes of agricultural nonpoint-source pollution in China. Employing fertilizer loss and micro-health data, this paper analyzes the effects of chemical fertilizer loss on the health of rural elderly and the medical cost in China. Results of the difference-in-differences (DID) method indicate that one kg/ha increase in fertilizer loss alters a key medical disability index (Activities of Daily Living) by 0.0147 and the number of diseases by 0.0057 for rural residents 65 and older. This is equivalent to CNY 316 million (USD 45 million) in national medical cost. Furthermore, the age of onset is younger in regions with higher fertilizer loss. One kg/ha increase of fertilizer loss advances the age of onset by 0.267 year, which will cause long term effect on public health. Our results are robust to variety of robustness checks.

1. Introduction

China experienced rapid growth in chemical fertilizer use, particular after 2004 when the Chinese government shifted from taxing agriculture to subsidizing agricultural programs. The unit area chemical fertilizer usage is nowadays 4 times more than the world average [1]. Agricultural nonpoint-source pollution is a growing concern in China because these pollutions had become the main cause of water pollution [2]. Agricultural nonpoint-source pollution has been found to be an important factor affecting health [3-6]. Specifically, the elderly might have a higher health risk comparing with the young people when facing environmental exposure. A higher susceptibility and higher mortality rates could be observed among the adults over the age of 75 years compared with the younger ages [7-9]. The differences of health risk might due to physiological changes associated with aging [10]; the age-related behavior trends varied between younger and older adults which might affect older adults' exposures [11]; other factors such as socio-economic and nutritional status [12]. China had the most serious aging problems, 70% of elderly live in rural areas [13], the elderly residents might most affect by agricultural nonpoint pollution. Another reason

we focus on the rural elderly is that the health status, medical facility and diseases awareness of rural elderly are not as good as those in cities [14-15]. This paper complements the existing literature by proving new evidence on the effects of agricultural non-agricultural pollution. [illegible]r pollution as a leading problem for public health was broadly studied in agricultural nonpoint pollutions [16-17]. The works most closely related to this paper were Brained and Menon, which assessed the impact of fertilizer runoff on infant and child in India, we complement their study by emphasizing another vulnerable group, i.e. older adults, who are more likely to stay in countryside in the process of urbanization and most directly affected by the loss of chemical fertilizer. Another work is Lai, which assessed the health effect of pesticide by using the pesticide cost. In order to eliminate the influence of price, we use the amount of chemical input and loss, which are more accurate. Though both chemical fertilizers and pesticides could cause water pollution, they worked in different ways. Therefore, this study to explain the health effect of fertilizer loss is valuable. This paper emphasis on non-occupational fertilizer exposure, which is necessary because the farmers' occupational risk via direct exposure of chemical fertilizer was only a small proportion of the population and had been studied [18-20]. Many more population may be at risk because of agricultural externality.

The inefficient use of chemical fertilizer has caused serious nonpoint-source pollution. Fertilizers leaded to "groundwater contamination, eutrophication of fresh water and estuarine ecosystems, tropospheric pollution related to emissions of nitrogen oxides and ammonia gas, and accumulation of nitrous oxide" [21]. A 10% increase in the use of nitrogen fertilizer (kg) and phosphorus fertilizer(kg) leaded to a 1.525% and 1.374% increase of the concentration of nitrogen (mg/l) and phosphorus (mg/l) in water, respectively [22]. In China, agricultural nonpoint-source pollution has exceeded industrial pollution in water, the chemical oxygen demand (COD) emission of agricultural pollution sources was 1.8 times of the industrial sources [23]. After 2004, in particular, China abolished agricultural tax and issued a series of agricultural support policies to promote agricultural production, which encourage the excessive application of agricultural fertilizer. From 2004 to 2015, the amount of chemical fertilizer applications was increased by 30% (i.e., 1.386 million tons) [24], while the increase in agricultural produce output was far less. As a result of the formation of nitrite compounds and the change in soil environmental conditions, chemical fertilizer loss may

potentially lead to chronic diseases, nervous system diseases, and organ diseases [25-27], and eventually affect human health as well as medical cost (Fig 1). Amines and amides in chemical fertilizers and sewage could cause digestive tract diseases, esophageal cancer, liver cancer, stomach cancer, esophagus cancer, and other cancers [28-30]. The fertilizer, which were soluble in water and had stable properties, were transported by rivers over long distances and large ranges, and enter the human body via drinking water and other ways to synthesize nitrosamines, especially nitrosamines. Drinking water or eating vegetables with a high nitrate content may lead to thyroid cancer, hypertension, neural tube defects (NTDs) [31-32], and various chronic diseases, such as skin diseases, lung cancer, bladder cancer, and cardiovascular disease [33]. Based on the background of China's agricultural support policy, this paper verifies the effect of chemical fertilizer loss from the soil on the health of the rural elderly, and comprehensively analyzes the health damage from the perspectives of daily living ability, disease risk, and disease age onset. The economic cost of this health damage is further calculated. More specifically, 3 hypotheses are proposed as follow: (1) Fertilizer loss can cause physical damage, increase the health risk and alter the daily living ability of the elderly. (2) Because of the physical function, fertilizer loss may lead the s[illegible]ples to be ill younger. (3) Due to the health damages, the medical cost will be higher in high-loss regions comparing the regions with lower fertilizer loss.

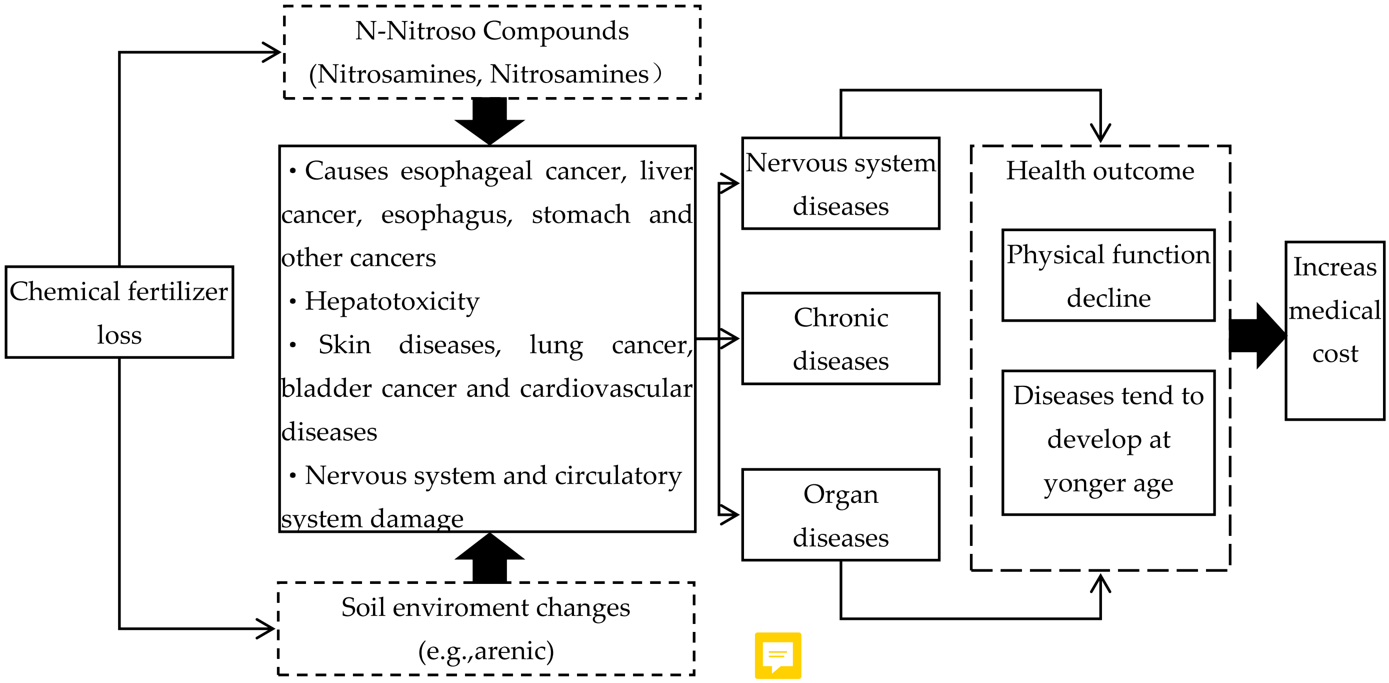

Fig1. Effects of Chemical Fertilizer Loss on Health.

The rest of the paper is organized as follows. Section 2 provides data description and summary statistics. Section 3 presents empirical models as well as results and Section 4 concludes.

2. Data and summary statistics

2.1 Data collection and variables

The analysis is accomplished using difference in differences method with the national dataset Chinese Longitudinal Healthy Longevity Survey (CLHLS) and publicly published yearbooks.

The individual health data come from the Chinese Longitudinal Healthy Longevity Survey (CLHLS). CLHLS is chosen for this research owning to its richness in health and demographic information, its nationwide sampling areas, and its time span which covers the year of the agricultural policy change. The CLHLS was conducted in a randomly selected half of the counties and cities in 22 provinces, covering 85% of the total population in China. Its eight waves (1998, 2000, 2002, 2005, 2008, 2011,2014 and 2018) surveyed the cohort of 65 years and older. We only use the data from 2000 to 2014 because the survey question asking whether drinking water was boiled or not started in 2000; only those who drank boiled water are included in our analysis to eliminate possible health linkages to waterborne bacteria. China began to implement the "fertilizer reduction" policy in 2015, in order to eliminate the policy effect, we did not use the data after 2015. The survey combines an in-home interview and a basic physical examination. Extensive information was collected on demographic characteristics, family and household characteristics, lifestyles, diet, psychological characteristics, health, disability, socioeconomic conditions, etc. Fertilizer input data come from the China Rural Statistical Yearbook (1999-2015) and other regional variables come from the China Statistical Yearbook (1999-2015). Chemical fertilizer loss index come from the Technical report on China pollution source census (2011).

2.1.1 Health outcomes variables

Multiple indicators for health status of the elderly are examined linked to fertilizer loss in the extant medical and toxicology literatures, including the index of Activities of Daily Living (ADL), the numbers of diseases related to fertilizer loss, and age of onset.

The ADL index is widely used to measure the dependence of the elderly and physically inactive people. It is also known as the "disability" index [34]. The ADL index is constructed by asking participants whether they need help with six basic activities (bathing, dressing, eating, going to the toilet, indoor movement, and controlling defecation). The choices for each item are "can do without help" (corresponding score is 1), "need help" (corresponding score is 2), and "need full help" (corresponding score is 3). The final score of the ADL index is the sum of all six items, ranging from 6 to 18. Higher scores mean the health status of the elderly is worse.

The number of diseases measures how many types of diseases related to fertilizer loss are suffered by a sample individual. Although the CLHLS database investigated 16 diseases in the elderly, including hypertension, heart disease, cancer, stroke, cardiovascular and cerebrovascular diseases, bronchitis, emphysema, asthma, and pneumonia, only three diseases—hypertension, cancer, and gastrointestinal ulcer—were related to fertilizer loss in the existing medical literature.

The age of onset refers to the age at which a sample individual first suffered from any of the three diseases (hypertension, gastrointestinal ulcer, or cancer).

2.1.2 Individual and household variables Individual and household variables include information on socio-economic characteristics, health behavior and dietary pattern. Socio-economic characteristics include age, gender, people living together, income station, basic health condition and hospitalization. Health behaviors measure whether the respondents smoke, drink or do any exercise (with options of "Yes" or "No"). Dietary patterns measure the frequency with which respondents eat meat, fish, egg, and salt-preserved vegetables. Options for each item are "rarely or never", "not every month, but occasionally", "not every week, but at least once per month", "not every day, but at least once per week", "almost every day", with scores from 1 to 5, respectively. The questionnaire also asks the sources of

respondents' drinking water. Options include drinking water sources from a well, a river of lake, a spring, a pond or pool, and tap water. A binary variable (Water) is constructed, which equals one if respondents drink surface water, i.e. water from a rive or lake or a pond of pool, and equals zero otherwise.

2.1.3 Provincial variables

Provincial variables include fertilizer loss, fertilizer use, organic fertilizer use, pesticide use, industrial pollution index, the numbers of hospital and provincial Gross Domestic Product. The calculation method for fertilizer loss intensity is based on literature [35]. On the basis of the unit investigation method and the inventory analysis method, the fertilizer loss coefficient method is used to calculate the total nitrogen and phosphorus emissions; then the total emission and emission intensity of fertilizer nonpoint-source pollution are calculated. The calculation formula is as follows:

$$E = \sum E_{ij} = \sum C_{ij} \times h_{ij} \quad (1)$$

$$I = E/A \quad (2)$$

where E measures the total emission of fertilizer loss; E_{ij} measures the amount of the pollutant j produced and the loss into water by pollutant i; C_i measures the index statistics of pollutant i (compound fertilizer is converted into total nitrogen 40% and phosphorus pentoxide 32%); η_{ij} measures the loss coefficient; A represents the crop areas; and I represents pollution emission per unit area, i.e., pollution intensity (unit: kg/ha).

The industrial pollution index ($Indpollution_i$) controls industrial activities, which defined in Equation (3) according to existing literature [36-37]. GDP measures general economy and wealth conditions. The number of hospitals controls the level of medical service. Because the health data are observed once three years, all provincial variables are assigned the average of the last three years.

$$Indpollution_i = \frac{pv_{i1} + pv_{i2} + pv_{i3}}{3}, \quad pv_{ij} = p_{ij} \Big/ \sum_{i=1}^{n} \frac{p_{ij}}{n} \quad (3)$$

where p_{ij} measures the pollutant j of province i, n is the amount of province, pv_{ij} measures the emission index of pollutant j in the province i relative to the national average level. The larger value means the

higher pollution level. $pv_{i1}, pv_{i2}, pv_{i3}$ represents industrial wastewater, industrial sulfur dioxide and industrial dust, respectively.

2.2 Statistical analyses

2.2.1 Sample summary

Table 1. Variable description and summary statistics

Variables	Description	Obs.	Mean	Std.Dev.	Min	Max
**Household Variables**						
Age	Age in years	39093	85.83	10.83	65	109
Male	Male=1;Female=0	39093	0.44	0.50	0	1
Co-residence	Nursing home=1; Alone or Spouse=2; Child=3; Others=4	39093	2.62	0.57	1	4
Income_cost	If income support daily cost (Yes=1; No=0)	39093	0.78	0.41	0	1
Illness	Number of 15 chronic diseases	39093	0.85	1.06	0	1
Hospitalization	The times of hospitalization in two years	39093	0.26	0.79	0	30
**Health Behaviors**						
Smoke	Currently smoke (Yes=1; No=0)	39093	0.33	0.47	0	1
Drink	Currently drink (Yes=1; No=0)	39093	0.31	0.46	0	1
Exercise	Currently exercise (Yes=1; No=0)	39093	0.27	0.44	0	1
Dietary Pattern						
Meat	The frequency of meat consumption	39093	3.95	1.03	1	5
Fish	The frequency of fish consumption	39093	3.36	1.15	1	5
Egg	The frequency of egg consumption	39093	3.92	1.07	1	5
Salt_vege	The frequency of salt-preserved vegetable consumption	39093	3.27	1.38	1	5
Boiled water	If drink boiled water (boild water=0; not boild water=1)	39093	0.057	0.23	0	1
Water	Drirking water source (Surface water=1; Tap water=0)	39093	0.14	0.35	0	1
**Provincial Variables**						
Floss	Fertilizer loss intensity (kg/ha/year)	39093	7.34	3.76	0.60	14.82
Finput	Fertilizer input intensity (kg/ha/year)	39093	354.80	92.47	128.41	704.32
Pinput	Pesticide inptut intensity (kg/ha/year)	39093	12.62	6.56	2.28	22.26
OFinput	Organic fertilizer input intensity (kg/ha/year)	39093	286.53	361.18	11.56	1997.00
Hospnum	The number of hospital per province (million)	39093	0.02	0.02	0.01	0.08
LnGDP	Logarithm of Provincial Gross Domestic Product	39093	9.10	0.91	7.32	11.04
Indpolltion	Index of industrial pollution (lower=better)	39093	1.46	0.55	0.45	2.95
**Health indicatos**						
ADL	Activities of Darly Living score (lower=better)	39093	6.66	1.80	6	18
No. of diseases	The number of illness caused by fertilizer loss (lower=better)	39093	0.25	0.48	0	3
Age_ill	Age of suffering from illness caused by fertilzer loss (lower=worse)	9503	83.70	10.06	65	109
Mescost	Medical cost(RMB)	39093	2768.29	11676.91	0	199996

2.2.2 Fertilizer loss and health outcome after agricultural support policies

China shifted from taxing agriculture to subsidizing agricultural programs since 2004 to maintain food security and self-reliance. In 2004, authorities introduced three subsides targeted at grain producers: a direct payment for grain producers, a subsidy for improved seed varieties and a partial rebate for farm machinery purchases. Several other support programs were introduced such as a general-input subsidy, price floors for wheat and rice, reform of the grain marketing system and transfer payment to grain counties since 2004, which has been accompanied with greater fertilizer use and fertilizer loss. This set of supporting programs had bolstered farmer' production incentives and crop production increased continuously since 2004 [38-39], on the other hand, the programs might lead to the distortion of agricultural factors and cause the negative effect of soil and water environment [40]. Table 2 reports the differences of health outcomes and fertilizer loss between regions with high versus low intensity of fertilizer loss. We report the differences of ADL index, the numbers of induces diseases, intensity of fertilizer input, amount of fertilizer loss and intensity of fertilizer loss before the implementation of agricultural support policies in column (3). The differences after the agricultural support policies are in column (6). The widening gap in health outcomes and fertilizer loss and input can be observed in column (7) between regions with high and low fertilizer loss after the agricultural support policies. Preliminary data analysis shows that the loss of chemical fertilizer in high-loss areas was more serious after the agricultural subsidy policies.

Table 2. Comparison of high-loss-areas and low-loss areas by agricultural support policies

Variables	Before agricultural Support Policies			After Agricultural Support Policies			2nd difference (7)=(6)-(3)
	Low loss (1)	High loss (2)	1st difference (3)=(2)-(1)	Low loss (4)	High loss (5)	1st difference (6)=(5)-(4)	
ADL index	6.823	7.022	0.199	6.822	7.108	0.287	0.088
No. of diseases	0.160	0.211	0.051	0.255	0.340	0.085	0.035
Input intensity (kg/ha)	239.562	358.413	118.851	317.519	457.163	139.644	20.793
Total loss (10000 tons)	1.689	6.717	5.028	2.023	7.350	5.327	0.299
Loss intensity (kg/ha)	2.905	8.599	5.694	3.491	9.226	5.735	0.041

2.2.3 Age of onset and fertilizer loss

The CLHLS contains three diseases (hypertension, gastrointestinal ulcer and cancer) induced by fertilizer loss. The age, when one sample first suffers from any of the three diseases, is defined age of onset The age

of onset comparisons across age groups represented by Fig 2. A significant difference of age of onset can be observed between the regions with high versus low intensity of fertilizer loss in younger-old groups (< 80 years) in Panel A of Fig 3. The age of onset is not signific[illegible] different in the oldest-old samples.

Panel A. Younger-old Samples   Panel B. Oldest-old Samples

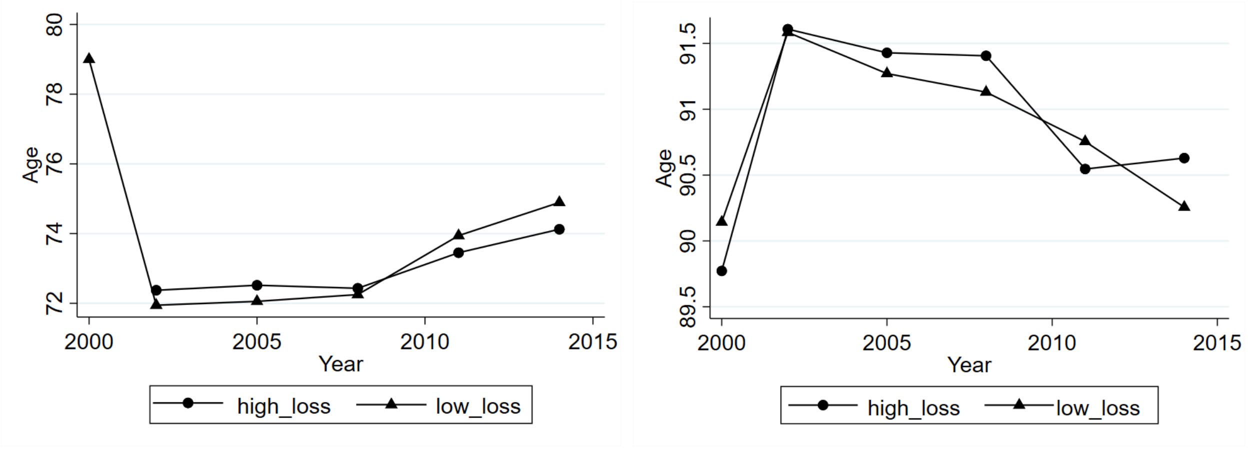

Fig 2. Chemical Fertilizer Loss and Age of Onset.

3. Empirical models and results

This section investigates the health effect of fertilizer loss on local elderly population. Preliminary data analysis reports the health the differences in regions with high verse low intensity of fertilizer loss after agricultural subside policies in 2004. Further, we follow a difference-in-differences (DID) framework to compare health outcomes between people in regions with different intensities of fertilizer loss before and after 2004, when China shifted from taxing agricultural outputs to subsidizing agriculture. The medical cost are estimated as well. To facilitate comparisons across age groups, explore heterogeneous effects by splitting the sample in various age groups and reporting estimates of α_1 and β_1 for these subsamples. Then several robustness checks are conducted to help assess the validity of our results. The difference-in-differences model is specified as:

$$Health_{it} = \alpha_0 + \alpha_1 Fertilizerloss_{it} \times Time_{it} + \alpha_2 Fertilizerloss_{it} + \alpha_3 Time_{it} + \alpha_4 X_{it} + \gamma_t + \mu_i + \varepsilon_{it} \quad (4)$$

$$Health_{it} = \beta_0 + \beta_1 Area_{it} \times Time_{it} + \beta_2 Area_{it} + \beta_3 Time_{it} + \beta_4 X_{it} + \gamma_t + \mu_i + \varepsilon_{it} \quad (5)$$

$$Age_{it} = \delta_0 + \delta_1 Fertilizerloss_{it} \times Time_{it} + \delta_2 Fertilizerloss_{it} + \delta_3 Time_{it} + \delta_4 X_{it} + \gamma_t + \mu_i + \varepsilon_{it} \quad (6)$$

$$Age_{it} = \theta_0 + \theta_1 Area_{it} \times Time_{it} + \theta_2 Area_{it} + \theta_3 Time_{it} + \theta_4 X_{it} + \gamma_t + \mu_i + \varepsilon_{it} \quad (7)$$

where $Health_{it}$ denotes the health outcome; Age_{it} denotes the age of onset; α_1, β_1, δ_1 and θ_1 identify the effects of fertilizer loss; $Fertilizerloss_{it}$ denotes the fertilizer loss of the region where individuals i resides; $Area_{it}$ is a dummy variable equaling 1 if the individual is in high loss areas, and 0 otherwise; $Time_{it}$ equals 1 starting in 2004 and 0 otherwise; X_{it} is a vector of control variables including individual, household, and provincial characteristics such as age, gender, health behavior, dietary habits, industrial pollution and hospitals, etc.; μ_i is a fixed effect unique to individuals i; γ_t is a time effect common to all individuals in year t; and ε_{it} is an error term.

The relationship between medical cost and health can be identified in the following Equation:

$$Y_{it} = \alpha_0 + \alpha_1 ADL_{it} + \alpha_2 X_{it} + \lambda_t + \mu_i + \varepsilon_{it} \tag{8}$$

where Y_{it} represents medical costs, and other variables are as defined as before.

3.1 Basic regression results of health

3.1.1 Effect of fertilizer loss on health

Table 3 reports the α_1 and β_1 coefficients in Equation (4) and Equation (5) for outcomes related to ADL index and No. of diseases. Estimated coefficients in column (1) of Table3 indicate that one kg/ha increase of chemical fertilizer loss is associated with increased ADL daily life index of 0.0147. From column (4) one kg/ha increase of fertilizer loss is associated with 0.0057 raise in the number of diseases. These results indicate that the fertilizer loss increase the elderly's health risks and support diseases significantly. For different regions, estimates for β_1 in columns (2) and column (5) of Table 3 indicate a reduction in the ADL index and the number of diseases for high-loss area verse low-loss areas. Compared with low-loss areas, the ADL index of the rural elderly is 0.1222 higher in areas with high chemical fertilizer loss, while the number of diseases is 0.0487 higher. We also use the two-way fixed effects model to estimate the health effect of fertilizer loss. Column (3) and Column (6) of Table 3 report the coefficients for outcomes related to ADL index and No. of diseases. Estimated coefficients indicate that one kg/ha of fertilizer loss increase is associated with increased ADL daily life index of 0.1088. From column (6) one kg/ha of fertilizer loss increase is associated with 0.0211

raise in the number of diseases. The health effect of agricultural fertilizer input not only accrue in farmers who are likely to receive a fertilizer loss via direct exposure. Many more proportions of the population are at risk of fertilizer loss because of exposure to contaminated water and soil. Agricultural support policies play an important role in the fertilizer loss, and the agricultural may cause heterogeneous fertilizer loss between regions because of the different subsides levels. These may cause the biased result of TWFE, DID model can avoid this problem, and we control the time effect and individual effect in our regressions.

Table 3. Basic Estimates ADL index and Diseases

	ADL			No. of diseases		
	DID		TWFE	DID		TWFE
	(1)	(2)	(3)	(4)	(5)	(6)
Fertilizerloss*Time	0.0147**	--	--	0.0057***	--	--
	(0.0060)	--	--	(0.0014)	--	--
Higharea*Time	--	0.1222***	--	--	0.0487***	--
	--	(0.0461)	--	--	(0.0114)	--
	--	--	0.1088**	--	--	0.0211*
	--	--	(0.0432)	--	--	(0.0126)
Control	YES	YES	YES	YES	YES	YES
Year FE	YES	YES	YES	YES	YES	YES
ID FE	YES	YES	YES	YES	YES	YES
Observations	32165	32165	38908	32329	32329	38680
R-squared	0.5570	0.5582	0.0843	0.6906	0.6907	0.3243

[a] robust standard errors for clustering at the individual level are reported in brackets; *, **, *** are significant at the level of 10%, 5% and 1% respectively.

3.1.2 Heterogeneous health effects

In S1 and S2 Tables, we explore the heterogeneous effects by splitting the samples in different age groups and reporting estimates of α_1 for these subsamples. First, the oldest olds (≥80years) were discussed in previous studies [41], because the oldest olds had the greater risk of falling and hospitalization [42-44]. We divide the samples at age into the younger age samples (<80years) and the oldest olds samples (≥80 years). In Columns (1) and columns (2) of S1 Table, a positive and significantly estimate of α_1 can be observed in the oldest olds. The estimates of α_1 are positive and significantly for both age groups (columns (3) and columns (4) of S1 Table), the fertilizer loss will increase the risk of diseases in both the younger olds and the oldest olds. The grouped estimates for effect of fertilizer loss on ADL and diseases of the oldest old are

significantly distinguishable from the younger olds at the 1% level. We also divide the samples into younger olds (65years-75years), middle olds (75-85years) and the oldest olds (≥85years) [45-47]. The differences between younger and middle olds are not significant in both ADL and No. of diseases. The coefficient for oldest olds is larger than that for younger and middle olds, and the differences are significant.

3.1.3 Effect of fertilizer loss on Age of Onset

Table 4 reports the δ_1 and θ_1 coefficients in Equation (6) and Equation (7) for outcomes related to age of onset. Fertilizer loss increases the risk of disease in the rural elderly. This study further explores whether fertilizer loss will lead to the elderly be sick in younger age. Though from Fig 2, the age of onset of elderly in areas with high-loss of chemical fertilizer are younger, in order to understand the relationship between fertilizer loss and the age of onset, we use the difference-in-differences model for empirical analysis. Estimated coefficient in column (1) of Table 4 indicates that one kg/ha increase of fertilizer loss advances the age by 0.2670 year. Comparing with low-loss areas, the age of onset is declines 0.3753 in high-loss areas.

Table 4. Estimation of fertilizer loss on age of onset

	Age of Onset	
	(1)	(2)
Fertilizerloss*Time	-0.2670*<br>(0. 0161)	
Higharea*Time		- 0.3753**<br>(0. 1258)
Control Variables	YES	YES
Year FE	YES	YES
Individual FE	YES	YES
Observations	9503	9503
R-squared	0.9822	0.9822

[a] *, **, *** are significant at the level of 10%, 5% and 1% respectively.

3.2 Results of health conditions on medical cost

In this section, we further explore these results by translating estimated effects into expected monetary losses. We do this by exploiting the relation between ADL and medical cost in Equation (8). Estimated coefficient in column (1) of Table 5 indicates that each additional unit increase in ADL index increases the medical cost by CNY 244.6 (USD 34.94). Columns (2) and (3) report the λ_1 coefficient in Equation (8) in high-loss and low-loss areas, respectively. The estimates indicate that there is a significant difference in medical cost between high loss areas and low loss areas, every one-unit ADL increase is associated with the

medical cost CNY 194.64 (USD 27.81) higher in high loss areas. The health cost per unit loss of chemical fertilizer (1kg/ha) is about CNY 3.64 (USD 0.51). According to the sixth census, there were about 87.91 million rural elderly people (over 65 years of age) in China, and the loss of chemical fertilizer per unit will add medical costs by CNY 316 (USD 45) million. Though agro-chemical use is associated with significantly greater agricultural output value a range studies showed that agro-chemical also damaged human health. Indirectly, medical cost increases and labor supply loss could be observed due to illness [48]. Similar to our study, Lai suggested that a 10%(CNY 0.2/USD 0.03) increase in rice pesticide use will add 168.8 and 55.89 million dollars to medical costs and offspring's human capital losses, respectively [6]. Compared with the pesticide use, we estimate the economic loss of fertilizer loss, an increase of one1kg/ha of fertilizer loss and fertilizer input will add CNY 316 (USD 45) million and CNY 21.5 (USD 3.1) million to national medical cost, respectively.

Table 5. Effect of health on medical cost

	Medical Cost		
	Overall samples	High-loss area	Low-loss area
ADL index	244.60***	331.94***	137.30**
	(55.81)	(93.54)	(63.58)
Control Variables	YES	YES	YES
Year FE	YES	YES	YES
Individual FE	YES	YES	YES
Observations	23087	19875	15167
R-squared	0.0709	0.0226	0.0121
p			0.0853*

[a]*, **, *** are significant at the level of 10%, 5% and 1% respectively.

3.3 Robustness check

3.3.1 Parallel trend test

In order to test that the time staggered entry events of agricultural policy are effective exogenous shocks, this study makes a parallel test. We divide the samples to five sections: the first period before the policy implementation, the second period after policy, the second two periods after the policy implementation to the end of sample period. Thus, five indicator variables pre_1, post_1, post_2, post_3 and post_4 are constructed. S1 Fig, Panel (1) plots estimates of ADL index and Panel plots estimates of No. of diseases. The set of pre_1 coefficient is statistically indistinguishable from zero, but there is a sharp, statistically significant and sustained change after the policy implementation.

3.3.2 Placebo test

In order to further test whether the results are driven by unobservable factors of the individual and provincial level, referring to the methods of existing literature [49-50], this study conducted a placebo test by randomly assigning high and low chemical fertilizer loss areas. Specifically, 9 out of 19 provinces are randomly selected as the experimental group, assuming that these 9 provinces are areas with high loss of chemical fertilizer, and other regions are the control group (areas with low loss of chemical fertilizer). Random sampling ensures that the model constructed in this paper has no effect on the health level of the elderly. In this study, random sampling is carried out 1000 times, and the benchmark regression is performed according to Equation (5). S2 Fig reports the average value of the ADL index and the number of induced diseases after 1000 random distributions. The mean value of1000 random sample regressions is almost 0. The placebo test shows that our basic estimation results are unlikely to be driven by unobservable factors at the individual and provincial level.

3.3.3 Effect of fertilizer input on health

In order to verify the robustness of the research conclusions, the application intensity of chemical fertilizer and the areas with high and low chemical fertilizer application intensities are used to test the robustness. According to the construction index of ecological counties in China (the application intensity of chemical fertilizer should not exceed 250 kg/ha), the areas with a chemical fertilizer application intensity exceeding 250 kg/ha are defined as high input areas, and the areas with a chemical fertilizer application intensity not exceeding 250 kg/ha are defined as low input areas. The basic regression of Equations (4) and (5) are carried out using the chemical fertilizer application intensity. The model results are shown in S3 Table. The model results are essentially consistent with the benchmark regression results, indicating that the research results are robust. For every 1 kg/ha increase in fertilizer input intensity, the ADL index will increase by 0.001 and the number of induced diseases will increase by 0.0001

3.3.4 Effect in different areas

In order to verify the robustness of the conclusions of this study, different samples are used to test the robustness. This paper focuses on health effect of agricultural fertilizer loss of the rural elderly. Therefore, samples from major agricultural-producing areas, major rice-producing areas and water resource-rich areas are used to carry out the basic regression of Equation (4) and Equation (5). The results (S5 and S6 Tables) show that fertilizer loss lead a significant increase both on ADL index and the number of diseases in major agricultural-producing areas, major rice-producing areas and water resource-rich areas, which are basically same comparing with the basic regression results in table3. The basic regression results are robust to a variety of robustness checks.

4. Conclusions and discussion

China's agricultural fertilizer input is facing a problem of "high input, high loss, and high pollution", which had been aggravated by agricultural support policies since 2004. The problem of the "Three Highs" of chemical fertilizer use has caused pollution to the environment and health damage, many more rural residents may be at health risk because of fertilizer loss. In this paper, the chemical fertilizer loss data and CLHLS micro-health data are used to verify the health effect of fertilizer loss of the rural elderly. China had the most serious aging problem; it was estimated that the number of the elders might increase to 450 million (nearly one third of the total population) by 2050. Nearly 60% elders live in rural areas [51]. The health decline caused by fertilizer loss not only increased the medical cost but also affected the economics, e.g. reducing labor time, increasing family care time and so on [52-53]. The health effect of fertilizer maybe a greater challenge to pension and health care in rural China. To improve the health station of elderly in rural areas, the government can consider from agricultural policy and public health policy. (1) reduce fertilizer loss by innovating agricultural production technology research and promoting technology adoptions. e.g. soil testing, formula fertilization technology, slow-release fertilizer technology, deep tillage machinery promotion, and straw returning technology. (2) as the loss of chemical fertilizer includes not only the loss from the current year, but also the basic loss of fertilizer, the government should improve soil quality to increase nutrient preserving capability of soil,, such as soil improvement technology, etc. (3) drinking water is an important way of

fertilizer loss affect the public health, so the government need to focus on the rural drinking water, improve the rural elderly's awareness of drinking safety and the health damages of chemical fertilizer loss.

The health effects of agricultural nonpoint-source pollution are estimated by taking the fertilizer as an example. Due to the limitations of the article, this article does not discuss the health effect of other agricultural pollution, such as pesticides use, straw combustion and the interaction of various agricultural pollutions. The nonpoint-source pollution in animal husbandry and fishery is also worth to discuss.

References

1. NBSC. 2016. China Statistical Yearbook. Beijing, National Bureau of Statistics of China.
2. Zhou K, Fan J, Liu HC. 2017. Spatiotemporal patterns and driving forces of water pollutant discharge in the Bohai Rim region. Progress in Geography. 2020; 36(02): 171-181.
3. Sun XY, Wang J, Jin YT. Advances in research on pesticide pollution to the aquatic environment and health impact in China. Journal of Environment and Health. 2009;26(07): 649-652.
4. Brainerd E, Menon N. Seasonal effects of water quality: The hidden costs of the green revolution to infant and child health in India. Journal of Development Economics. 2014; 107: 49-64. doi: 10.1016/j.jdeveco.2013.11.004.
5. Lai WY. Pesticide use and health outcomes: evidence from agricultural water pollution in China. Journal of Environmental Economics and Management. 2017; 86: 93-120. doi:10.2139/ssrn.2751458.
6. He GJ, Liu T, Zhou MG. Straw burning, PM 2.5, and death: Evidence from China. Journal of Development Economics. 2020;145: 102468. doi: 10.1016/j.jdeveco.2020.102468.
7. Wong CM, Lai HK, Tsang H, Thach TQ, Thomas GN, et al. Satellite-based estimates of long-term exposure to fine particles and association with mortality in elderly Hong Kong residents. Environmental Health Perspectives. 2015;123(11):1167–1172. doi: 10.1289/ehp.1408264. Epub 2015 Apr 24. PMID: 25910279.
8. Sacks JD, Stanek LW, Luben TJ, Johns DO, Buckley BJ, et al. Particulate matter-induced health effects: who is susceptible? Environmental Health Perspectives. 2011;119(4):446-54. doi:10.1289/ehp.1002255. PMID: 20961824.
9. Cohen, G., Gerber, Y. Air pollution and successful aging: Recent evidence and new perspectives. Current Environmental Health Reports. 2017; 4:1-11. https://doi.org/10.1007/s40572-017-0127-2. PMID: 28101729.
10. Assembly of First Nations (AFN). Environmental health older adults and seniors (elders). Ottawa, Canada. 2009.
11. Tuttle L, Meng Q, Moya J, Johns DO. Consideration of age-related changes in behavior trends in older adults in assessing risks of environmental exposures. Journal of Aging and Health. 2013; 25(2):243-273. doi:10.1177/0898264312468032. PMID: 23223208
12. Health Canada. Health of older adults and the environment discussion paper. Ottawa, Canada. 2008.
13. Tong YF. Changes and challenges of labor supply in China in the context of population ageing. Population Research. 2014; 38(02): 52-60.
14. Li JX, Li CH. Health difference of the elderly between the rural and urban districts. Population Journal. 2014; 36(05):37-47.

15. Weeks WB, Kazis LE, Shen TJ, Cong ZX, Ren XH, et al. Differences in health-related quality of life in rural and urban veterans. American Journal of Public Health. 2004; 10:1762-1767. https://doi.org/10.2105/AJPH.94.10.1762.
16. Khanal SJ, Pokhrel RP, Pokharel B, Becker S, Giri B, et al. An episode of transboundary air pollution in the central Himalayas during agricultural residue burning season in North India. Atmospheric Pollution Research. 2022; 13(1): 101270. https://doi.org/10.1016/j.apr.2021.101270.
17. Huang L, Zhu YH, Wang Q, Zhu AS, Liu ZY, et al. Assessment of the effects of straw burning bans in China: Emissions, air quality, and health impacts. Science of The Total Environment. 2021; 789: 147935. https://doi.org/10.1016/j.scitotenv.2021.147935.
18. Nganchamung T, Robson M. Chemical fertilizer use and acute health effects among Chili farmers in Ubon Ratchathani Province, Thailand. Journal of Health Research.2017; 31(6): 427-435. doi:10.14456/jhr.2017.53
19. Gorman NM, Stjernberg E, Koehoorn M, Demers PA, Winters M, et al. Fertilizer use and self-reported respiratory and dermal symptoms among tree planters. Journal of Occupational and Environmental Hygiene. 2013; 10(1): 36-45. doi: 10.1080/ 15459624.2012.740994.
20. Qiao F, Huang J, Zhang L, Rozelle S. Pesticide use and farmers' health in China's rice production. China Agricultural Economic Review. 2012; 4: 468-484. https://doi.org/10.1108/17561371211284821.
21. Zhang WD, Sohngen B. Do U.S. anglers care about harmful algal blooms? A discrete choice experiment of Lake Erie recreational anglers. American Journal of Agricultural Economics, Agricultural and Applied Economics Association.2018; 100(3): 868-888. https://doi.org/10.1111/ajae.12130
22. Paudel J, Crago CL. Environmental externalities from agriculture: Evidence from water quality in the United States. American Journal of Agricultural Economics. 2021; 103: 185-210. https://doi.org/10.1111/ajae.12130
23. China pollution source census. Technical report on China pollution source census. Beijing: China Environmental Press, 2011.
24. National Bureau of Statistics. China statistical yearbook (2019). Beijing: China Statistics Pres, 2020.
25. Zahra, Bahadoran, Asghar. Nitrate-nitrite-nitrosamines exposure and the risk of type 1 diabetes: A review of current data. World Journal of Diabetes. 2016; 7:433. doi: 10.4239/wjd.v7.i18.433. PMID: 27795817
26. Fan AM, Steinberg VE. Health implications of nitrate and nitrite in drinking water: An update on methemoglobinemia occurrence and reproductive and developmental toxicity. Regulatory Toxicology and Pharmacology.1996; 23:35-43. doi:10.1006/rtph.1996.0006.
27. Kristensen P, Andersen A, Irgens LM. Testicular cancer and parental use of fertilizers in agriculture. Cancer epidemiology, biomarkers & prevention. 1996; 5:3-9. PMID: 8770459
28. Ward MH. Too much of a good thing? Nitrate from nitrogen fertilizers and cancer. Reviews on Environmental Health. 2009; 24, 357-363. doi:10.1515/REVEH.2009.24.4.357.
29. Forman D. Are nitrates a significant risk factor in human cancer? Cancer Surveys.1989; 8(2): 443-458. PMID: 2696589
30. Bivolarska A, Gatseva P. Thyroid status in pregnant women and association with nitrates as an environmental factor stimulating the manifestation of iodine deficiency. Trace Elements and Electrolyte. 2015; 32:60-64. doi: 10.5414/tex01365.
31. Manasaram DM, Backe LC, Moll DM. A review of nitrates in drinking water: Maternal exposure and adverse reproductive and developmental outcomes. Environmental Health Perspectives. 2006; 114: 320-327. doi:10.2307/3436672.
32. Uddin M.S, Kurosawa K. Effect of chemical nitrogen fertilizer application on the release of arsenic from sediment to groundwater in Bangladesh. Procedia Environmental Sciences. 2011; 4: 294-302. doi: 10.1016/j.proenv.2011.03.034.
33. Smith AH, Hopenhayn-Rich C, Bates MN. Cancer risks from arsenic in drinking water. Environmental Health Perspectives. 1992; 97:259. doi: 10.1289/ehp.9297259.

34. Johnson RJ, Wolinsky FD. The structure of health status among older adults: disease, disability, functional limitation, and perceived health. Journal of Health Social Behavior. 1993; 34: 105-121. doi:10.2307/2137238
35. Lei J, Su SP, Yu WM. Temporal and spatial pattern evolution and grouping prediction of non-point source pollution of chemical fertilizers in China. Chinese Journal of Eco-Agriculture. 2020; 28(07): 1079-1092. doi: 10.13930/j.cnki.cjea.190923.
36. Shen KR, Jin G, Fang X. Dose environmental regulation cause pollution to transfer nearby? Economic Research Journal. 2017; 52(05): 44-59. http://www.cesgw.cn/cn/mlInfo.aspx?m=20170217100514860651&n=20170609120846613735&tip=4.
37. Zhu FP, Zhang ZY, Jiang GL. Empirical study of the relationship between FDI and environmental regulation: An intergovernmental competition perspective. Economic Research Journal. 2011; 46(06): 133-145. http://www.cesgw.cn/cn/mlInfo.aspx?m=20110125121729123417&n=20110617154203400510&tip=10
38. Chen HP, Wu LP, Wang YB. Impacts of subsidy policy on grain production: An empirical analysis based on sub-provincial data of grain production from 2004—2007. Journal of Agro technical Economics. 2010; 4: 100-106.
39. Chen F, Fan QQ, Gao TM. Agricultural policies, food production and food production-adjustment ability. Economic Research Journal. 2010; 45(11): 101-114+140.
40. Ge JH, Zhou SD. An analysis of the economic determinants to agricultural non-point source pollution: Based on data of Jiangsu province during 1978-2009. Chinese Rural Economy, 2011; (5): 72-81.
41. Callaway B, Goodman-Bacon A, and Pedro H.C. Sant'Anna. Difference-in-differences with a continuous treatment. Working Paper, 2021.
42. Brettschneider C, Hajek A, Rohr S, Fuchs A, Weeg D, et al. Determinants of health-care costs in the oldest-old in Germany. The Journal of the Economics of Ageing. 2019; 14: 100200. https://doi.org/10.1016/j.jeoa.2019.100200.
43. Winograd CH. Targeting Strategies: An overview of criteria and outcomes. Journal of the American Geriatrics Society. 1991; 39:25-35. https://doi.org/10.1111/j.1532-5415.1991.tb05930.x
44. Speechley M, Tinetti M. Falls and injuries in frail and vigorous community elderly persons. Journal of the American Geriatrics Society. 1991; 39:46-52. doi: 10.1111/j.1532-5415. 1991.tb05905. x. PMID: 1987256.
45. Rockwood K, Stadnyk K, MacKnight C, McDowell I. A brief clinical instrument to classify frailty in elderly people. The Lancet. 1999; 353:205-206. doi: 10.1016/S0140-6736(98)04402-X. PMID: 9923878.
46. Lau LK, Wee SL, Mallya JU, Yap, Pang, et al. Physiological and cognitive determinants of gait in middle-aged, older-aged and oldest-aged Asian adults – The Yishun study. Aging and Health Research. 2021; 1(3): 100030. https://doi.org/10.1016/j.ahr.2021.100030.
47. Monti A, Doulazmi M, Nguyen-Michel VH, Pautas E, Mariani J, et al. Clinical characteristics of sleep apnea in middle-old and oldest-old inpatients: symptoms and comorbidities. Sleep Medicine. 2021; 82: 179-185. https://doi.org/10.1016/j.sleep.2021.04.017.
48. Sheahan M, Barrett CB, Goldvale, C. The unintended consequences of agricultural input intensifycation: human health implications of agro-chemical use in Sub-Saharan Africa. Working Paper Series. 2016; 234. https://www.afdb.org/en/documents/document/working-paper-234-the-unintended-consequences-of-agricultural-input-intensification-human-health-implications-of-agro-chemical-use-in-sub-saharan-africa-87634.
49. Cai XQ, Lu Y, Wu MQ. Does environmental regulation drive away inbound foreign direct investment? evidence from a quasi-natural experiment in China. Journal of Development Economics. 2016; 123: 73-85. doi: 10.1016/j.jdeveco.2016.08.003.
50. Ferrara LE, Chong A, Duryea S. Soap operas and fertility: evidence from Brazil. Applied Economics. 2012; 4: 1-31. doi:10.2139/ssrn.1820921.

51. Dai BZ. The old age health security in rural China: where to go? International Journal for Equity Health. 2015; 14: 119. doi: 10.1186/s12939-015-0224-5.
52. Mitra S, Gao Q, Chen W, Zhang YL. Health, work, and income among middle-aged and older adults: a panel analysis for China. The Journal of the Economics of Ageing. 2020; 17:100255. doi: 10.1016/j.jeoa.2020.100255.
53. Behncke, S. Does retirement trigger ill health? Health Economics. 2012;21: 282-300. doi:10.1002/hec.1712.

Supporting information

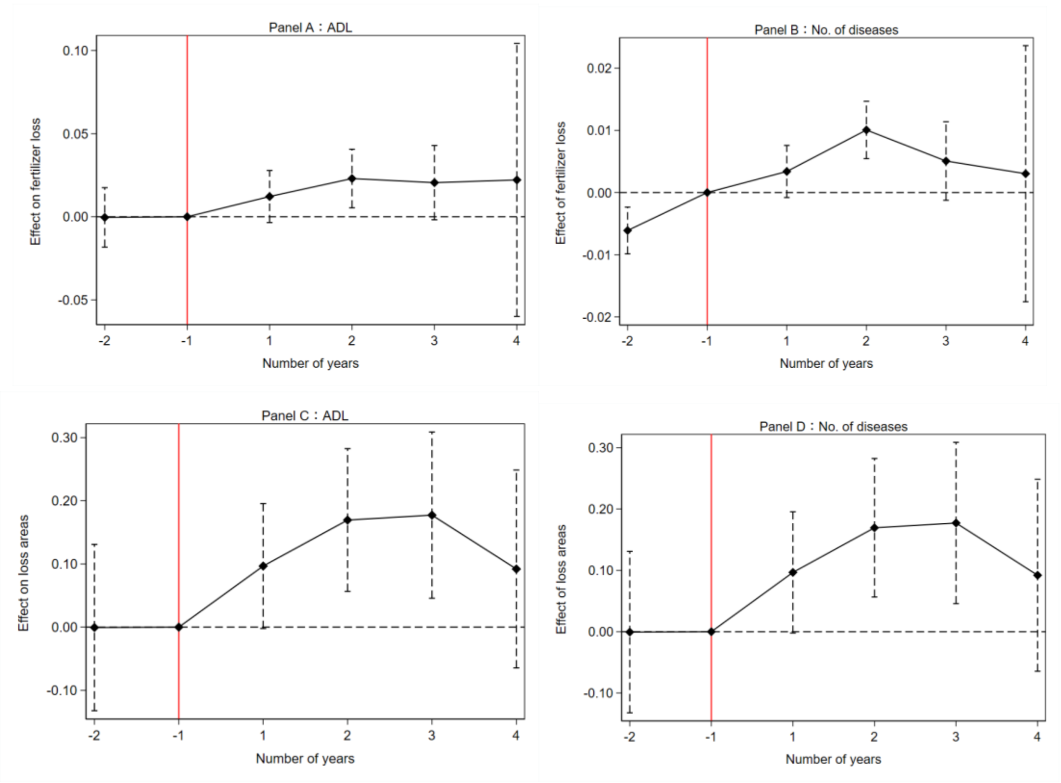

S1 Fig Parallel Trend Test

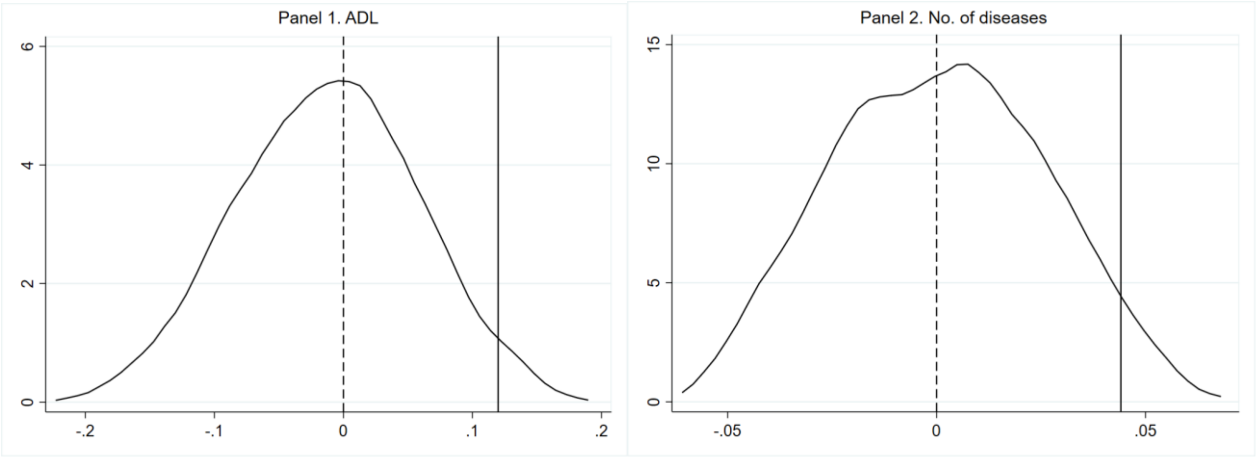

S2 Fig Placebo Test.

Panel 1 is the result of the ADL index placebo test; Panel 2 is the result of the placebo test of the number of patients. The X-axis represents the estimated coefficients from 1000 random assignments. The curve is the estimated kernel density distribution. The vertical type is a true estimate in columns (2) and (4) of Table 3.

S1 Table Basica regression results without control variables

	ADL		No. of diseases	
	(1)	(2)	(3)	(4)
Fertilizerloss*Time	0.0175***		0.0052***	
	(0.0057)		(0.0016)	
Higharea*Time		0.1608***		0.0441***
		(0.0432)		(0.0124)
Year FE	YES	YES	YES	YES
ID FE	YES	YES	YES	YES
Observations	32,302	32,302	32,467	32,467
R-squared	0.5474	0.5475	0.5597	0.5597

[a]*, **, *** are significant at the level of 10%, 5% and 1% respectively.

S2 Table Heterogeneous estimation of yonger olds and oldest olds

	(1)	(2)	(3)	(4)
	ADL		No. of diseases	
	Younger olds	Oldest olds	Younger olds	Oldest olds
Fertilizerloss*Time	-0.0006	0.0356***	0.0033**	0. 0086***
	(0.0038)	(0.0074)	(0.0014)	(0.0114)
Control	YES	YES	YES	YES
Year FE	YES	YES	YES	YES
ID FE	YES	YES	YES	YES
Observations	20536	22367	20620	22431
R-squared	0.0761	0.1359	0.3418	0.2951
p	0.0008***		0.0204**	

[a]*, **, *** are significant at the level of 10%, 5% and 1% respectively.

S3 Table Heterogeneous estimation of yonger olds, middle olds and oldest olds

	(1)	(2)	(3)	(4)	(5)	(6)
		ADL			No. of diseases	
	Younger olds	Middle olds	Oldest olds	Younger olds	Middle olds	Oldest olds
Fertilizerloss*Time	0.0027	0.0000	0.0334***	0. 0018	0.0039*	0. 0074***
	(0.0040)	(0.0061)	(0.0071)	(0. 0022)	(0. 0021)	(0.0114)
Control	YES	YES	YES	YES	YES	YES
Year FE	YES	YES	YES	YES	YES	YES
ID FE	YES	YES	YES	YES	YES	YES
Observations	8332	10719	23852	8360	10769	23922
R-squared	0.0548	0.0885	0.1287	0.3416	0.3398	0.2954
p	P^{12}=0.7163			P^{45}=0.4969		
	P^{23}=0.0039***			P^{56}=0.1936		
	P^{13}=0.0029***			P^{46}=0.0419**		

[a]*, **, *** are significant at the level of 10%, 5% and 1% respectively.

S4 Table Robustness check of fertilizer input and fertilizer input areas

	ADL		No. of diseases	
	(1)	(2)	(3)	(4)
Fertilizer input*Time	0.0010***		0.0001*	
	(0.0003)		(0.0001)	
Higharea*Time		0.2168***		0.0061
		(0.0547)		(0.0148)
Control	YES	YES	YES	YES
Year FE	YES	YES	YES	YES
ID FE	YES	YES	YES	YES
Observations	32443	32278	32443	32443
R-squared	0.6905	0.5646	0.6905	0.6903

[a]*, **, *** are significant at the level of 10%, 5% and 1% respectively.

S5 Table Additional robustness check of fertilizer loss in different areas

	Major Agri-producing areas		Major Rice-producing areas		Water resource rich areas	
	ADL index	No. of diseases	ADL index	No. of diseases	ADL index	No. of diseases
Fertilizerloss*Time	0.0283***	0.00200	0.0203***	0.00500***	0.0274	0.0161***
	(0.0082)	(0.0021)	(0.0066)	(0.0017)	(0.0175)	(0.0043)
Control Variables	YES	YES	YES	YES	YES	YES
Year FE	YES	YES	YES	YES	YES	YES
Individual FE	YES	YES	YES	YES	YES	YES
Observations	16880	16959	21528	21603	20526	20604
R-squared	0.572	0.695	0.549	0.677	0.533	0.667

[a]*, **, *** are significant at the level of 10%, 5% and 1% respectively.

S6 Table Additional robustness check of fertilizer loss areas in different areas in different areas

	Major Agri-producing areas		Major Rice-producing areas		Water resource rich areas	
	ADL index	No. of diseases	ADL index	No. of diseases	ADL index	No. of diseases
Higharea*Time	0.255***	0.0389**	0.163***	0.0452***	0.144**	0.0339**
	(0.0677)	(0.0171)	(0.0567)	(0.0141)	(0.0613)	(0.0158)
Control Variables	YES	YES	YES	YES	YES	YES
Year FE	YES	YES	YES	YES	YES	YES
Individual FE	YES	YES	YES	YES	YES	YES
Observations	16880	16959	21528	21603	20526	20604
R-squared	0.571	0.695	0.549	0.677	0.532	0.667

[a]*, **, *** are significant at the level of 10%, 5% and 1% respectively.

1. High loss areas include Anhui, Shandong, Jiangsu, Guangdong, Hebei, Henan, Zhejiang, Hubei and Fujian, while low loss areas include Chongqing, Sichuan, Shaanxi, Guangxi, Hunan, Jiangxi, Heilongjiang,

Jilin, Liaoning and Shanxi. In this paper, data from Beijing, Tianjin and Shanghai are deleted because of the small sample size and the small agricultural planting area.

2. Data source of loss coefficient: technical report of the first general survey of pollution sources.

3. The major agricultural-producing areas are Hebei Province, Inner Mongolia Autonomous Region, Heilongjiang Province, Jiangsu Province, Anhui Province, Shandong Province, Henan Province, Hubei Province, Hunan Province and Sichuan Province.

The major rice-producing areas are Heilongjiang Province, Jiangsu Province, Anhui Province, Jiangxi Province, Hubei Province, Hunan Province, Guangdong Province, Guangxi Zhuang Autonomous Region, Chongqing and Sichuan Province.

The water resource rich areas are Heilongjiang Province, Zhejiang Province, Anhui Province, Fujian Province, Jiangxi Province, Hubei Province, Hunan Province, Guangdong Province, Guangxi Zhuang Autonomous Region and Sichuan Province.

Figure Click here to access/download;Figure;Figures.doc

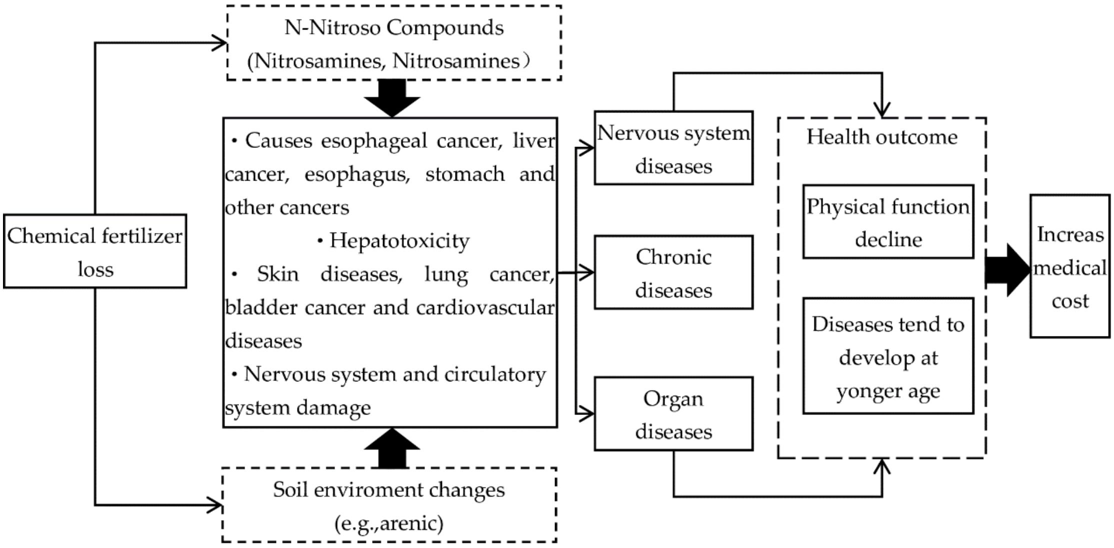

Fig1. Effects of Chemical Fertilizer Loss on Health.

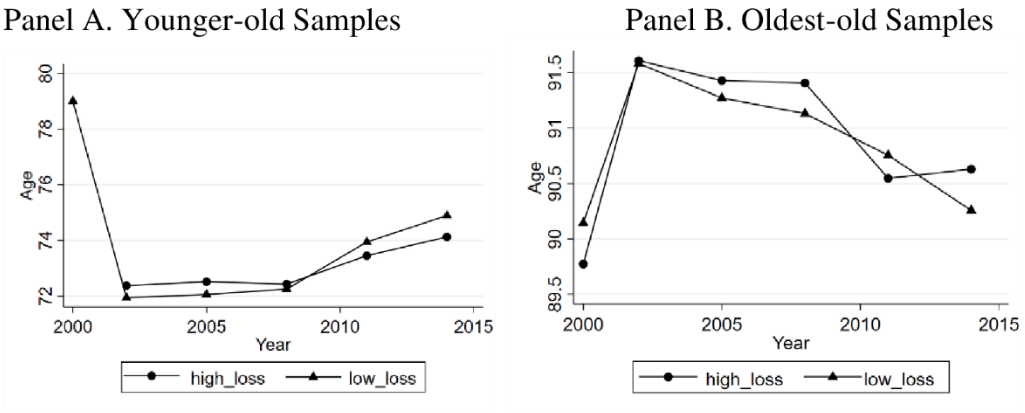

Fig 2. Chemical Fertilizer Loss and Age of Onset.

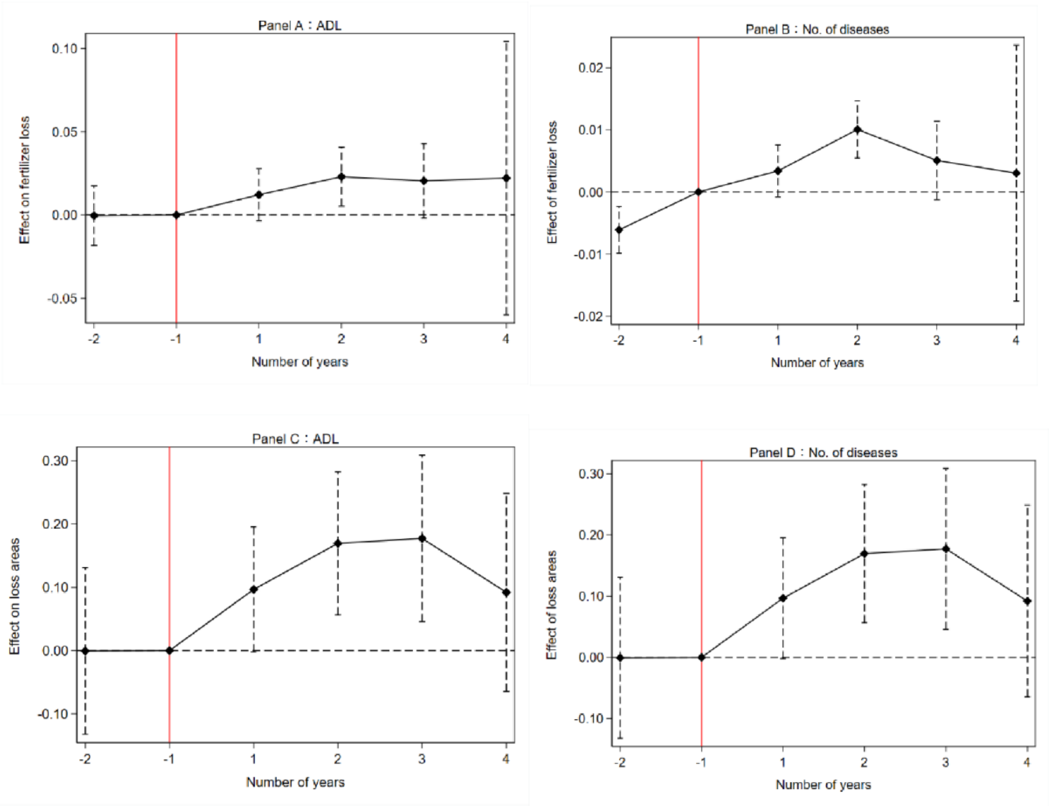

S1 Fig Parallel Trend Test

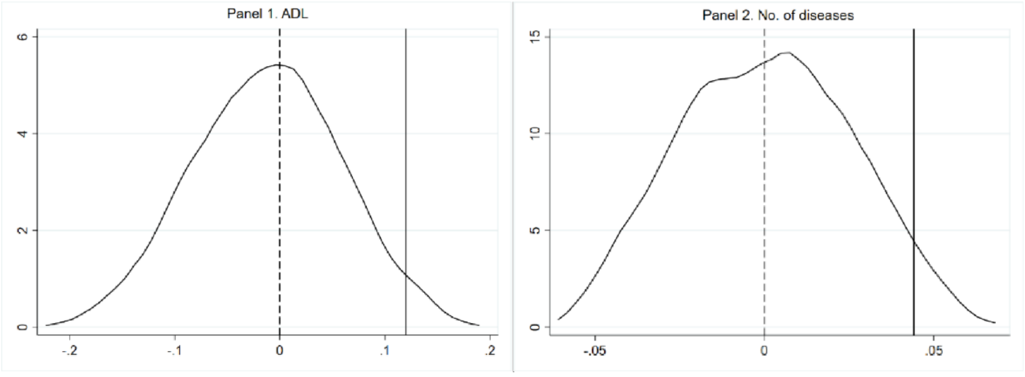

S2 Fig Placebo Test.

Panel 1 is the result of the ADL index placebo test; Panel 2 is the result of the placebo test of the number of patients. The X-axis represents the estimated coefficients from 1000 random assignments. The curve is the estimated kernel density distribution. The vertical type is a true estimate in columns (2) and (4) of Table 3.

Revised Manuscript with Track Changes

Agricultural Nonpoint-source Pollution and Health of the Elderly in Rural China

~~Health Effects of Non-point Pollution on the Elderly in Rural Areas of China~~

Ying WANG[1], Hang XIONG[2], Chao CHEN[3*]

[1] College of Economics and Management, Nanjing Agricultural University, Nanjing, Jiangsu, P.R.China

[2] College of Economics and Management, Nanjing Agricultural University, Nanjing, Jiangsu, P.R.China; China Center for Food Security Studies, Nanjing Agricultural University, Nanjing, Jiangsu, P.R.China

[3] College of Economics and Management, Nanjing Agricultural University, Nanjing, Jiangsu, P.R.China; China Center for Food Security Studies, Nanjing Agricultural University, Nanjing, Jiangsu, P.R.China

* Corresponding author

E-mail: cchen@njau.edu.cn.

Funding Information：This article was sponsored by the Priority Academic Program Development of Jiangsu Higher Education Institutions (PAPD)

Abstract

Large input and high loss of chemical fertilizer are the main causes of agricultural non-point--source pollution in China. Employing fertilizer loss and micro-health data, this paper analyzes the effects of chemical fertilizer loss ~~from the soil~~ on the health of rural elderly ~~people in Chinese rural areas as well as~~ and the medical cost in China. Results of the difference-in-differences (DID) method ~~show~~ indicate that one kg/ha increase in fertilizer loss alters a key medical disability index (Activities of Daily Living) by 0.0147 and the number of diseases by 0.0057 for rural residents 65 and older. This is equivalent to CNY 316 million (USD 45 million) in national medical cost. Furthermore, the age of onset is younger in regions with higher fertilizer loss. One kg/ha increase of fertilizer loss advances the age of onset by 0.267 year, which will cause long term effect on public health. Our results are robust to variety of robustness checks.~~: (1) fertilizers negatively impact the health of the elderly in rural areas, in particular by reducing their ability on a daily basis and increasing the risk of disease; (2) the excessive use of chemical fertilizers also causes many diseases among young people; and (3) 1 kg/ha increase in chemical fertilizer loss unfavorably alters the index of dependence in activities of daily living by 0.0153 for rural residents aged 65 years and over. This is equivalent to a total extra cost increases of CNY 329 million (USD 7 million) one year. Agricultural technological innovation can fundamentally reduce the input of chemical fertilizer and reduce the loss from the soil to decrease the health risk of elderly people who are exposed to chemical fertilizer risk in rural China.~~

1. Introduction

China experienced rapid growth in chemical fertilizer use, particular after 2004 when the Chinese government shifted from taxing agriculture to subsidizing agricultural programs. The unit area chemical fertilizer usage is nowadays 4 times more than the world average [1]. Agricultural nonpoint-source pollution is a growing concern in China because these pollutions had become the main cause of water pollution

[2].According to the health production function theory, environmental pollution is considered to be an important factor affecting human health. Alberini finds that people living in heavily polluted areas are generally faced with the impact of an accelerated decline of healthy stock [1]. Currie stats that pollution near factories can lead to the depreciation of real estate and poor health [2]. Several studies reveal that the deterioration of river water quality not only causes the higher economic costs of diseases, but also increases the incidence rate of digestive tract cancer in China [3-4]. Agricultural non-point--source pollution is has been found to be an important factor affecting health[3-6]. Brainerd and Menon surmise that the concentration of chemical fertilizer in water can increase neonatal mortality and damage children's health [5]. Lai finds that pesticide usage reduced the health level of the elderly, increasing their medical and family care costs [6]. In addition to chemical fertilizers and pesticides, agricultural straw combustion is found to increase mortality by affecting air quality [7]. Specifically, the elderly might have a higher health risk comparing with the young people when facing environmental exposure. A higher susceptibility and higher mortality rates could be observed among the adults over the age of 75 years compared with the younger ages [7-9]. The differences of health risk might due to physiological changes associated with aging [10]; the age-related behavior trends varied between younger and older adults which might affect older adults' exposures [11]; other factors such as socio-economic and nutritional status [12]. China had the most serious aging problems, 70% of elderly live in rural areas [13], the elderly residents might most affect by agricultural nonpoint-pollution. Another reason we focus on the rural elderly is that the health status, medical facility and diseases awareness of rural elderly are not as good as those in cities [14-15]. This paper complements the existing literature by proving new evidence on the effects of agricultural non-agricultural pollution. Air pollution as a leading problem for public health was broadly studied in agricultural nonpoint pollutions [16-17]. The works most closely related to this paper were Brained and Menon, which assessed the impact of fertilizer runoff on infant and child in India, we complement their study by emphasizing another vulnerable group, i.e. older adults, who are more likely to stay in countryside in the process of urbanization and most directly affected by the loss of chemical fertilizer. Another work is Lai, which assessed the health effect of pesticide by using the pesticide cost. In order to eliminate the influence of price,

we use the amount of chemical input and loss, which are more accurate. Though both chemical fertilizers and pesticides could cause water pollution, they worked in different ways. Therefore, this study to explain the health effect of fertilizer loss is valuable. This paper emphasis on non-occupational fertilizer exposure, which is necessary because the farmers’ occupational risk via direct exposure of chemical fertilizer was only a small proportion of the population and had been studied [18-20]. Many more population may be at risk because of agricultural externality.

~~In China, agricultural non-point source pollution has become the main cause of water pollution [8]. According to the China Pollution Source Census (2010), the chemical oxygen demand (COD) emission of agricultural pollution sources was 1.8 times of the industrial sources [9]. The application intensity of chemical fertilizer in China is four times the world average level and about double the internationally recognized safe level. After 2004, in particular, China abolished agricultural tax and issued a series of agricultural support policies to promote agricultural production, which encouraged the excessive plication of agricultural fertilizer. From 2004 to 2015, the amount of chemical fertilizer applications was increased by 30% (i.e., 1.386 million tons) [10], while the increase in agricultural produce output was far less.~~

The inefficient use of chemical fertilizer has caused serious nonpoint-source pollution. Fertilizers leaded to "groundwater contamination, eutrophication of fresh water and estuarine ecosystems, tropospheric pollution related to emissions of nitrogen oxides and ammonia gas, and accumulation of nitrous oxide" [21]. A 10% increase in the use of nitrogen fertilizer (kg) and phosphorus fertilizer(kg) leaded to a 1.525% and 1.374% increase of the concentration of nitrogen (mg/l) and phosphorus (mg/l) in water, respectively [22]. In China, agricultural nonpoint-source pollution has exceeded industrial pollution in water, ~~According to the China Pollution Source Census (2010),~~ the chemical oxygen demand (COD) emission of agricultural pollution sources was 1.8 times of the industrial sources [23]. After 2004, in particular, China abolished agricultural tax and issued a series of agricultural support policies to promote agricultural production, which encourage the excessive application of agricultural fertilizer. From 2004 to 2015, the amount of chemical fertilizer applications was increased by 30% (i.e., 1.386 million tons) [24], while the increase in agricultural produce output was far less. ~~Under the conditions of rainfall and farmland irrigation, chemical pollution~~

~~from fertilizer may be transported from the soil circle to the water circle, causing water pollution. The health effects of fertilizer loss on the elderly are shown in Fig 1.~~ As a result of the formation of nitrite compounds and the change in soil environmental conditions, chemical fertilizer loss may potentially lead to chronic diseases, nervous system diseases, and organ diseases [~~11-13~~25-27], and eventually affect human health as well as medical cost (Fig 1). ~~eventually affect human health as well as medical cost.~~ Amines and amides in chemical fertilizers and sewage could~~an~~ cause digestive tract diseases, esophageal cancer, liver cancer, stomach cancer, esophagus cancer, and other cancers [~~14~~28-30~~16~~]. The fertilizer, which ~~are~~ were soluble in water and had~~ve~~ stable properties, were~~are~~ transported by rivers over long distances and large ranges, and enter the human body via drinking water and other ways to synthesize nitrosamines, especially nitrosamines. Drinking water or eating vegetables with a high nitrate content may lead to thyroid cancer, hypertension, neural tube defects (NTDs) [~~17-18~~31-32], and various chronic diseases, such as skin diseases, lung cancer, bladder cancer, and cardiovascular disease [~~19-20~~33]. Based on the background of China's agricultural support policy, this paper verifies the effect of chemical fertilizer loss from the soil on the health of the rural elderly, and comprehensively analyzes the health damage from the perspectives of daily living ability, disease risk, and disease age onset. The economic cost of this health damage is further calculated. More specifically, 3 hypotheses are proposed as follow: (1) Fertilizer loss can cause physical damage, increase the health risk and alter the daily living ability of the elderly. (2) Because of the physical function, fertilizer loss may lead the samples to be ill younger. (3) Due to the health damages, the medical cost will be higher in high-loss regions comparing the regions with lower fertilizer loss.

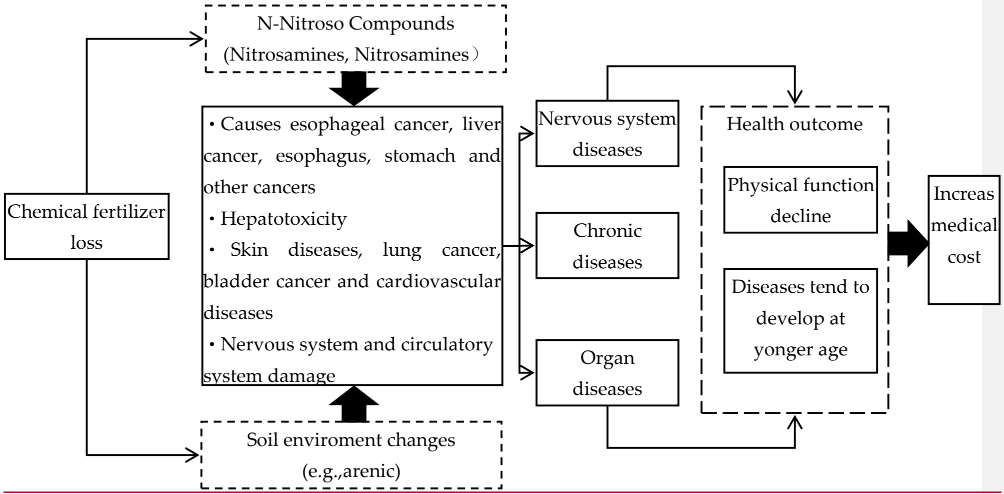

Fig1. Effects of Chemical Fertilizer Loss on Health. ~~This is the Fig 1 legend.~~

~~Based on the background of China's agricultural support policy, this paper verifies the effect of chemical fertilizer loss from the soil on the health of the rural elderly, and comprehensively analyzes the health damage from the perspectives of daily living ability, disease risk, and disease age onset. The analysis is accomplished using difference in differences method with the national dataset Chinese Longitudinal Healthy Longevity Survey (CLHLS) and publicly published yearbooks. The economic cost of this health damage is further calculated. More specifically, 3 hypotheses are proposed as follow: (1) Fertilizer loss can cause physical damage, increase the health risk of the elderly and affect the daily living ability of the elderly. (2) Because of the physical function, fertilizer may lead to the rejuvenation of disease. (3) Fertilizer loss may increase medical cost and there is regional heterogeneity. This paper contributes to existing literature from the following aspects: first, by studying China's rural elderly in the context of an aging society, this article enrich previous economic literature which estimates the health effecting using primarily pregnant women and infant data; secondly, agricultural support policy reform in 2004 is employed as a quasi-natural experiment to compare the health condition of rural elderly exposed to different fertilizer loss level before and after the policy reform; at last but not least, fertilizer loss data are analyzed in our study. Compared to previous studies which focused on fertilizer input, the relation between fertilizer loss and health is more accurate.~~

The rest of the paper is organized as follows. Section 2 provides data description and summary statistics. Section 3 presents empirical models as well as results and Section 4 concludes.

2. Data and s~~S~~ummary s~~S~~tatistics

2.1 Data c~~C~~ollection and v~~V~~ariables

The analysis is accomplished using difference in differences method with the national dataset Chinese Longitudinal Healthy Longevity Survey (CLHLS) and publicly published yearbooks.

~~This study matches micro-level individual health data and provincial-level chemical fertilizer input data to conduct the analysis.~~

The individual health data come~~s~~ from the Chinese Longitudinal Healthy Longevity Survey (CLHLS). CLHLS ~~was~~ is chosen for this research owning to ~~because of~~ its richness in health and demographic information, its nationwide sampling areas, and its time span~~length of time~~ which covers the year of the agricultural policy change. The CLHLS ~~This survey~~ was conducted in a randomly selected half of the counties and cities in 22 provinces, covering 85% of the total population in China. Its ~~has~~ eight waves ~~to data~~ (1998, 2000, 2002, 2005, 2008, 2011,2014 and 2018)~~and~~ surveyed the cohort of ~~aged~~ 65 years and older. We only use the data ~~after 1998 and before 2018~~from 2000 to 2014~~The data of 1998 is deleted~~ because the survey question ask~~s~~ings whether drinking water ~~is~~ was boiled or not started in 2000; only those who dr~~i~~ank boiled water are included in our analysis to eliminate possible health linkages to waterborne bacteria. China began to implement the "fertilizer reduction" policy in 2015, in order to eliminate the policy effect, we did not use the data after 2015. The survey combines an in-home interview and a basic physical examination. Extensive information was collected on demographic characteristics, family and household characteristics, lifestyles, diet, psychological characteristics, health, disability, socioeconomic conditions, etc. Fertilizer input data come from the China Rural Statistical Yearbook (1999-~~2019~~2015) and other regional variables come from the China Statistical Yearbook (1999-~~2019~~2015). Chemical fertilizer loss index come from the Technical report on China pollution source census (2011).

2.1.1 Health outcomes variables

Multiple indicators for health status of the elderly are examined linked to fertilizer loss in the extant medical and toxicology literatures, including the index of Activities of Daily Living (ADL), the numbers of diseases related to fertilizer loss, and age of ~~illness~~ onset.

The ADL index is widely used to measure the dependence of the elderly and physically inactive people. It is also known as the "disability" index [~~21~~34]. The ADL index is constructed by asking participants whether they need help with six basic activities (bathing, dressing, eating, going to the toilet, indoor movement, and controlling defecation). The choices for each item are "can do without help" (corresponding score is 1), "need help" (corresponding score is 2), and "need full help" (corresponding score is 3). The final score of the ADL index is the sum of all six items, ranging from 6 to 18. Higher scores mean the health status of the elderly is worse.

The number of diseases measures how many types of diseases related to fertilizer loss are suffered by a sample individual~~is the second health indicator~~. Although ~~T~~the CLHLS database investigated 16 diseases in the elderly, including hypertension, heart disease, cancer, stroke, cardiovascular and cerebrovascular diseases, bronchitis, emphysema, asthma, and pneumonia~~. However~~, only three diseases—hypertension, cancer, and gastrointestinal ulcer—were related to fertilizer loss in the existing medical literature.

The age of ~~illness~~ onset refers to the age at which a sample individual first ~~suffers~~ suffered from any of the three diseases (hypertension, gastrointestinal ulcer, or cancer).

2.1.2 Individual and household variables

Individual and household variables include information on socio-economic characteristics, health behavior and dietary pattern. Socio-economic characteristics include age, gender, people living together, income station, basic health condition and hospitalization. Health behaviors measure whether the respondents smoke, drink ~~and~~or do any exercise ~~now~~ (~~the answers are~~with options of "Yes" or "No"). Dietary patterns measure the frequency with which respondents eat meat, fish, egg, and salt-preserved vegetables. Options~~Answer~~ for each item are "~~“~~rarely or never"~~”~~, "~~“~~not every month, but occasionally"~~”~~,

"not every week, but at least once per month", "not every day, but at least once per week", "almost every day", with scores from 1 to 5, respectively. The questionnaire also asks the ~~re~~sources of respondents' drinking water. Options ~~The answers~~ include drinking water sources from a well, a river of lake, a spring, a pond or pool, and tap water. A binary variable (Water) is constructed, which equals one if respondents drink surface water, i.e. water from a rive or lake or a pond of pool, and equals zero otherwise.

2.1.3 Provincial variables

Provincial variables include fertilizer loss, fertilizer use, organic fertilizer use, pesticide use, industrial pollution index, the numbers of hospital and provincial Gross Domestic Product. The calculation method for fertilizer loss intensity is based on literature [~~22~~35]. On the basis of the unit investigation method and the inventory analysis method, the fertilizer loss coefficient method is used to calculate the total nitrogen and phosphorus emissions; then the total emission and emission intensity of fertilizer nonpoint-source pollution are calculated. The calculation formula is as follows:

$$E=\sum E_{ij}=\sum C_{ij}\times h_{ij} \quad (1)$$

$$I=E/A \quad (2)$$

where E measures the total emission of fertilizer loss; E_{ij} measures the amount of the pollutant j ~~(j)~~ produced and the loss into water by ~~pollution~~ pollutant i ~~unit (i)~~; C_i measures the index statistics of ~~pollution~~ pollutant i ~~units (i)~~(compound fertilizer is converted into total nitrogen 40% and phosphorus pentoxide 32%); η_{ij} measures the loss coefficient; A represents the crop areas; and I represents pollution emission per unit area, i.e., pollution intensity (unit: kg/ha).

The industrial pollution index ($Indpollution_i$) controls industrial activities, which defined in ~~formula~~ Equation (3) according to existing literature [~~23~~36-~~24~~37]. GDP measures general economy and wealth conditions. The number of hospitals controls the level of medical service. Because the health data are observed once three years, all provincial variables are assigned the average of the last three years.

$$Indpollution_i=\frac{pv_{i1}+pv_{i2}+pv_{i3}}{3},\quad pv_{ij}=p_{ij}\Big/\sum_{i=1}^{n}\frac{p_{ij}}{n} \quad (3)$$

where p_{ij} measures the pollutant_ j ~~(j)~~ of province i ~~(i)~~, n is the amount of province, pv_{ij} measures the emission index of pollutant_ j ~~(j)~~ in the province_ i ~~(i)~~ relative to the national average level. The larger value means the higher pollution level. $pv_{i1}, pv_{i2}, pv_{i3}$ represents industrial wastewater, industrial sulfur dioxide ~~SO_2~~ and industrial dust, respectively.

2.2 Statistical a~~A~~nalyses

2.2.1 Sample summary

Table 1. Variable description and summary statistics

Variables	Description	Obs.	Mean	Std.Dev.	Min	Max
**Household Variables**						
Age	Age in years	39093	85.83	10.83	65	109
Male	Male=1;Female=0	39093	0.44	0.50	0	1
Co-residence	Nursing home=1; Alone or Spouse=2; Child=3; Others=4	39093	2.62	0.57	1	4
Income_cost	If income support daily cost (Yes=1; No=0)	39093	0.78	0.41	0	1
Illness	Number of 15 chronic diseases	39093	0.85	1.06	0	1
Hospitalization	The times of hospitalization in two years	39093	0.26	0.79	0	30
**Health Behaviors**						
Smoke	Currently smoke (Yes=1; No=0)	39093	0.33	0.47	0	1
Drink	Currently drink (Yes=1; No=0)	39093	0.31	0.46	0	1
Exercise	Currently exercise (Yes=1; No=0)	39093	0.27	0.44	0	1
Dietary Pattern						
Meat	The frequency of meat consumption	39093	3.95	1.03	1	5
Fish	The frequency of fish consumption	39093	3.36	1.15	1	5
Egg	The frequency of egg consumption	39093	3.92	1.07	1	5
Salt_vege	The frequency of salt-preserved vegetable consumption	39093	3.27	1.38	1	5
Boiled water	If drink boiled water (boild water=0; not boild water=1)	39093	0.057	0.23	0	1
Water	Drirking water source (Surface water=1; Tap water=0)	39093	0.14	0.35	0	1
**Provincial Variables**						
Floss	Fertilizer loss intensity (kg/ha/year)	39093	7.34	3.76	0.60	14.82
Finput	Fertilizer input intensity (kg/ha/year)	39093	354.80	92.47	128.41	704.32
Pinput	Pesticide inptut intensity (kg/ha/year)	39093	12.62	6.56	2.28	22.26
OFinput	Organic fertilizer input intensity (kg/ha/year)	39093	286.53	361.18	11.56	1997.00
Hospnum	The n~~N~~umber of hospital per province (million)	39093	0.02	0.02	0.01	0.08
LnGDP	Logarithm of Provincial Gross Domestic Product	39093	9.10	0.91	7.32	11.04
Indpolltion	Index of industrial pollution (lower=better)	39093	1.46	0.55	0.45	2.95
**Health indicatos**						
ADL	Activities of Darly Living score (lower=better)	39093	6.66	1.80	6	18

No. of diseases	The nNumber of illness caused by fertilizer loss (lower=better)	39093	0.25	0.48	0	3
Age_ill	Age of suffering from illness caused by fertilzer loss (lower=worse)	9503	83.70	10.06	65	109
Mescost	Medical cost(RMB)	39093	2768.29	11676.91	0	199996

This is the Table 1 legend.

2.2.2 Fertilizer loss and health outcome after agricultural support policies

China shifted from taxing agriculture to subsidizing agricultural programs since 2004 to maintain food security and self-reliance. In 2004, authorities introduced three subsides targeted at grain producers: a direct payment for grain producers, a subsidy for improved seed varieties and a partial rebate for farm machinery purchases. Several other support programs were introduced such as a general-input subsidy, price floors for wheat and rice, reform of the grain marketing system and transfer payment to grain counties since 2004, which has been accompanied with greater fertilizer use and fertilizer loss. This set of supporting programs had bolstered farmer' production incentives and crop production increased continuously since 2004 [38-39], on the other hand, the programs might lead to the distortion of agricultural factors and cause the negative effect of soil and water environment [40]. Table 2 reports the differences of health outcomes and fertilizer loss between regions with high versus low intensity of fertilizer loss. We report the differences of ADL index, the numbers of induces diseases, intensity of fertilizer input, amount of fertilizer loss and intensity of fertilizer loss before the implementation of agricultural support policies in column (3). The differences after the agricultural support policies are in column (6). The widening gap in health outcomes and fertilizer loss and input can be observed in column (7) between regions with high and low fertilizer loss after the agricultural support policies. After the implementation of the agricultural support policies, the ADL index and the number of induced diseases were different between regions with high versus low intensity of fertilizer after the implementation of the agricultural support policies (Table 2, lines 1 and 2). Preliminary statistics show that the loss of chemical fertilizer damages public health and increases health risks. The input and loss of chemical fertilizer are significantly different before and after the implementation of the agricultural support policies: (1) before and after the implementation of the agricultural support policies, the input intensity of agricultural fertilizer in-

creased in both high-loss areas and low-loss areas (the national average value of fertilizer loss in China is calculated. The regions with higher loss than the average value are high-loss areas, and the regions with lower loss than the average value are low-loss areas), but increased more in high loss areas (line 3); and (2) after the implementation of the agricultural support policies, the total amount and intensity of chemical fertilizer loss were more serious in high-loss areas (lines 4 and 5). Preliminary data analysis shows that the loss of chemical fertilizer in high-loss areas was more serious after the agricultural subsidy policies.

Table 2. Comparison of high-loss-areas and low-loss areas by agricultural support policies

Variables	Before agricultural Support Policies			After Agricultural Support Policies			2nd difference (7)=(6)-(3)
	Low loss (1)	High loss (2)	1st difference (3)=(2)-(1)	Low loss (4)	High loss (5)	1st difference (6)=(5)-(4)	
ADL index	6.823	7.022	0.199	6.822	7.108	0.287	0.088
No. of diseases	0.160	0.211	0.051	0.255	0.340	0.085	0.035
Input intensity (kg/ha)	239.562	358.413	118.851	317.519	457.163	139.644	20.793
Total loss (10000 tons)	1.689	6.717	5.028	2.023	7.350	5.327	0.299
Loss intensity (kg/ha)	2.905	8.599	5.694	3.491	9.226	5.735	0.041

This is the table 2 legend.

[a]ADL index represents the health outcome, which is described in Section 2.2.1, and stands the same meaning in tables below.

[b]No. of diseases represents the number of diseases (hypertension, gastrointestinal ulcer, or cancer), which is which is described in Section 2.2.1, and stands the same meaning in tables below.

2.2.3 Age of disease onset and fertilizer loss

The CLHLS contains three diseases (hypertension, gastrointestinal ulcer and cancer) induced by fertilizer loss. The age, when one sample first suffers from any of the three diseases, is defined "“age of disease onset "refers to the age at which a sample individual first suffers from any of the three diseases (hypertension, gastrointestinal ulcer, or cancer). The age of onset comparisons across age groups represented by Fig 2. A significant difference of age of onset can be observed between the regions with high versus low intensity of fertilizer loss in younger-old groups (< 80 years) in Panel A of Fig 3. The age of onset is not significant different in the oldest-old samples.Fig2 shows the relationship between the age of illness onset in the elderly in different age groups, and the intensity of chemical fertilizer loss. Preliminary data analysis shows that the effect of chemical fertilizer on the age of disease is heterogeneous. It can be seen that: (1) for the elderly under 80 years of age, there is a significant difference in the age of illness onset in the areas with high chemical

fertilizer loss, and the age of illness onset in the elderly in the areas with high chemical fertilizer loss is lower than that in the areas with low chemical fertilizer loss; and (2) for the elderly over 80 years of age, there is no significant difference in the age of illness onset. The illness and sick of younger elderly can cause longer-time effects. Disease rejuvenation means less working times and more medical costs and family cares. And the trend will become more severe as the elderly age.

Panel A. Younger-old Samples Panel B. Oldest-old Samples

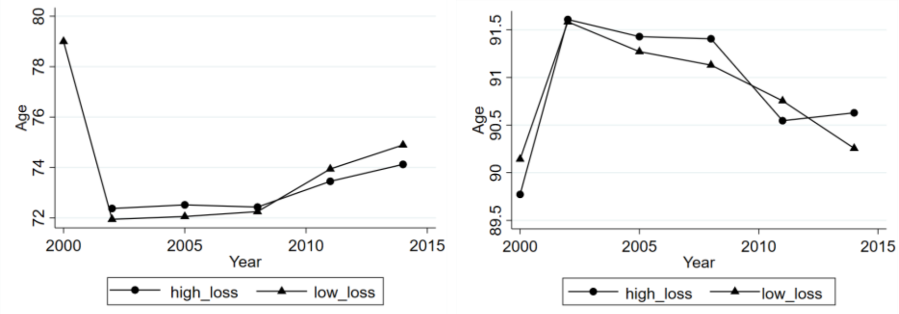

Fig 2. Chemical Fertilizer Loss and Age of Onset. This is the Fig 2 legend.

3. Empirical models and results

This section investigates the health effect of fertilizer loss on local elderly population. Preliminary data analysis reports the healthshows the differences in regions withhealth outcomes and high verse low intensity of fertilizer loss fertilizer loss between high-loss area and low-loss area after agricultural subside policies in 2004. Further, we follow a dDifference-in-dDifferences (DID) is frameworkadopted to compare health outcomes between people in regions with different intensitieshigh versus low intensity of fertilizer loss before and after 2004, when China shifted from taxing agricultural outputs to subsidizing agriculture. The medical cost between regions with high versus low intensity of fertilizer loss are estimated as well. To facilitate comparisons across age groups, explore heterogeneous effects by splitting the sample in various age groups and reporting estimates of α_1 and β_1 for these subsamples. Than Then several robustness

checks are conducted to help assess the validity of our results. The difference-in-differences DID model is specified as:

$$Health_{it} = \alpha_0 + \alpha_1 Fertilizerloss_{it} \times Time_{it} + \alpha_2 Fertilizerloss_{it} + \alpha_3 Time_{it} + \alpha_4 X_{it} + \gamma_t + \mu_i + \varepsilon_{it} \quad (4)$$

$$Health_{it} = \beta_0 + \beta_1 Area_{it} \times Time_{it} + \beta_2 Area_{it} + \beta_3 Time_{it} + \beta_4 X_{it} + \gamma_t + \mu_i + \varepsilon_{it} \quad (5)$$

$$Age_{it} = \delta_0 + \delta_1 Fertilizerloss_{it} \times Time_{it} + \delta_2 Fertilizerloss_{it} + \delta_3 Time_{it} + \delta_4 X_{it} + \gamma_t + \mu_i + \varepsilon_{it} \quad (6)$$

$$Age_{it} = \theta_0 + \theta_1 Area_{it} \times Time_{it} + \theta_2 Area_{it} + \theta_3 Time_{it} + \theta_4 X_{it} + \gamma_t + \mu_i + \varepsilon_{it} \quad (7)$$

where $Health_{it}$ denotes the health outcome; Age_{it} denotes the age of suffering from the disease above onset; α_1, β_1, δ_1 and θ_1 identify the effects of fertilizer loss; $Fertilizerloss_{it}$ denotes the emission of fertilizer loss of the region where individuals i resides; $Area_{it}$ is a dummy variable equalingdenotes the high and low area of fertilizer loss, *Area* equals 1 if the individual is in high loss areas, and 0 otherwise equals 0; $Time_{it}$ equals 1 starting in 2004 and 0 otherwise; X_{it} is a vector of control variables including individual, household, and provincial characteristics such as age, gender, health behavior, dietary habits, industrial pollution and hospitals, etc.; μ_i is a fixed effect unique to individuals i i; γ_t is a time effect common to all individuals in year t t; and ε_{it} is an error term.

The relationship between medical cost and health can be identified in the following Eequation:

$$Y_{it} = \alpha_0 + \alpha_1 ADL_{it} + \alpha_2 X_{it} + \lambda_t + \mu_i + \varepsilon_{it} \quad (8)$$

where Y_{it} represents medical costs, and other variables are as defined as before.

3.1 Basic regression results of health

3.1.1 Effect of fertilizer loss on health

Table 3 reports the α_1 and β_1 coefficients in Equation (4) and Equation (5) for outcomes related to ADL index and No. of diseases. Estimated coefficients in column (1) of Table3 indicate that one kg/ha increase of lists the impact of chemical fertilizer loss on the health of the rural elderly. From the results of the model, the loss of chemical fertilizer significantly increased the ADL index and the number of diseases, indicating that the loss of chemical fertilizer significantly increased the health risk for the rural elderly. Columns (1) and (2)

of the table show the impact of chemical fertilizer loss is associated with increased ADL daily life index of 0.0147. From column (4) one kg/ha increase of fertilizer loss is associated with on health. For every unit of increased chemical fertilizer loss that, the ADL daily life index increased by 0.0153 (significant at 1% level) and the number of induced diseases increased by 0.00575 raise in the number of diseases. These results indicate that the fertilizer loss increase the elderly's health risks and support diseases significantly. (significant at 1% level). Columns (3) and (4) show the model results after the samples were divided into high and low loss areas. For different regions, estimates for β_1 in columns (2) and column (5) of Table 3 indicate a reduction in the ADL index and the number of diseases for high-loss area verse low-loss areas. The results show that, Ccompared with low-loss areas, with low chemical fertilizer loss, the ADLdaily life index of the rural elderly in areas with high chemical fertilizer loss increases by is 0.1222 higher326 (significant at 1% level) in areas with high chemical fertilizer loss, while the number of diseases induced byis chemical fertilizer loss increases by 0.0487390 (significant at 1% level) higher. We also use the two-way fixed effects model to estimate the health effect of fertilizer loss. Column (3) and Column (6) of Table 3 report the coefficients for outcomes related to ADL index and No. of diseases. Estimated coefficients indicate that one kg/ha of fertilizer loss increase is associated with increased ADL daily life index of 0.1088. From column (6) one kg/ha of fertilizer loss increase is associated with 0.0211 raise in the number of diseases. The health effect impact of agricultural fertilizer input not only exists accrue in farmers who are likely to receive a fertilizer loss via direct exposure. Many more proportions of the population are at risk of fertilizer loss because of exposure to contaminated water and soil. Agricultural support policies play an important role in the fertilizer loss, and the agricultural may cause heterogeneous fertilizer loss between regions because of the different subsides levels. These may cause the biased result of TWFE, DID model can avoid this problem, and we control the time effect and individual effect in our regressions. the agricultural production system, but also has a significant impact on the health of the elderly outside the agricultural production system. Due to the existence of externality, agricultural fertilizer diffuses outside of agricultural production in the form of loss, resulting in a greater range of health issues. The loss of chemical fertilizer causes a change in the water and soil environment, which then

affects human health, causing disease and a decline in bodily function. Therefore, from the perspective of agricultural and public health policies, agricultural policy should take fertilizer loss into consideration and promote low loss production technology. In terms of public health policies, the government need to focus on the impact of agricultural fertilizer loss on health, particularly the two impacts of physical function and induced disease. The government need to start with the two aspects of information dissemination and medical policy to improve the health awareness and health status of the rural elderly.

Table 3. Basic Estimates ADL index and Diseases

Effect of Chemical Fertilizer Loss on the Health of the Elderly: Difference in Differences

	ADL			No. of diseases		
	DID		TWFE	DID		TWFE
	(1)	(2)	(3)	(4)	(5)	(6)
Fertilizerloss*Time	0.0147**	--	--	0.0057***	--	--
	(0.0060)	--	--	(0.0014)	--	--
Higharea*Time	--	0.1222***	--	--	0.0487***	--
	--	(0.0461)	--	--	(0.0114)	--
	--	--	0.1088**	--	--	0.0211*
	--	--	(0.0432)	--	--	(0.0126)
Control	YES	YES	YES	YES	YES	YES
Year FE	YES	YES	YES	YES	YES	YES
ID FE	YES	YES	YES	YES	YES	YES
Observations	32165	32165	38908	32329	32329	38680
R-squared	0.5570	0.5582	0.0843	0.6906	0.6907	0.3243

This is the table 3 legend.

[a] robust standard errors for clustering at the individual level are reported in brackets; *, **, *** are significant at the level of 10%, 5% and 1% respectively.

3.1.2 Heterogeneous health effects

In S1 and S2 Tables, we explore the heterogeneous effects by splitting the samples in different age groups and reporting estimates of α_1 for these subsamples. First, the oldest olds (≥80years) were discussed in previous studies [41], because the oldest olds had the greater risk of falling and hospitalization [42-44]. We divide the samples at age into the younger age samples (<80years) and the oldest olds samples (≥80 years). In Columns (1) and columns (2) of S1 Table, a positive and significantly estimate of α_1 can be observed in the oldest olds. The estimates of α_1 are positive and significantly for both age groups (columns (3) and columns (4) of S1 Table), the fertilizer loss will increase the risk of diseases in both the younger olds and the

oldest olds. The grouped estimates for effect of fertilizer loss on ADL and diseases of the oldest old are significantly distinguishable from the younger olds at the 1% level. We also divide the samples into younger olds (65years-75years), middle olds (75-85years) and the oldest olds (≥85years) [45-47]. The differences between younger and middle olds are not significant in both ADL and No. of diseases. The coefficient for oldest olds is larger than that for younger and middle olds, and the differences are significant.

3.1.~~2~~3~~.~~ Effect of fertilizer loss on Age of ~~Illness~~ Onset

Table 4 reports the δ_1 and θ_1 coefficients in Equation (6) and Equation (7) for outcomes related to age of onset. ~~Loss of chemical f~~Fertilizer loss increases the risk of disease in the rural elderly. This ~~paper~~ study further ~~discusses~~ explores whether ~~the loss of chemical~~ fertilizer loss will lead~~s~~ to the elderly be sick in younger age~~rejuvenation of disease~~. Though from Fig 2, the age of onset of elderly in areas with high-loss of chemical fertilizer are younger~~Although it can be seen from Fig 2, that the elderly people in areas with a high loss of chemical fertilizer are younger~~, in order to understand the ~~causal~~ relationship between ~~the loss of chemical~~ fertilizer loss and the age of onset~~the elderly people~~, ~~this paper~~we use~~s~~ the ~~double~~ difference-in-differences model for empirical analysis~~, .and the results are shown in Table 4.~~ Estimated coefficient in c~~C~~olumn (1) of Table 4 indicates that~~shows the average effect of chemical~~ one kg/ha increase of fertilizer loss advances the age by 0.2670 year. Comparing with low-loss areas, the age of onset is declines 0.3753 in high-loss areas. ~~on the age of the elderly after controlling the fixed effect of time and individual. The results show that the regression coefficient of chemical fertilizer loss on the age of the elderly is -0.277 (significant at the 5% level), indicating that the loss of chemical fertilizer significantly reduces the age of the elderly. Column (2) adds control variables to column (1), and the regression results remain unchanged. Column (3) shows the regression coefficients after clustering at the individual level, and the regression coefficients are essentially consistent, indicating that the model results are more robust. A younger age of illness onset may have two effects: (1) to further reduce the efficiency of agricultural production, as the elderly population is an important part of agricultural production, and a younger age of illness onset will reduce the labor efficiency of the elderly with a higher production efficiency, or may even result in their withdrawal from the labor market;~~

and (2) to increase the burden of medical care, as this will increase the medical cost and burden of the younger elderly. As they age, the younger elderly who are ill will face a faster deterioration in health, which will undoubtedly make the rural aging situation more difficult.

Table 4. Estimation of Effect of Chemical Ffertilizer Lloss on Age age of Onsetonset: Difference in Differences

	Age of Onset	
	(1)	(2)
Fertilizerloss*Time	-0.2670*<br>(0. 0161)	
Higharea*Time		- 0.3753**<br>(0. 1258)
Control Variables	YES	YES
Year FE	YES	YES
Individual FE	YES	YES
Observations	9503	9503
R-squared	0.9822	0.9822

This is the table 4 legend.

[a] *, **, *** are significant at the level of 10%, 5% and 1% respectively.

3.2 Results of health conditions on medical cost

In this section, we further explore these results by translating estimated effects into expected monetary losses. We do this by exploiting the relation between ADL and medical cost in Equation (8). Table 5 lists the effect of health conditions of the elderly on medical cost. The first column is the regression result of the overall sample. Estimated coefficient in column (1) of Table 5 indicates The results show that each additionalevery unit increase inof the ADL index increases the medical cost by CNY 244.6 (USD 34.94) (1% level is significant). Columns (2) and (3) report the λ_1 coefficient in Equation (8) in high-loss and low-loss areas, respectively. are the model results of areas with high and low chemical fertilizer loss, respectively. The estimatesp-value of the difference test is 0.0853 (10% level is significant), indicating indicate that there is a significant difference in medical cost between high loss areas and low loss areas, every one-unit ADL increase is associated with the medical costwith high loss areas being CNY 194.64 (USD 27.81) higher in high loss areas than low loss areas. The health cost per unit loss of chemical fertilizer (1kg/ha) is about CNY 3.674 (USD 0.5351). According to the sixth census, there wereare about 87.91 million rural elderly people (over 65 years of age) in China, and the loss of chemical fertilizer per unit will add increase medical costs by CNY 31629 (USD 457) million. There are about 4.4 million elderly people (65 years of age and above) in rural areas

with a high loss of chemical fertilizer. Compared with low loss areas, the medical cost of high loss areas is about CNY 114 (USD 16.29) million more. Though aAgro-chemical use is associated with significantly greater agricultural output value, a range studies showed that agro-chemical also damaged human health. Indirectly, medical cost increases and labor supply loss could be observed due to illnessbut also costly from the standpoint of a range of human health outcomes negatively associated with agro-chemical use and, indirectly, labor supply due to time lost to illness [2548]. Similar to our study, Lai suggested thatIn China, a 10%(CNY 0.2/USD 0.03) increase in rice pesticide use will add 168.8 and 55.89 million dollars to medical costs and offspring's human capital losses, respectively [6]. Compared with the pesticide use, we estimate the economic loss of fertilizer loss, an increase of one1kg/ha of fertilizer loss and fertilizer input will add CNY 316 (USD 45) million and CNY 21.5 (USD 3.1) million to national medical cost, respectively.

Table 5. Effect of health on medical cost

	Medical Cost		
	Overall samples	High-loss area	Low-loss area
ADL index	244.60***	331.94***	137.30**
	(55.81)	(93.54)	(63.58)
Control Variables	YES	YES	YES
Year FE	YES	YES	YES
Individual FE	YES	YES	YES
Observations	23087	19875	15167
R-squared	0.0709	0.0226	0.0121
p			0.0853*

This is the table 5 legend.

[a]*, **, *** are significant at the level of 10%, 5% and 1% respectively.

3.3 Robustness cCheck

3.3.1 Parallel tTrend tTest

In order to test that the time staggered entry events of agricultural policy are effective exogenous shocks, this study makes a parallel test. We divide the samples to five sections: the first period before the policy implementation, the second period after policy, the second two periods after the policy implementation to the end of sample period. Thus, five indicator variables pre_1, post_1, post_2, post_3 and post_4 are constructed. S1 Fig, Panel (1) plots estimates of ADL index and Panel plots estimates of No. of diseases. The set of pre_1 coefficient is statistically indistinguishable from zero, but there is a sharp, statistically significant and sustained change after the policy implementation.

3.3.2 Placebo t~~T~~est

In order to further test whether the results ~~of this study~~ are driven by unobservable factors ~~at~~ of the individual and provinc~~e~~ial level, referring to the methods of existing literature [~~26~~49-~~27~~50], this study conducted a placebo test by randomly assigning high and low chemical fertilizer loss areas. Specifically, 9 out of 19 provinces ~~were~~ are randomly selected as the experimental group, assuming that these 9 provinces are areas with high loss of chemical fertilizer, and other regions are the control group (areas with low loss of chemical fertilizer). Random sampling ~~ensured~~ ensures that the model constructed in this paper ~~had~~ has no effect on the health level of the elderly. In this study, random sampling ~~was~~ is carried out ~~500~~ 1000 times, and the benchmark regression ~~was~~ is performed according to Equation (~~4~~5). S2 Fig ~~3~~ reports the average value of the ADL index and the number of induced diseases after ~~500~~ 1000 random distributions. The mean value of ~~500~~1000 random sample regressions is almost 0. The ~~results~~ placebo test shows that ~~the~~ our basic estimation results are unlikely to be driven by unobservable factors at the individual and provinci~~al~~e level.

~~**Fig 3. Placebo Test.** This is the Fig 3 legend.~~

3.3.3 Effect of fertilizer input on health

In order to verify the robustness of the research conclusions, the application intensity of chemical fertilizer and the areas with high and low chemical fertilizer application intensities ~~were~~are used to test the robustness. According to the construction index of ecological counties in China (the application intensity of chemical fertilizer should not exceed 250 kg/ha), the areas with a chemical fertilizer application intensity exceeding 250 kg/ha are defined as high input areas, and the areas with a chemical fertilizer application intensity not exceeding 250 kg/ha are defined as low input areas. The basic regression of Equations (~~3~~4) and (~~4~~5) are~~was~~ carried out using the chemical fertilizer application intensity. The model results are shown in S3 Table ~~6~~. The model results are essentially consistent with the benchmark regression results, indicating that the research results are robust. For every 1 kg/ha increase in fertilizer input intensity, the ADL index will increase

by 0.001 0 (significant at 1% level), and the number of induced diseases will increase by 0.0001 (significant at 10% level).

3.3.4 Effect in different areas

In order to verify the robustness of the conclusions of this study, different samples were are used to test the robustness. This paper focuses on the impacthealth effect of agricultural fertilizer loss on the health of the rural elderly. Therefore, samples data from major agricultural-producing areas, major rice-producing areas, andareas and water resource-rich areas were are used to carry out the basic regression of Equation (4) and Equation (5). The results (S5 and S6 Tables)are shown in Table 6. The results show that fertilizer loss has lead a significant negative effectincrease both on ADL index and the number of diseases in major agricultural-producing areas, major rice-producing areas and water resource-rich areas, which are basically same comparing with the basic regression results in table7table3. The basic regression results are robust to a variety of robustness checks.

Table 7. Effect of Chemical Fertilizer loss on the Health of the Elderly in different areas

Variables	Major Agri-producing areas		Major Rice-producing areas		Water resource rich areas	
	ADL index	No. of diseases	ADL index	No. of diseases	ADL index	No. of diseases
Fertilizerloss*Time	0.180**	0.043**	0.110*	0.035**	0.066	0.033**
	(0.076)	(0.017)	(0.064)	(0.014)	(0.072)	(0.016)
Control Variables	YES	YES	YES	YES	YES	YES
Year FE	YES	YES	YES	YES	YES	YES
Individual FE	YES	YES	YES	YES	YES	YES
Observations	21063	21063	25760	25760	24223	24223
R-squared	0.608	0.703	0.598	0.682	0.597	0.671

This is the table 7 legend.

[a]*, **, *** are significant at the level of 1%, 5% and 10% respectively.

4. Conclusions and dDiscussion

China's agricultural fertilizer input is facing a problem of "high input, high loss, and high pollution, which ~~has~~ had been aggravated by agricultural support policies since 2004. The problem of the "T~~t~~hree H~~h~~ighs" of chemical fertilizer use has caused pollution to the environment and health damage, many more rural residents may be at health risk because of fertilizer loss.~~with the impact of chemical fertilizer loss transferring from within agricultural production to outside agricultural production.~~ In this paper, the chemical fertilizer loss data and CLHLS micro-health data are used to verify the health effect of fertilizer loss of the rural elderly. ~~Fertilizer loss will reduce the health and increase medical cost. China has the most serious aging, 70% of elderly live in rural area [28].~~ China had the most serious aging problem; it was estimated that the number of the elders might increase to 450 million (nearly one third of the total population) by 2050. Nearly 60% elders live in rural areas [51]. The health decline caused by fertilizer loss not only ~~increases~~ increased the medical cost but also affected the economics, e.g. reducing labor time, increasing family care time and so on [~~29~~52-53~~30~~]. The health effect of fertilizer maybe a greater challenge to pension and health care in rural China. To improve the health station of elderly in rural areas, the government can consider from agricultural policy and public health policy. (1) reduce fertilizer loss by innovating agricultural production technology research and promoting technology adoptions. e.g. soil testing, formula fertilization technology, slow-release fertilizer technology, deep tillage machinery promotion, and straw returning technology. (2) as the loss of chemical fertilizer includes not only the loss from the current year, but also the basic loss of ~~basic~~ fertilizer, the government should improve soil quality to increase nutrient preserving capability of soil,~~focus on the technologies that reduce the loss of basic fertilizer in technology development and promotion~~, such as soil improvement technology,~~ application of organic fertilizer,~~ etc.~~, to increase the content of organic matter in the soil and improve the fertilizer conservation capacity.~~ (3) drinking water is an important way of fertilizer loss affect the public health, so the government need to focus on the rural drinking water, improve the rural elderly's awareness of drinking safety and the health damages of chemical fertilizer loss.

The health effects of agricultural nonpoint~~-~~-source pollution ~~were~~ are estimated by taking the fertilizer as an example. Due to the limitations of the article, this article ~~did~~ does not discuss the health effect of other

agricultural pollution, such as pesticides use, straw combustion and the interaction of various agricultural pollutions. The nonpoint--source pollution in animal husbandry and fishery is also worth to discuss.

References

1. NBSC. 2016. China Statistical Yearbook. Beijing, National Bureau of Statistics of China.
2. Zhou K, Fan J, Liu HC. 2017. Spatiotemporal patterns and driving forces of water pollutant discharge in the Bohai Rim region. Progress in Geography. 2020; 36(02): 171-181.
3. Sun XY, Wang J, Jin YT. Advances in research on pesticide pollution to the aquatic environment and health impact in China. Journal of Environment and Health. 2009;26(07): 649-652.
4. Brainerd E, Menon N. Seasonal effects of water quality: The hidden costs of the green revolution to infant and child health in India. Journal of Development Economics. 2014; 107: 49-64. doi: 10.1016/j.jdeveco.2013.11.004.
5. Lai WY. Pesticide use and health outcomes: evidence from agricultural water pollution in China. Journal of Environmental Economics and Management. 2017; 86: 93-120. doi:10.2139/ssrn.2751458.
6. He GJ, Liu T, Zhou MG. Straw burning, PM 2.5, and death: Evidence from China. Journal of Development Economics. 2020;145: 102468. doi: 10.1016/j.jdeveco.2020.102468.
7. Wong CM, Lai HK, Tsang H, Thach TQ, Thomas GN, et al. Satellite-based estimates of long-term exposure to fine particles and association with mortality in elderly Hong Kong residents. Environmental Health Perspectives. 2015;123(11):1167–1172. doi: 10.1289/ehp.1408264. Epub 2015 Apr 24. PMID: 25910279.
8. Sacks JD, Stanek LW, Luben TJ, Johns DO, Buckley BJ, et al. Particulate matter-induced health effects: who is susceptible? Environmental Health Perspectives. 2011;119(4):446-54. doi:10.1289/ehp.1002255. PMID: 20961824.
9. Cohen, G., Gerber, Y. Air pollution and successful aging: Recent evidence and new perspectives. Current Environmental Health Reports. 2017; 4:1-11. https://doi.org/10.1007/s40572-017-0127-2. PMID: 28101729.
10. Assembly of First Nations (AFN). Environmental health older adults and seniors (elders). Ottawa, Canada. 2009.
11. Tuttle L, Meng Q, Moya J, Johns DO. Consideration of age-related changes in behavior trends in older adults in assessing risks of environmental exposures. Journal of Aging and Health. 2013; 25(2):243-273. doi:10.1177/0898264312468032. PMID: 23223208
12. Health Canada. Health of older adults and the environment discussion paper. Ottawa, Canada. 2008.
13. Tong YF. Changes and challenges of labor supply in China in the context of population ageing. Population Research. 2014; 38(02): 52-60.
14. Li JX, Li CH. Health difference of the elderly between the rural and urban districts. Population Journal. 2014; 36(05):37-47.
15. Weeks WB, Kazis LE, Shen TJ, Cong ZX, Ren XH, et al. Differences in health-related quality of life in rural and urban veterans. American Journal of Public Health. 2004; 10:1762-1767. https://doi.org/10.2105/AJPH.94.10.1762.
16. Khanal SJ, Pokhrel RP, Pokharel B, Becker S, Giri B, et al. An episode of transboundary air pollution in the central Himalayas during agricultural residue burning season in North India. Atmospheric Pollution Research. 2022; 13(1): 101270. https://doi.org/10.1016/j.apr.2021.101270.

17. Huang L, Zhu YH, Wang Q, Zhu AS, Liu ZY, et al. Assessment of the effects of straw burning bans in China: Emissions, air quality, and health impacts. Science of The Total Environment. 2021; 789: 147935. https://doi.org/10.1016/j.scitotenv.2021.147935.
18. Nganchamung T, Robson M. Chemical fertilizer use and acute health effects among Chili farmers in Ubon Ratchathani Province, Thailand. Journal of Health Research.2017; 31(6): 427-435. doi:10.14456/jhr.2017.53
19. Gorman NM, Stjernberg E, Koehoorn M, Demers PA, Winters M, et al. Fertilizer use and self-reported respiratory and dermal symptoms among tree planters. Journal of Occupational and Environmental Hygiene. 2013; 10(1): 36-45. doi: 10.1080/ 15459624.2012.740994.
20. Qiao F, Huang J, Zhang L, Rozelle S. Pesticide use and farmers' health in China's rice production. China Agricultural Economic Review. 2012; 4: 468-484. https://doi.org/10.1108/17561371211284821.
21. Zhang WD, Sohngen B. Do U.S. anglers care about harmful algal blooms? A discrete choice experiment of Lake Erie recreational anglers. American Journal of Agricultural Economics, Agricultural and Applied Economics Association.2018; 100(3): 868-888. https://doi.org/10.1111/ajae.12130
22. Paudel J, Crago CL. Environmental externalities from agriculture: Evidence from water quality in the United States. American Journal of Agricultural Economics. 2021; 103: 185-210. https://doi.org/10.1111/ajae.12130
23. China pollution source census. Technical report on China pollution source census. Beijing: China Environmental Press, 2011.
24. National Bureau of Statistics. China statistical yearbook (2019). Beijing: China Statistics Pres, 2020.
25. Zahra, Bahadoran, Asghar. Nitrate-nitrite-nitrosamines exposure and the risk of type 1 diabetes: A review of current data. World Journal of Diabetes. 2016; 7:433. doi: 10.4239/wjd.v7.i18.433. PMID: 27795817
26. Fan AM, Steinberg VE. Health implications of nitrate and nitrite in drinking water: An update on methemoglobinemia occurrence and reproductive and developmental toxicity. Regulatory Toxicology and Pharmacology.1996; 23:35-43. doi:10.1006/rtph.1996.0006.
27. Kristensen P, Andersen A, Irgens LM. Testicular cancer and parental use of fertilizers in agriculture. Cancer epidemiology, biomarkers & prevention. 1996; 5:3-9. PMID: 8770459
28. Ward MH. Too much of a good thing? Nitrate from nitrogen fertilizers and cancer. Reviews on Environmental Health. 2009; 24, 357-363. doi:10.1515/REVEH.2009.24.4.357.
29. Forman D. Are nitrates a significant risk factor in human cancer? Cancer Surveys.1989; 8(2): 443-458. PMID: 2696589
30. Bivolarska A, Gatseva P. Thyroid status in pregnant women and association with nitrates as an environmental factor stimulating the manifestation of iodine deficiency. Trace Elements and Electrolyte. 2015; 32:60-64. doi: 10.5414/tex01365.
31. Manasaram DM, Backe LC, Moll DM. A review of nitrates in drinking water: Maternal exposure and adverse reproductive and developmental outcomes. Environmental Health Perspectives. 2006; 114: 320-327. doi:10.2307/3436672.
32. Uddin M.S, Kurosawa K. Effect of chemical nitrogen fertilizer application on the release of arsenic from sediment to groundwater in Bangladesh. Procedia Environmental Sciences. 2011; 4: 294-302. doi: 10.1016/j.proenv.2011.03.034.
33. Smith AH, Hopenhayn-Rich C, Bates MN. Cancer risks from arsenic in drinking water. Environmental Health Perspectives. 1992; 97:259. doi: 10.1289/ehp.9297259.
34. Johnson RJ, Wolinsky FD. The structure of health status among older adults: disease, disability, functional limitation, and perceived health. Journal of Health Social Behavior. 1993; 34: 105-121. doi:10.2307/2137238
35. Lei J, Su SP, Yu WM. Temporal and spatial pattern evolution and grouping prediction of non-point source pollution of chemical fertilizers in China. Chinese Journal of Eco-Agriculture. 2020; 28(07): 1079-1092. doi: 10.13930/j.cnki.cjea.190923.
36. Shen KR, Jin G, Fang X. Dose environmental regulation cause pollution to transfer nearby? Economic Research Journal. 2017; 52(05): 44-59.

http://www.cesgw.cn/cn/mlInfo.aspx?m=20170217100514860651&n=20170609120846613735&tip=4.
37. Zhu FP, Zhang ZY, Jiang GL. Empirical study of the relationship between FDI and environmental regulation: An intergovernmental competition perspective. Economic Research Journal. 2011; 46(06): 133-145. http://www.cesgw.cn/cn/mlInfo.aspx?m=20110125121729123417&n=20110617154203400510&tip=10
38. Chen HP, Wu LP, Wang YB. Impacts of subsidy policy on grain production: An empirical analysis based on sub-provincial data of grain production from 2004—2007. Journal of Agro technical Economics. 2010; 4: 100-106.
39. Chen F, Fan QQ, Gao TM. Agricultural policies, food production and food production-adjustment ability. Economic Research Journal. 2010; 45(11): 101-114+140.
40. Ge JH, Zhou SD. An analysis of the economic determinants to agricultural non-point source pollution: Based on data of Jiangsu province during 1978-2009. Chinese Rural Economy, 2011; (5): 72-81.
41. Callaway B, Goodman-Bacon A, and Pedro H.C. Sant'Anna. Difference-in-differences with a continuous treatment. Working Paper, 2021.
42. Brettschneider C, Hajek A, Rohr S, Fuchs A, Weeg D, et al. Determinants of health-care costs in the oldest-old in Germany. The Journal of the Economics of Ageing. 2019; 14: 100200. https://doi.org/10.1016/j.jeoa.2019.100200.
43. Winograd CH. Targeting Strategies: An overview of criteria and outcomes. Journal of the American Geriatrics Society. 1991; 39:25-35. https://doi.org/10.1111/j.1532-5415.1991.tb05930.x
44. Speechley M, Tinetti M. Falls and injuries in frail and vigorous community elderly persons. Journal of the American Geriatrics Society. 1991; 39:46-52. doi: 10.1111/j.1532-5415. 1991.tb05905. x. PMID: 1987256.
45. Rockwood K, Stadnyk K, MacKnight C, McDowell I. A brief clinical instrument to classify frailty in elderly people. The Lancet. 1999; 353:205-206. doi: 10.1016/S0140-6736(98)04402-X. PMID: 9923878.
46. Lau LK, Wee SL, Mallya JU, Yap, Pang, et al. Physiological and cognitive determinants of gait in middle-aged, older-aged and oldest-aged Asian adults – The Yishun study. Aging and Health Research. 2021; 1(3): 100030. https://doi.org/10.1016/j.ahr.2021.100030.
47. Monti A, Doulazmi M, Nguyen-Michel VH, Pautas E, Mariani J, et al. Clinical characteristics of sleep apnea in middle-old and oldest-old inpatients: symptoms and comorbidities. Sleep Medicine. 2021; 82: 179-185. https://doi.org/10.1016/j.sleep.2021.04.017.
48. Sheahan M, Barrett CB, Goldvale, C. The unintended consequences of agricultural input intensifycation: human health implications of agro-chemical use in Sub-Saharan Africa. Working Paper Series. 2016; 234. https://www.afdb.org/en/documents/document/working-paper-234-the-unintended-consequences-of-agricultural-input-intensification-human-health-implications-of-agro-chemical-use-in-sub-saharan-africa-87634.
49. Cai XQ, Lu Y, Wu MQ. Does environmental regulation drive away inbound foreign direct investment? evidence from a quasi-natural experiment in China. Journal of Development Economics. 2016; 123: 73-85. doi: 10.1016/j.jdeveco.2016.08.003.
50. Ferrara LE, Chong A, Duryea S. Soap operas and fertility: evidence from Brazil. Applied Economics. 2012; 4: 1-31. doi:10.2139/ssrn.1820921.
51. Dai BZ. The old age health security in rural China: where to go? International Journal for Equity Health. 2015; 14: 119. doi: 10.1186/s12939-015-0224-5.
52. Mitra S, Gao Q, Chen W, Zhang YL. Health, work, and income among middle-aged and older adults: a panel analysis for China. The Journal of the Economics of Ageing. 2020; 17:100255. doi: 10.1016/j.jeoa.2020.100255.
53. Behncke, S. Does retirement trigger ill health? Health Economics. 2012;21: 282-300. doi:10.1002/hec.1712.

Supporting information

Formatted: Level 1

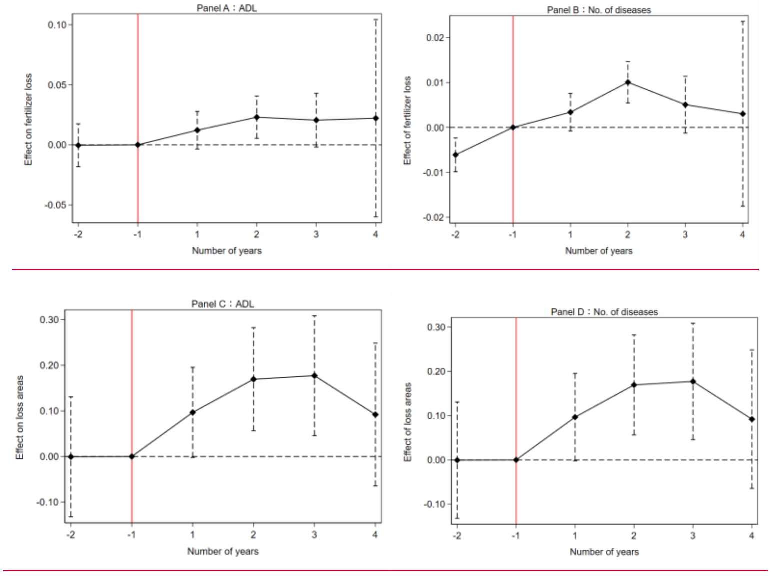

S1 Fig Parallel Trend Test

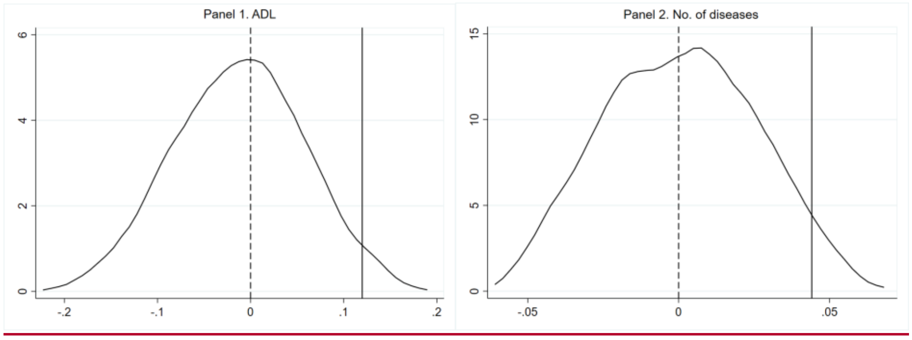

S2 Fig Placebo Test.

Panel 1 is the result of the ADL index placebo test; Panel 2 is the result of the placebo test of the number of patients. The X-axis represents the estimated coefficients from 1000 random assignments. The curve is the estimated kernel density distribution. The vertical type is a true estimate in columns (2) and (4) of Table 3.

S1 Table Basica regression results without control variables

	ADL		No. of diseases	
	(1)	(2)	(3)	(4)
Fertilizerloss*Time	0.0175***		0.0052***	
	(0.0057)		(0.0016)	
Higharea*Time		0.1608***		0.0441***
		(0.0432)		(0.0124)
Year FE	YES	YES	YES	YES
ID FE	YES	YES	YES	YES
Observations	32,302	32,302	32,467	32,467
R-squared	0.5474	0.5475	0.5597	0.5597

[a]*, **, *** are significant at the level of 10%, 5% and 1% respectively.

S2 Table Heterogeneous estimation of yonger olds and oldest olds

	(1)	(2)	(3)	(4)
	ADL		No. of diseases	
	Younger olds	Oldest olds	Younger olds	Oldest olds
Fertilizerloss*Time	-0.0006	0.0356***	0.0033**	0. 0086***
	(0.0038)	(0.0074)	(0.0014)	(0.0114)
Control	YES	YES	YES	YES
Year FE	YES	YES	YES	YES
ID FE	YES	YES	YES	YES
Observations	20536	22367	20620	22431
R-squared	0.0761	0.1359	0.3418	0.2951
p	0.0008***		0.0204**	

[a]*, **, *** are significant at the level of 10%, 5% and 1% respectively.

S3 Table Heterogeneous estimation of yonger olds, middle olds and oldest olds

	(1)	(2)	(3)	(4)	(5)	(6)
		ADL			No. of diseases	
	Younger olds	Middle olds	Oldest olds	Younger olds	Middle olds	Oldest olds
Fertilizerloss*Time	0.0027	0.0000	0.0334***	0. 0018	0.0039*	0. 0074***
	(0.0040)	(0.0061)	(0.0071)	(0. 0022)	(0. 0021)	(0.0114)
Control	YES	YES	YES	YES	YES	YES
Year FE	YES	YES	YES	YES	YES	YES
ID FE	YES	YES	YES	YES	YES	YES
Observations	8332	10719	23852	8360	10769	23922
R-squared	0.0548	0.0885	0.1287	0.3416	0.3398	0.2954
p	P^{12}=0.7163			P^{45}=0.4969		
	P^{23}=0.0039***			P^{56}=0.1936		
	P^{13}=0.0029***			P^{46}=0.0419**		

[a]*, **, *** are significant at the level of 10%, 5% and 1% respectively.

S4 Table Robustness check of fertilizer input and fertilizer input areas

	ADL		No. of diseases	
	(1)	(2)	(3)	(4)
Fertilizer input*Time	0.0010***		0.0001*	
	(0.0003)		(0.0001)	
Higharea*Time		0.2168***		0.0061
		(0.0547)		(0.0148)
Control	YES	YES	YES	YES
Year FE	YES	YES	YES	YES
ID FE	YES	YES	YES	YES
Observations	32443	32278	32443	32443
R-squared	0.6905	0.5646	0.6905	0.6903

[a]*, **, *** are significant at the level of 10%, 5% and 1% respectively.

S5 Table Additional robustness check of fertilizer loss in different areas

	Major Agri-producing areas		Major Rice-producing areas		Water resource rich areas	
	ADL index	No. of diseases	ADL index	No. of diseases	ADL index	No. of diseases
Fertilizerloss*Time	0.0283***	0.00200	0.0203***	0.00500***	0.0274	0.0161***
	(0.0082)	(0.0021)	(0.0066)	(0.0017)	(0.0175)	(0.0043)
Control Variables	YES	YES	YES	YES	YES	YES
Year FE	YES	YES	YES	YES	YES	YES
Individual FE	YES	YES	YES	YES	YES	YES
Observations	16880	16959	21528	21603	20526	20604
R-squared	0.572	0.695	0.549	0.677	0.533	0.667

[a]*, **, *** are significant at the level of 10%, 5% and 1% respectively.

S6 Table Additional robustness check of fertilizer loss areas in different areas in different areas

	Major Agri-producing areas		Major Rice-producing areas		Water resource rich areas	
	ADL index	No. of diseases	ADL index	No. of diseases	ADL index	No. of diseases
Higharea*Time	0.255***	0.0389**	0.163***	0.0452***	0.144**	0.0339**
	(0.0677)	(0.0171)	(0.0567)	(0.0141)	(0.0613)	(0.0158)
Control Variables	YES	YES	YES	YES	YES	YES
Year FE	YES	YES	YES	YES	YES	YES
Individual FE	YES	YES	YES	YES	YES	YES
Observations	16880	16959	21528	21603	20526	20604
R-squared	0.571	0.695	0.549	0.677	0.532	0.667

[a]*, **, *** are significant at the level of 10%, 5% and 1% respectively.

1. High loss areas include Anhui, Shandong, Jiangsu, Guangdong, Hebei, Henan, Zhejiang, Hubei and Fujian, while low loss areas include Chongqing, Sichuan, Shaanxi, Guangxi, Hunan, Jiangxi, Heilongjiang, Jilin, Liaoning and Shanxi. In this paper, data from Beijing, Tianjin and Shanghai are deleted because of the small sample size and the small agricultural planting area.

2. Data source of loss coefficient: technical report of the first general survey of pollution sources.

3. The major agricultural-producing areas are Hebei Province, Inner Mongolia Autonomous Region, Heilongjiang Province, Jiangsu Province, Anhui Province, Shandong Province, Henan Province, Hubei Province, Hunan Province and Sichuan Province.

The major rice-producing areas are Heilongjiang Province, Jiangsu Province, Anhui Province, Jiangxi Province, Hubei Province, Hunan Province, Guangdong Province, Guangxi Zhuang Autonomous Region, Chongqing and Sichuan Province.

The water resource rich areas are Heilongjiang Province, Zhejiang Province, Anhui Province, Fujian Province, Jiangxi Province, Hubei Province, Hunan Province, Guangdong Province, Guangxi Zhuang Autonomous Region and Sichuan Province.

Response to Editor

Dear Dr. Qiu,

Thank you for coordinating, reviewing, and extending the invitation to revise our manuscript. We have treated your as well as the reviewers' comments seriously and have significantly modified portions of the manuscript. For your advice, we have paid specific attention to meeting PLOS ONE's style and reporting associations in our manuscript. We summarize revisions we have implemented following the two reviewers' comments. Point by point responses are in separate files addressing to them respectively.

For Reviewer1, we have:

1. Improved writing and readability by simplifying some sentences, proving a better expression, and adding additional reading aid.
2. Added a series of publications in introduction and strengthened our contributions by comparing closely related literatures.
3. Enriched the content of empirical model of by adding regression results.

For Reviewer2, we have:

1. Compared the heterogeneous health risk of environmental exposure by adding the empirical analysis of different age groups and discussion in more detail.
2. Explained the hypotheses more clearly and explained the agricultural support polices in more detail.
3. Revised the description of the regression coefficients.
4. Offered the parallel trend assumption and discussed consistent estimation of Difference in Differences with a Continuous Treatment.
5. Improved writing and readability by including an additional figure, citing more recent publications, offering better variable definitions, and adding additional reading aid.

We hope you find our revised manuscript acceptable for publication in PLOS ONE.

Sincerely,

Ying Wang

Reviewer: 1

Thank you for the constructive comments on our manuscript. We have taken your comments seriously and substantially modified portions of the manuscript. We believe our work has been significantly improved as a result of your comments. Please find below our response to each point you made with your original comments in italic.

This paper focuses on an important and interesting topic of fertilizer exposure/application and human health. Although many efforts are put into this work, the paper still needs some improvement regarding methodology and organization/formatting of the paper. The writing and language should also be improved significantly before being published.

General comments

1. At the beginning of the introduction, a paper usually starts with background information and introduces the issue addressed by the paper. The first paragraph is not clear and interesting enough by listing some findings.

Response： Following your advice, we have significantly revised the first paragraph. First we introduced the background of agricultural non-point source pollution and fertilizer use in China. Second, added more publications to explain health effect of the agricultural non-point pollution Third, we explained the elders would be more susceptible, and the background of aging in China. This was the reason we focused on the elderly residences in rural China. Last, we have strengthened our contributions by comparing with two works most closely related to our study. The first paragraph was clearer and more logical.

2. Line 80 The contribution of the paper should be backed up with a thorough literature review. Please add more references and discussion on previous studies that are closely relevant to your study.

Response: Thank you very much for your suggestion. We have added some references about agricultural non-point pollution on public health, i.e. pesticide and straw burning in Line41-42, Line52-53. We have cited previous studies relevant to our study to back up the contribution of this paper in Line54-59.

3. Line 98 Can you have a descriptive map showing the provinces and use different color shades to show the levels of fertilizer application/exposure or heath level?

Response: Thank you very much for your suggestion. We dichotomized fertilizer loss by its pre-policy mean. The national average value of fertilizer loss is 5.36 kg/ha. The red parts in Figure 1 represent regions with high intensity of fertilizer loss and the pink parts represent are for regions with low intensity of fertilizer loss.

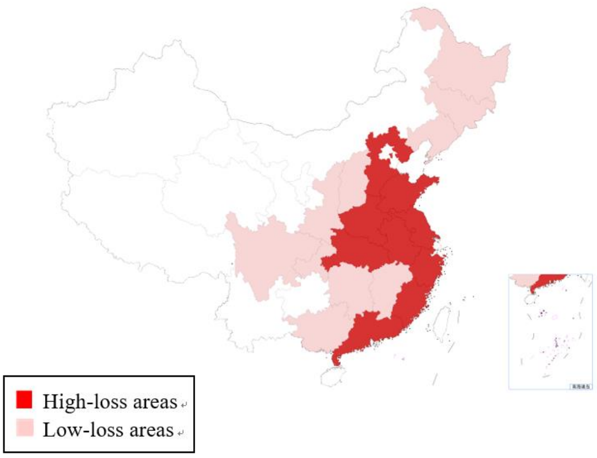

Fig 1 Fertilizer Loss in China

4. Line 105 What are other regional variables?
Response: Thank you very much for your suggestion. The other regional variables contain fertilizer use, organic fertilizer use, pesticide use, industrial pollution index, numbers of hospital and provincial Gross Domestic Product. GDP measures general economy and wealth conditions. The number of hospital controls for the level of medical service. Industrial pollutions control for pollution from industrial activities. All the regional variables are described in section “Provincial variables” in Section 2.1.3 “Provincial variables”.

5. Line 152 Why use the average of the past three years instead of using data from the same year? Is there a possibility of lagged effects?
Response: The consideration is as following. First, The individual health data from the Chinese Longitudinal Healthy Longevity Survey. We observed were once every three years. Second, as mentioned in our article, most studies indicated a lagged effects of environmental exposure on public health(e.g. Wang and Luo 2021; Hu et al. 2015) Therefore, we follow the literature and also use the average of the past three year considering the lagged effect as well as the consistency of health data.

6. Line 155 I suggest adding min. and max to the table.
Response: Thank you for the advice. We have added the min. and max in Table1.

7. Table 1 Provincial variables. Would there be some city-level factors omitted such as city-level subsidies that will influence both fertilizer application and human health?
Response: Thank you very much for your suggestion. This study explores the health effect of fertilizer loss at the provincial level and we have controlled the individual effects, so we did not control the city-level factors. We did not control the city-level subsidies because the samples are rural residents, not only the farmers who receive the health damage via direct exposure. We hold the fertilizer loss is exogenous.

8. Line 165 “Lines 1 and 2”, do you mean rows?
Response: Thank you for the suggestion. “Line1 and 2” means the rows of ADL index and No. of diseases, respectively. “Rows or Lines” maybe nor clearly enough, so we reinterpret the

results of Table 2 in Section 2.2.2.

9. Line 171 What are the average values of fertilizer loss?

Response: The fertilizer loss is continuous variable, and we dichotomized fertilizer loss by its pre-policy mean to compare the health effect in different regions. The average value of fertilizer loss is the national average of fertilizer loss; it is equal to 5.36kg/ha. We explained the details in section "Supporting information".

10. Line 165-Line 176 Please give more background information about the agricultural subsidy policy. What it is about and how does it impact fertilizer loss?

Response: Thank you for the suggestion. We have added the background about agricultural subsidy policy and the effect of fertilizer loss on environment and public health in Section 2.2.2.

11. Could you have a graph showing how the intensity changes over the years for both the high areas and low areas?

Response: Thank you for the suggestion. The fertilizer loss intensity and intensity variations are shown in Fig 2. Fertilizer loss increase per hectare in high loss regions is roughly 2times larger than low loss areas. The variations of fertilizer input intensity, fertilizer loss and fertilizer loss intensity were showed in Table2 in the revised manuscript.

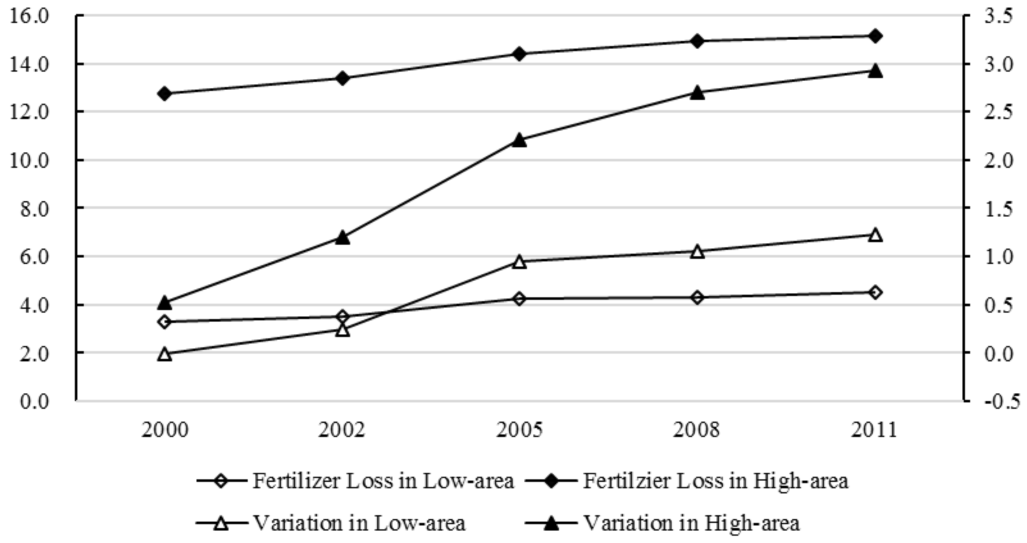

Fig 2 Fertilizer Loss Amount and Variation

12. This comparison of high and low is not exactly a difference-in-differences comparison because they both have the policy in place, and they are both "treated". This is more a two-way fixed effects methodology.

Response: Thank you for the suggestion. As you pointed out, all the samples are treated by agricultural support policies. A difference-in-differences is still applicable, but with two conditions need to be satisfied. First, the impacts are different between "experience group" and "control group". Second, the parallel trend assumption is need be hold. Following publications adopted DID to explain the "policy effect", however all samples were treated by the "policy": the bill of strengthening the protection of mortgage claims will have great

impact on enterprises with high proportion of tangible assets, because the tangible assets are easier to be used as collateral, so Vig(2013) constructed the experimental group and the control group according to the proportion of tangible assets of enterprises. Campello &Larrain (2016) constructed the experimental group and control group according to the difference of enterprises' demand for movable property. In our study, we constructed the experimental group and control group by fertilizer loss levels. First, the parallel trend assumption holds. Second, most of the high loss areas are in the developed regions and is the primary rice production regions in China. Agricultural subsidies of per unit of field are different between provinces, such as the subsidies in Jilin province is four and two times lower than Guangdong and Zhejiang respectively (Shu et al.,2013). The developed provinces had a larger subsides, and this will cause a larger fertilizer loss after agricultural support policies. The Green Revolution lead to the extensive use of chemical fertilizer, particular in rice production. We dichotomized fertilizer loss by its pre-policy mean. The variation of fertilizer loss in high loss areas is 2 times larger than low loss areas. Also, we used a continuous measure of intensity of treatment (i.e. fertilizer loss) and thereby captured more variation in the data (Nunn and Qian, 2011; Lai, 2017). As your suggestion, we have estimate the health effect of fertilizer loss using TWFE, and we have added the estimated results in column (3) and column (6) of Table3.

13. Line 179 Table notes should be self-explanatory. The readers could understand without referring too much to the paper.
Response: Thank you for your advice. We have deleted the table notes.

14. Equation (4) There is no subscript for X. Will heavy metals from organic fertilizer also impact human health? Will that bias your estimates?
Response: Thank you for the suggestion. We have added the subscript for X in Equation (4). Chemical fertilizer was most widely used in China and the supply proportion of chemical fertilizer nutrients exceeds seventy percent. The use of organic fertilizer was far less than that of chemical fertilizer, and the organic fertilizer use was concentrated in some regions. So we did not consider the health effect of organic fertilizer. But following your suggestion, we have controlled the organic fertilizer use in our revised manuscript.

15. Line 216 How old is defined as rural elderly and "younger elderly"? Which part of the results is based on equation (4) and which is from equation (5)?
Response: Thank you for the suggestion. According to the previous studies, an elderly person over 80 years or 85 years is defined as the oldest olds. The oldest olds were wildly discussed because they had greater risk of falling and hospitalization (Rockwood et al. 1999; Speechley &Tinetti, 1991; Winograd,1991; Brettschneider et al. 2019). We divided the samples by age into the younger elderly samples (<80years) and the oldest olds samples (≥80 years). The α_1 coefficient of equation (4) represented the post-agricultural policies of fertilizer loss. health effect of fertilizer loss and the β_1 of equation (5) captured the post-agricultural policies effect of high loss areas relative to low loss areas. Estimated coefficients of α_1 were reported in column (1) and column (3) of Table3, and estimated coefficients of β_1 were reported in column (2) and column (4) of Table3. We described the result of Table 3 in section 3.1.1.

16. Line 232 Policy implications can be put in the conclusion section.

Response: Thank you for this advice. We have deleted the policy implications Line229-235, Line261-257 of original manuscript. And we discussed the policy implications in conclusion section.

17. Table 3 What are the coefficients of the covariates (control variables)? Do they show the expected sign? What are the results without control variables?
Response: We have added the results without control variables in S1 Table in section "Support information" in our revise manuscript. The results with covariates are shown in Table 1 below.

Table 1 Basic Estimates ADL index and Diseases

	ADL		No. of diseases	
	(1)	(3)	(2)	(4)
Fertilizerloss*Time	0.0147**		0.0057***	
	(0.0061)		(0.0014)	
Higharea*Time		0.1222***		0.0487***
		(0.0474)		(0.0116)
Floss	0.0570	0.0659	-0.0148	-0.0124
	(0.0417)	(0.0416)	(0.0103)	(0.0103)
Pinpur	-94.2811	-125.0235	-47.6648	-65.0932**
	(116.5155)	(118.8320)	(30.3544)	(31.1875)
OFinput	0.0010	0.0010	0.0009***	0.0009***
	(0.0010)	(0.0010)	(0.0002)	(0.0002)
Age	0.0273	0.0323	0.0113	0.0109
	(0.0318)	(0.0317)	(0.0081)	(0.0081)
Male	-0.0103	-0.0373	-0.0029	-0.0042
	(0.2373)	(0.2385)	(0.0426)	(0.0427)
Co-residence	0.1617***	0.1647***	-0.0074	-0.0077
	(0.0256)	(0.0256)	(0.0066)	(0.0066)
Income_cost	0.0354*	0.0271	-0.0024	-0.0023
	(0.0200)	(0.0199)	(0.0046)	(0.0046)
Illness	1.5128***	1.4911***	3.5079***	3.5068***
	(0.1930)	(0.1923)	(0.0580)	(0.0581)
Hospitalization	0.1171***	0.1170***	0.0126***	0.0126***
	(0.0183)	(0.0183)	(0.0038)	(0.0038)
Smoke	-0.0784	-0.0722	0.0369*	0.0366*
	(0.0856)	(0.0851)	(0.0201)	(0.0201)
Drink	-0.0330	-0.0264	-0.0104	-0.0103
	(0.0663)	(0.0662)	(0.0167)	(0.0167)
Exercise	-0.2192***	-0.2181***	-0.0123	-0.0123
	(0.0489)	(0.0488)	(0.0127)	(0.0127)
Meat	0.0196	0.0044	0.0056	0.0054
	(0.0140)	(0.0184)	(0.0044)	(0.0044)
Fish	0.0245*	0.0132	-0.0014	-0.0015
	(0.0131)	(0.0184)	(0.0045)	(0.0045)
Egg	-0.0140	-0.0247	0.0024	0.0025

	(0.0116)	(0.0183)	(0.0046)	(0.0046)
Salt_veg	-0.0435***	-0.0406***	-0.0075***	-0.0075***
	(0.0089)	(0.0089)	(0.0024)	(0.0024)
Boild water	-0.0836*	-0.0934**	-0.0042	-0.0050
	(0.0456)	(0.0457)	(0.0107)	(0.0107)
Water	0.0028	0.0027	-0.0152	-0.0147
	(0.0823)	(0.0822)	(0.0175)	(0.0175)
Hospnum	0.0712***	0.0706***	-0.0155***	-0.0159***
	(0.0153)	(0.0154)	(0.0041)	(0.0041)
LnGDP	-0.2306	-0.2379	0.0920	0.0996*
	(0.2207)	(0.2185)	(0.0585)	(0.0576)
Indpolltion	0.0662	0.0495	0.0291	0.0279
	(0.1085)	(0.1081)	(0.0253)	(0.0253)
Constant	4.8844	5.0608	-1.6139*	-1.6166*
	(3.3262)	(3.3242)	(0.8570)	(0.8543)
Observations	32,165	32,165	32,329	32,329
R-squared	0.5570	0.5582	0.6906	0.6907
Year FE	YES	YES	YES	YES
ID FE	YES	YES	YES	YES

robust standard errors for clustering at the individual level are reported in brackets; *, **, *** are significant at the level of 1%, 5% and 10% respectively

18. Line 248 What do you mean by "reduces the age of the elderly"? Please pay attention to how to interpret the results.
Response: Thank you for the advice. "Reduces the age of elderly" means the fertilizer loss will lead the age of illness onset younger. As your suggestions, we have deleted this confusing expression and interpreted the results of Table 4 in section 3.1.3.

19. Line 276 How are the costs of fertilizer application compared to the cost of this pesticide use?
Response: Thank you for the suggestion. This study focused on the health effect and medical cost loss of fertilizer. We have estimated the medical cost of fertilizer loss. An increase of one unit (1kg/ha) of fertilizer loss would add CNY 3.74 (USD 0.53) to medical cost, which would increase 316 million yuan (45 million dollars) in total for the rural elderly populations in China. For you advise, we added medical cost of fertilizer input in Line290-293. One more unit fertilizer input will increase medical cost by CNY 0.22. For the elderly population in rural China, the total medical cost will increase CNY 21.5 (USD 3.1) million.

20. Line 301 Do you mean equations (4) and (5)?
Response: Sorry about these errors. We have corrected these errors.

21. Line 317 What are these rice-producing areas. Could you be more specific?

Response: Thank you for the suggestion. The details about major agricultural-producing areas, major rice-producing areas and water resource-rich areas are in section supporting information.

22. Formatting:
Introduction. Each Line ends with a half-word. I suggest checking the composition/distribution of paraphs.
Response: Thank you for this suggestion. We have revised it in introduction and other parts of our manuscript.

23. "This is table X legend." What is this for? Considering removing. Please also check the table adjustment and Line spacing.
Response: Thank you for the suggestion. *"This is table X legend"* is the template format. We have adjusted the single spacing of the tables.

24. Grammar mistakes and typos:
There are grammar mistakes and typos. Please also check the uses of some phrases. Examples are seen in the following, but not limited to:
First paragraph in the introduction, "stats, surmise"
Line 56 Plication
Line 100 "the data of 1998…."
Line 127 "rejuvenation of disease", "income station
Line 146 should this n be h?
Line 155 Indicaos should be indicators
Line 165 "after the implementation" duplicated phrases
Line 201 medic should be medical
Line 202 than should be then
Some sentences can be cleaned and simplified. I encourage the authors to have some native or fluent speakers read through and help with the language of the paper.
Response: Sorry for these errors. In our revise manuscript, we have carefully checked the grammar and revised the sentences.

Reviewer: 2

Thank you for the constructive comments on our manuscript. We have taken your comments seriously and substantially modified portions of the manuscript. We believe our work has been significantly improved as a result of your comments. Please find below our response to each point you made with your original comments in italic.

This paper focuses on an important and interesting topic of fertilizer exposure/application and human health. Although many efforts are put into this work, the paper still needs some improvement regarding methodology and organization/formatting of the paper. The writing and language should also be improved significantly before being published. At the beginning of the introduction, a paper usually starts with background information and introduces the issue addressed by the paper. The first paragraph is not clear and interesting enough by listing some findings.

General comments

1. Although it is important to examine the regional inequality in the medical outcome, the most important question is: why aged people? Previous studies show that fertilizer loss damages residents, then the natural steps should be who are more subjected to harm from fertilizer loss. Therefore, citing a study to show the difference between elder residents and younger residents is appreciated.

Response**：** Thank you for the suggestion. China has the most serious aging, 70% of elderly live in rural areas. Agricultural non-point pollutions had been more serious according to the overuse of agrochemicals. Under the background of aging and non-point pollution, this study focused on health effect of fertilizer loss on elderly in rural areas. The elder residents have different vulnerabilities to environmental contaminants. We have cited several studies in Line 44-48.

2. Therefore, hypothesis 1 needs transition. If fertilizer loss is harmful to health, in general, is known, why do we have to explore its effect on aged residents, before showing whether there is a difference between elder and younger residents? Are they more vulnerable or more resilient than the younger group? This problem can be solved by citing studies showing fertilizer overuse has a different effect on elder and younger people.

Response: Several studies had reported that the elder residents were more vulnerable than the younger groups. A higher susceptibility and higher mortality rates could be observed among the elder residents (We cited the studies in Introduction(Line44-45). We have added more references to explain the reasons of heterogeneity effect in younger and older population in Line 45-48. We further explored heterogeneous health effect of fertilizer loss by splitting the samples in various ages. First, we divided the samples at age into the younger age samples (<80years) and the oldest olds samples (≥80 years). Then, we also divided the samples into younger olds (65years-75years), middle olds (75-85years) and oldest olds (≥85years) according to Monti et al. 2021 and Lau et al. 2021. The heterogeneous effects are in section 3.1.2.

3. For hypothesis 2, you might want to add more explanation on how the outcome variables are correlated with the "rejuvenation of disease". How do you define the term?
Response: We have rewired the hypothesis 2. Because of the physical function, fertilizer loss may lead the samples to be ill younger. The fertilizer loss caused the several diseases, which in Line76-83. And in Figure 2, the elderly under 80 years of age, there was a significant difference in the age of illness onset in the areas with high chemical fertilizer loss, and the age of illness onset in the elderly in the areas with high chemical fertilizer loss was lower than that in the areas with low chemical fertilizer loss.

4. Hypothesis 3 makes two points, and should be separated; their contents are also made sense by common wisdom, but the literature in the manuscript seems not to correlate with them.
Response: Thank you for the suggestion. We revised the hypothesis 3 by "Due to the health damages, the medical cost will be higher in high-loss regions comparing the regions with lower fertilizer loss". In this section, we explored the economic results by translating estimated effects into expected monetary loss.

5. The linear regression coefficients are correlations, not "impact". The impact has a causal implication.
Response: Thank you for the suggestion. We have revised the expressions by using "lead to, be associated with".

6. This research uses DID, but the research did not check the parallel trends before 2004. In this study, the main analysis uses data after 2004 and separates the provinces into high-loss and low-loss. However, as far as I am aware, there is no mention of how the health outcomes trend before 2004. For DID, parallel trends assumption is essential. I randomly search for a blog from the world bank that could help the authors to have the idea (https://blogs.worldbank.org/impactevaluations/revisiting-difference-differences-parallel-trends-assumption-part-i-pre-trend). Your placebo test does not correlate with this problem.
Response: Thank you for the suggestion. We added the parallel trends in section 3.3.1. We divided the samples to six sections: the second period before the policy implementation, the first period before the policy implementation, the second period after policy, the second two periods after the policy implementation to the end of sample period. Thus, six indicator variables pre_2, pre_1, post_1, post_2, post_3 and post_4 were constructed. The set of pre_1 coefficient were statistically indistinguishable from zero, but there was a sharp, statistically significant and sustained change after the policy implementation.

7. Even if the parallel trend assumption holds, another problem is how to support the assumption that all the residents are treated to high fertilizer overuse randomly. High fertilizer loss could also imply a lack of management or facility, which also leads to high medical loss. For example, it can be argued that the aged person exposed to extra use of fertilizer tend to live in provinces with lower medical resources (agriculture province such as Hubei are also subjected to fertilizer overuse). In short, the author should consider blocking potential causal paths.
Response: Thank you for the suggestion. We had considered the randomization of fertilizer loss. A major concern was that the factors that caused the different fertilizer loss between

provinces may be correlated with health outcomes. These types of confounding factors vary across provinces and individuals but are fixed over time. A common method of controlling for time-invariant unobserved heterogeneity is to use fixed effects. We had fixed the individual effect and time effect. And we also had controlled the medical resources and economic developments.

8. Another problem that comes with DID is the treatment effect. In formula (4), it seems the treatment effect is continuous. However, the traditional DID does not allow for the continuous treatment effect and could miscalculate S.D and perform the wrong t-test. I suggest reading this paper and reconsidering this part:
Callaway, B., Goodman-Bacon, A., & Sant'Anna, P. H. C. (2021). Difference-in-Differences with a Continuous Treatment. ArXiv:2107.02637 [Econ]. http://arxiv.org/abs/2107.02637
Response: Thank you for this advice and thank you very much for recommending the paper. We have learned the paper you recommended carefully, the paper provided a good job in the front of theoretical basis research of Difference-in-Differences. Unfortunately to date a package that allows researchers to implement this approach in Stata has not been released. And sorry for that we are not capable of empirical research following Callaway's theory. However, if differences in treatment intensity are not correlated with heterogeneity in the treatment effect at a given treatment intensity or there is no selection of outcome variables, the traditional DiD can be used (Callagher, et al. 2021; Kindsgrab, 2022; Battiston et al.2021). Callaway argued that the magnitude of coefficient with continuous treatment from DiDs should be interpreted with caution as the interaction coefficient identifies a weighted average of the "average causal response" of the treatment along different levels of the treatment. In our setting, we are interested in causal parameter $ATT(d \mid d)$ the average treatment effect to fertilizer loss level d. When we want to compare the magnitudes of $ATT(d \mid d)$ across different levels of health damage (i.e. Equation4 and Equation6), we need a stronger parallel trends assumption that the restricts the potential outcomes of individuals. Specifically, we must assume that the average potential outcomes for individuals are the same at each level of fertilizer loss, or that (on average) there is no selection into a particular level of fertilizer loss. The stronger parallel trends assumption is likely to be satisfied in our setting. In addition, we followed some literatures by adopting a binary treatment as robustness check (Gong et al.2022; Motohashi,2022).

Minor problems are listed as follows:
9. Replacing the word "rejuvenation of disease". Many definitions can be found on the internet, so it might be helpful to address this word.
Response: Thank you for thesuggestion. In this study, "rejuvenation of disease" means that the residences may be ill in younger ages. We have changed the expression by "be ill younger" and "be sick in younger age".

10. The research can be more specific on the unit of analysis. "Matches micro-level individual health data and provincial-level chemical fertilizer" is confusing.
Response: Thank you for the advice. The analysis was accomplished using difference in differences method with the national dataset Chinese Longitudinal Healthy Longevity Survey (CLHLS) and publicly published yearbooks. We have revised the explanation in first paragraph of Section 2.1 "Data Collection and Variables". We have introduced the CLHLS

database and how did the individual variables defined in section 2.1.1 and section 2.1.2. We also explained the provincial variables in section 2.1.3.

11. No need to discuss policy implications in basic regression results of health. I was referring to the line(L)229-L235, L261-257,
Response: Thank you for the suggestion. We have deleted the discussion of policy implications.

12. When you use "1% level is significant", do you mean "significant at 99% CI"?
Response: Thank you for the suggestion. Yes, "1% level is significant" means "significant at 99% CI". "1% level is significant" is usually used in economics.

13. L316-L319: Are you referring to table 6 or table 7?
Response: Sorry for these errors. Line316-319 is the descriptions of Table 7. And we have moved Table7 to section "Supporting information", in S3 Table and S4 Table.

14. Results of the placebo test, the Effect of fertilizer input on health, and the Effect in different areas can be moved to the appendix.
Response: Thank you for the suggestion. We have moved the robustness check to the Supporting information.

To sum up, in its current form, this manuscript is not ready to publish. However, if the author is willing to address these mentioned problems, then the topic is interesting and contributes to the existing literature.

Reference

1. Battiston D, Espinosa M, Liu S. Talent poaching and job rotation. SSRN. 2021; 3778068,
2. Brettschneider C, Hajek A, Rohr S, Fuchs A, Weeg D, et al. Determinants of health-care costs in the oldest-old in Germany. The Journal of the Economics of Ageing. 2019; 14: 100200.
3. Campello M, Larrain M. Enlarging the contracting space: Collateral menus, access to credit, and economic activity. The Review of Financial Studies. 2016; 29(2): 349-383.
4. Gallagher J, Hartley D, Rohlin S. Weathering an unexpected financial shock: the role of federal disaster assistance on household finance and business survival. 2021.
5. Gong D, Yan A, Yu J. Cost of zero-covid: effects of anti-contagious policy on labor market outcomes in china[J]. SSRN.2022; 4037688.
6. He GJ, Liu T, Zhou MG. Straw burning, PM 2.5, and death: evidence from China. Journal of Development Economics. 2020;145: 102468.
7. Hu R, Huang X, Huang J, Li Y, Zhang C, et al. Long- and short-term health effects of

pesticide exposure: a cohort study from China. PLOS One. 2015; 10(6): e0128766.
8. Kindsgrab P. Do higher income taxes on top earners trickle down? A local labor markets approach. A Local Labor Markets Approach, 2022.
9. Lai WY. Pesticide use and health outcomes: evidence from agricultural water pollution in China. Journal of Environmental Economics and Management. 2017; 86: 93-120.
10. Motohashi K. Unintended consequences of sanitation: externalities on water quality and health in India.2022.
11. Nunn N, Qian N, The potato's contribution to population and urbanization: evidence from a historical experiment. The Quarterly Journal of Economics. 2011;126(2): 593-650.
12. Rockwood K, Stadnyk K, MacKnight C, McDowell I. A brief clinical instrument to classify frailty in elderly people. The Lancet. 1999; 353:205-206.
13. Shu KL, Yang S, Wang HL, Guo QH. Analysis on evolution of Jilin grain production investment mechanism and its relevant policy effect. Research of Agricultural Modernization. 2013;34(03): 257-262.
14. Speechley M, Tinetti M. Falls and injuries in frail and vigorous community elderly persons. Journal of the American Geriatrics Society. 1991; 39:46-52.
15. Wang Y Z, Luo N S. Air pollution, health depreciation and medical costs: Research based on the three perspectives of physical health, mental health and social adaptability[J]. Economic Research Journal, 2020, 55(12): 80-97.
16. Winograd CH. Targeting Strategies: An overview of criteria and outcomes. Journal of the American Geriatrics Society. 1991; 39:25-35.
17. Vig V. Access to collateral and corporate debt structure: evidence from a natural experiment. The Journal of Finance. 2013; 68(3): 881-928.
