# Supplementary material for: Agricultural non-point source pollution and health of the elderly in rural China
Source: PLoS One. 2022 Oct 14;17(10):e0274027. doi: 10.1371/journal.pone.0274027 (PMC9565375; doi:10.1371/journal.pone.0274027)
Supplement: Attachment — Submitted filename: PONE-D-21-34489_R2_0723.pdf [file pone.0274027.s009.pdf]

PLOS ONE

Agricultural Nonpoint-source Pollution and Health of the Elderly in Rural China

--Manuscript Draft--

Manuscript Number:	PONE-D-21-34489R2
Article Type:	Research Article
Full Title:	Agricultural Nonpoint-source Pollution and Health of the Elderly in Rural China
Short Title:	none short title
Corresponding Author:	YING WANG<br>Nanjing Agricultural University College of Economics and Management<br>Nanjing, CHINA
Keywords:	chemical fertilizer loss, aging, health risk, difference-in-differences
Abstract:	Large input and high loss of chemical fertilizer are the main causes of agricultural nonpoint-source pollution in China. Employing fertilizer loss and micro-health data, this paper analyzes the effects of chemical fertilizer loss on the health of rural elderly and the medical cost in China. Results of the difference-in-differences (DID) method indicate that one kg/ha increase in fertilizer loss alters a key medical disability index (Activities of Daily Living) by 0.0147(0.2 percent changes) and the number of diseases by 0.0057 for rural residents of 65 and older. This is equivalent to CNY 316 million (USD 45 million) in national medical cost. Furthermore, the age of onset is younger in regions with higher fertilizer loss. One kg/ha increase of fertilizer loss advances the age of onset by 0.267 year, which will cause long term effect on public health. Our results are robust to a variety of robustness checks.
Order of Authors:	Ying WANG
	Hang XIONG
	Chao CHEN
Response to Reviewers:	Response to Editor<br>Dear Dr. Qiu,<br><br>Thank you for coordinating, reviewing, and extending the invitation to revise our manuscript. We have treated yours as well as the reviewers' comments seriously and made significant modification to the manuscript. As follows, we will summarize revisions implemented following the two reviewers' comments before point by point responses.<br><br>For Reviewer1, we have:<br>1.Improved writing and readability by proving a better expression and revising Figure 1 and Figure 2.<br>2.Added the regression results of percentage changes and the details of TWFE model.<br><br>For Reviewer2, we have:<br>1.Revised the section of "parallel trend analysis" and explained the regression results in more details.<br>2.Added the regression results of propensity score matching (PSM) difference-in-differences (DID).<br>3.Offered the binary robustness check by changing the cutoff for the binary treatment effect.<br><br>We hope you find our revised manuscript acceptable for publication in PLOS ONE.<br><br>Sincerely,<br><br>Ying Wang<br><br>Cc. Hang Xiong and Chao Chen<br><br>Reviewer: 1

Powered by Editorial Manager® and ProduXion Manager® from Aries Systems Corporation

Thank you for the constructive comments on our manuscript. We have taken your comments seriously and substantially modified the manuscript. We believe our work has been significantly improved thanks to your comments. Please find below our response to each point you made with your original comments in italic.

The manuscript is much improved. I have the following comments before this paper can be published. I have also highlighted some minor comments such as grammar mistakes, typos, and formatting errors in the revised manuscript (see pdf if applicable).
1.Abstract: "ADL daily life index of 0.0147.." How much is this change in percentage?
Response：Thank you very much for your question. ADL daily index of 0.0147 is 0.2 percent change. We have added the percentages in the abstract. The empirical model results are in Table1. The explained variables are the logarithmic of health outcome variables.
Table 1 Empirical result of the logarithmic of health outcome variables.
ADLNo. of diseases
(1)(2)(3)(4)
Fertilizerloss*Time0.0020***0.0017**0.00240.0047*
(0.0006)(0.0007)(0.0030)(0.0028)
ControlNOYESNOYES
Year FEYESYESYESYES
ID FEYESYESYESYES
Observations321893216544264395
R-squared0.56180.57190.52450.6093
Notes:
Robust standard errors for clustering at the individual level are reported in brackets.
*, **, *** are significant at the level of 10%, 5% and 1% respectively.

2.Figure 1: Can you add something about the pathways, such as through drinking water?
Response：Following your advice, we have revised the Figure 1.

3.I did not see a figure showing the Fertilizer loss in China (maybe you can put it into the Supporting information)
Response：Thank you very much for your suggestion. We have added S1 figure to demonstrate the Fertilizer Input and Fertilizer Loss in China in the Supporting information.

4.Figure 2: Could you add a vertical line to show treatment time?
Response：Following your advice, we have added a vertical line to show treatment line and we have replaced the figure2.

5.Table 3 Missing variable name for coefficients. Please add. Also, could you check the R2 for column (3)?
Response：Sorry about these errors. In our revised manuscript, we have carefully corrects these errors.

6.What are the model specifications for TWFE?
Response：Thanks for your question. We use two-way fixed effect model to estimate the health effect of fertilizer loss directly. We fix the individual and time effects both.
(1)
where demotes the health outcome; identifies the marginal effect of fertilizer loss; is a vector of control variables including individual, household, and provincial characteristics such as age, gender, health behavior, dietary habits, industrial pollution and hospitals, etc.; is a fixed effect unique to individuals ; is a time effect common to all individuals in year ; and is an error term.

7.Table 4 Do individual fixed effects mean the individual person (survey participants) fixed effects or province fixed effects?

	Response：In this paper, individual fixed effects mean the individual person (survey participants) fixed effects. And we have revised by “ID FE” to make the expression clearer.<br><br>8.Section 3.2.1 “The differences between younger and middle olds are not significant in both ADL and No. of diseases”. Do you expect that the effects are homogenous across ages? Are there any possible explanations if they are the same?<br>Response：Thank you for the questions. The health effects of pollution expose are complex and does not follow a simple linear or near-linear relationship. The elderly often has a higher susceptibility and a higher mortality rate (Wong et al., 2015; Cohen and Gerber, 2017). The heterogeneous effects are because of the age-related behaviors and the physical functions (Tuttle et al., 2013; Rockwood et al., 1999). Increasing variability in diseases and disabilities could be found when people aged and the aging people were more likely to fall and be hospitalized (Speechley and Tinetti, 1991; Winograd, 1991). The younger elderly had better physical functions, so effects on ADL and No. of diseases are not significant. However, the younger elderly also has health risks. The elderly becomes suffered from the diseases related to fertilizer loss as the age growing. So a significant effect on No. of diseases could be observed among the middle olds elderly.<br><br>9.Table 5 what are the p values?<br>Response：P-values estimate the likelihood of obtaining the observed differences in coefficient estimates. The original hypothesis is there is no difference between groups ( ).The model results reject the original hypothesis. It means that there is a significant difference between groups.<br><br>10.Section 3.3.1 Parallel trend test Why are they divided into five periods?<br>Response：Thank you very much for your question. There are six waves (2000, 2002, 2005, 2008, 2011, 2014) survey data. Two periods before the agricultural support policies, and four periods after treatment time. We choose the first period before the treatment time point as the benchmark group, so there are five periods. We have restructured this part.<br><br>11.Table S3 What do that superscripts for p values mean?<br>Response：P-values estimate the likelihood of obtaining the observed differences in coefficient estimates.<br><br>Response for comments in the PDF documents:<br><br>1.Thank you very much for the suggestion about typographical and grammatical errors. We have corrected the errors. And we have checked publications in the journal and reconfirmed that the supporting information citations as “S1 Fig” and “S1 Table”.<br><br>2.We have revised the section of conclusions and discussion and shortened the expression of Line344-347.<br><br>3.For response 9: We have added the details about the average values of fertilizer loss in note of Table 2 in our revised manuscript.<br><br>4.For response 17: There is a double-direction causality relationship between medical supply and health. And the medical supply is only a control variable, so we did not discuss the double-direction causality relationship. Further, we estimated health effect of fertilizer loss by deleting the medial supply variable (the results in Table 2). Also the negative effects between medical supply and health were found in other literatures (Lai, 2017; Ju,2022), when they controlled medical supply.<br>Table 2 Estimates without variable of medical supply

Powered by Editorial Manager® and ProduXion Manager® from Aries Systems Corporation

ADLNo. of diseases
Fertilizerloss*Time0.0173***0.0051***
(0.0061)(0.0014)
ControlYESYES
Year FEYESYES
ID FEYESYES
Observations3216532329
R-squared0.55640.6903
Notes:
*, **, *** are significant at the level of 10%, 5% and 1% respectively.

Reviewer: 2
6. Your parallel trend analysis is appreciated. However, you might want to add an explanation on which figures are for low fertilizer regions and which are for the high fertilizer regions. Otherwise, I cannot understand. Also, the explanation "We divide the samples to five sections" is also confusing. You could consider rewriting this part.
Response：Thank you very much for your suggestion. In order to test whether the time staggered entry events of agricultural policy are effective exogenous shocks, we conduct a parallel test base on Equation (9):
(9)
where is a binary variable that takes value of 1 when agricultural support policies are implemented. We use to denote the base year. Panel A and Panel B of S2 Fig were the estimate results of fertilizer loss and Panel C and Panel showed the estimated coefficients when adopting a binary variable that takes values 1 as high loss areas. Following your advice, we have restructured the Section of Parallel trend test. We added the Equation (9) to explain how we do the parallel trend, and we explained S2 Fig in more detail.

7. I am concerned about this treatment. Although in some cases, adding linear coefficients could work, health factors are sometimes not linear. Do the authors have considered matching? If so, how does it go?
Response：Following your advice, three matching techniques are utilized to assess the health effect of fertilizer loss, namely nearest neighbor matching, the radius matching and the kernel matching. The matching results are reported in Table 3. The estimated coefficients are significant and it means that the basic results are robust. We have added the Table 3 in support information as S2 Table in our revised manuscript.
Table 3 Estimates of Matching
Nearest neighborRadiusKernel
ADLNo. of diseases ADLNo. of diseases ADLNo. of diseases
Fertilizerloss*Time0.0695**0.0143**0.0267***0.0065***0.0267***0.0065***
(0.0273)(0.0064)(0.0085)(0.0023)(0.0085)(0.0023)
ControlYESYESYESYESYESYES
Year FEYESYESYESYESYESYES
ID FEYESYESYESYESYESYES
Observations453245321780117801178011780 1
R-squared0.59270.71520.58280.70490.58280.7049
a *, **, *** are significant at the level of 10%, 5% and 1% respectively.

8. I know it makes our life difficult if no package is available. However, assuming the level of fertilizer loss does not have different impacts is too strong. After all, this is the

main argument of this study. I would suggest, that given the authors already adopt binary treatment in the robustness check, the authors should repeat the binary robustness check by calibrating the cutoff for the binary treatment effect.
Response：Following your advice, we have offered the binary robustness check by changing the cutoff for the binary treatment effect. We defined the high loss areas with the top 10%, 20%, 30% and 40% of the highest fertilizer loss intensity respectively. Table 4 reports the estimate coefficients in Equation (5), the basic regression results of binary treatment are robust to a variety of robustness checks.

Table 4 The Robustness Checks of Binary Treatment
ADLNo. of diseases
10%20%30%40%10%20%30%40%
Higharea*Time0.1193**0.1278**0.1946***0.1890***0.0239*0.0621***0.0500***0.0671***
(0.0596)(0.0525)(0.0500)(0.0491)(0.0142)(0.0122)(0.0119)(0.0120)
ControlYESYESYESYESYESYESYESYES
Year FEYESYESYESYESYESYESYESYES
ID FEYESYESYESYESYESYESYESYES
Observations32,18932,18932,18932,18932,35332,35332,35332,353
R-squared0.56200.56210.56230.56230.68980.69010.69000.6902
a robust standard error for clustering at the individual level are reported in brackets; *, **, *** are significant at the level of 10%, 5% and 1% respectively

Reference

1.Wong CM, Lai HK, Tsang H, et al. Satellite-based estimates of long-term exposure to fine particles and association with mortality in elderly Hong Kong residents. Environmental Health Perspectives. 2015;123(11):1167-1172
2.Cohen, G., Gerber, Y. Air pollution and successful aging: Recent evidence and new perspectives. Current Environmental Health Reports. 2017; 4:1-11. https://doi.org/10.1007/s40572-017-0127-2. PMID: 28101729.
3.Tuttle L, Meng Q, Moya J, Johns DO. Consideration of age-related changes in behavior trends in older adults in assessing risks of environmental exposures. Journal of Aging and Health. 2013; 25(2):243-273.
4.Rockwood K., Stadnyk K., MacKnight C., et al. A Brief Clinical Instrument to Classify Frailty in Elderly People. The Lancet. 1999; 353: 205-206.
5.Speechley M. and Tinetti M. Falls and Injuries in Frail and Vigorous Community Elderly Persons. Journal of the American Geriatrics Society.1991; 39: 46-52.
6.Winograd C.H. Targeting Strategies：An Overview of Criteria and Outcomes. Journal of the American Geriatrics Society. 1991; 39: 25S-35S.
7.Lai WY. Pesticide use and health outcomes: evidence from agricultural water pollution in China. Journal of Environmental Economics and Management. 2017; 86: 93-120.
8.Ju K, Lu LY, Chen T, et al. Does long-term exposure to air pollution impair physical and mental health in the middle-aged and older adults? — A causal empirical analysis based on a longitudinal nationwide cohort in China. Science of The Total Environment. 2022; 87: 154312.

Additional Information:	
**Question**	**Response**
**Financial Disclosure**<br><br>Enter a financial disclosure statement that describes the sources of funding for the work included in this submission. Review the submission guidelines for detailed requirements. View published research articles from *PLOS ONE* for specific examples.<br><br>This statement is required for submission and **will appear in the published article** if	This article was sponsored by the Priority Academic Program Development of Jiangsu Higher Education Institutions (PAPD). The funders had no role in study design, data collection and analysis, decision to publish, or preparation of the manuscript.

Powered by Editorial Manager® and ProduXion Manager® from Aries Systems Corporation

the submission is accepted. Please make sure it is accurate.<br><br>**Unfunded studies**<br>Enter: *The author(s) received no specific funding for this work.*<br><br>**Funded studies**<br>Enter a statement with the following details:<br>• Initials of the authors who received each award<br>• Grant numbers awarded to each author<br>• The full name of each funder<br>• URL of each funder website<br>• Did the sponsors or funders play any role in the study design, data collection and analysis, decision to publish, or preparation of the manuscript?<br>• **NO** - Include this sentence at the end of your statement: *The funders had no role in study design, data collection and analysis, decision to publish, or preparation of the manuscript.*<br>• **YES** - Specify the role(s) played.<br><br>* typeset	
**Competing Interests**<br><br>Use the instructions below to enter a competing interest statement for this submission. On behalf of all authors, disclose any competing interests that could be perceived to bias this work—acknowledging all financial support and any other relevant financial or non-financial competing interests.<br><br>This statement is required for submission and **will appear in the published article** if the submission is accepted. Please make sure it is accurate and that any funding sources listed in your Funding Information later in the submission form are also declared in your Financial Disclosure statement.<br><br>View published research articles from *PLOS ONE* for specific examples.	The authors have declared that no competing interests exist.

**NO authors have competing interests**<br><br>Enter: *The authors have declared that no competing interests exist.*<br><br>**Authors with competing interests**<br><br>Enter competing interest details beginning with this statement:<br><br>*I have read the journal's policy and the authors of this manuscript have the following competing interests: [insert competing interests here]*<br><br>* typeset	
**Ethics Statement**<br><br>Enter an ethics statement for this submission. This statement is required if the study involved:<br><br>• Human participants<br>• Human specimens or tissue<br>• Vertebrate animals or cephalopods<br>• Vertebrate embryos or tissues<br>• Field research<br><br>Write "N/A" if the submission does not require an ethics statement.<br><br>General guidance is provided below. Consult the submission guidelines for detailed instructions. **Make sure that all information entered here is included in the Methods section of the manuscript.**	N/A. The individual informations come from a national survey. The survey is about the elderly in 22 provinces of China. The respindents are willing to accept the survey, and the survey data can be used pubicly. The private information that can identify the respondents are hidden.

**Format for specific study types**<br><br>**Human Subject Research (involving human participants and/or tissue)**<br>• Give the name of the institutional review board or ethics committee that approved the study<br>• Include the approval number and/or a statement indicating approval of this research<br>• Indicate the form of consent obtained (written/oral) or the reason that consent was not obtained (e.g. the data were analyzed anonymously)<br><br>**Animal Research (involving vertebrate animals, embryos or tissues)**<br>• Provide the name of the Institutional Animal Care and Use Committee (IACUC) or other relevant ethics board that reviewed the study protocol, and indicate whether they approved this research or granted a formal waiver of ethical approval<br>• Include an approval number if one was obtained<br>• If the study involved *non-human primates*, add *additional details* about animal welfare and steps taken to ameliorate suffering<br>• If anesthesia, euthanasia, or any kind of animal sacrifice is part of the study, include briefly which substances and/or methods were applied<br><br>**Field Research**<br><br>Include the following details if this study involves the collection of plant, animal, or other materials from a natural setting:<br>• Field permit number<br>• Name of the institution or relevant body that granted permission	
**Data Availability**<br><br>Authors are required to make all data underlying the findings described fully available, without restriction, and from the time of publication. PLOS allows rare exceptions to address legal and ethical concerns. See the PLOS Data Policy and FAQ for detailed information.	Yes - all data are fully available without restriction

Powered by Editorial Manager® and ProduXion Manager® from Aries Systems Corporation

A Data Availability Statement describing where the data can be found is required at submission. Your answers to this question constitute the Data Availability Statement and **will be published in the article**, if accepted.<br><br>**Important:** Stating 'data available on request from the author' is not sufficient. If your data are only available upon request, select 'No' for the first question and explain your exceptional situation in the text box.<br><br>Do the authors confirm that all data underlying the findings described in their manuscript are fully available without restriction?	
**Describe where the data may be found in full sentences. If you are copying our sample text, replace any instances of XXX with the appropriate details.**<br><br>• If the data are **held or will be held in a public repository**, include URLs, accession numbers or DOIs. If this information will only be available after acceptance, indicate this by ticking the box below. For example: *All XXX files are available from the XXX database (accession number(s) XXX, XXX.*).<br>• If the data are all contained **within the manuscript and/or Supporting Information files**, enter the following: *All relevant data are within the manuscript and its Supporting Information files.*<br>• If neither of these applies but you are able to provide **details of access elsewhere**, with or without limitations, please do so. For example:<br><br>*Data cannot be shared publicly because of [XXX]. Data are available from the XXX Institutional Data Access / Ethics Committee (contact via XXX) for researchers who meet the criteria for access to confidential data.*<br><br>*The data underlying the results presented in the study are available from (include the name of the third party*	The data underlying the results presented in the study are available from https://opendata.pku.edu.cn/dataverse/CHADS;jsessionid=6a16c8c5d8c20925c51b93da42fc.

and contact information or URL).<br>• This text is appropriate if the data are owned by a third party and authors do not have permission to share the data.<br><br>* typeset	
Additional data availability information:	

Manuscript Click here to access/download;Manuscript;Manuscript.docx

Agricultural Nonpoint-source Pollution and Health of the Elderly in Rural China

Ying WANG[1], Hang XIONG[2], Chao CHEN[3*]

[1] College of Economics and Management, Nanjing Agricultural University, Nanjing, Jiangsu, P.R.China

[2] College of Economics and Management, Nanjing Agricultural University, Nanjing, Jiangsu, P.R.China; China Center for Food Security Studies, Nanjing Agricultural University, Nanjing, Jiangsu, P.R.China

[3] College of Economics and Management, Nanjing Agricultural University, Nanjing, Jiangsu, P.R.China; China Center for Food Security Studies, Nanjing Agricultural University, Nanjing, Jiangsu, P.R.China

* Corresponding author

E-mail: cchen@njau.edu.cn.

Funding Information：This article was sponsored by the Priority Academic Program Development of Jiangsu Higher Education Institutions (PAPD)

Abstract

Large input and high loss of chemical fertilizer are the main causes of agricultural nonpoint-source pollution in China. Employing fertilizer loss and micro-health data, this paper analyzes the effects of chemical fertilizer loss on the health of rural elderly and the medical cost in China. Results of the difference-in-differences (DID) method indicate that one kg/ha increase in fertilizer loss alters a key medical disability index (Activities of Daily Living) by 0.0147(0.2 percent changes) and the number of diseases by 0.0057 for rural residents of 65 and older. This is equivalent to CNY 316 million (USD 45 million) in national medical cost. Furthermore, the age of onset is younger in regions with higher fertilizer loss. One kg/ha increase of fertilizer loss advances the age of onset by 0.267 year, which will cause long term effect on public health. Our results are robust to a variety of robustness checks.

1. Introduction

China experienced rapid growth in chemical fertilizer use, particular after 2004 when the Chinese government shifted from taxing agriculture to subsidizing agricultural programs. The unit area chemical fertilizer usage is nowadays 4 times more than the world average [1]. Agricultural nonpoint-source pollution is a growing concern in China because these pollutions had become the main cause of water pollution [2]. Agricultural nonpoint-source pollution has also been found to be an important factor affecting health [3-6]. Specifically, the elderly might have a higher health risk comparing with the young people when facing environmental exposure. A higher susceptibility and higher mortality rates could be observed among the adults over the age of 75 years compared with the younger ages [7-9]. The differences of health risk might due to physiological changes associated with aging [10]; the age-related behavior trends varied between younger and older adults which might affect older adults' exposures [11]; other f[illegible]rs such as socio-economic and nutritional status [12]. China had the most serious aging problems because 70% of elderly lives in rural areas

[13]. The elderly residents might most be affected by agricultural nonpoint pollution. Another reason we focus on the rural elderly is that the health status, medical facility and diseases awareness of rural elderly are not as good as those in cities [14-15]. This paper complements the existing literature by proving new evidence on the effects of agricultural non-agricultural pollution. Air pollution as a leading problem for public health was broadly studied in agricultural nonpoint pollutions [16-17]. The work most closely related to this paper was Brained and Menon (2014) [4], which assessed the impact of fertilizer runoff on infant and child in India. We complement their study by emphasizing another vulnerable group, i.e., older adults, who are more likely to stay in countryside in the process of urbanization and most directly affected by the loss of chemical fertilizer. Another work by Lai (2017) [5] assessed the health effect of pesticide by using the pesticide cost. In order to eliminate the influence of price, we use the amount of chemical input and loss, which are more accurate. Though both chemical fertilizers and pesticides can cause water pollution, they work in different ways, fertilizer are directly spread in the soil which is more likely to affect with surface and groundwater runoff. This paper emphasizes on non-occupational fertilizer exposure because the farmers who are directly exposure to chemical fertilizer are only a small proportion of the population and had been studied [18-20]. Many more population may be at risk because of agricultural externality.

The inefficient use of chemical fertilizer has caused serious nonpoint-source pollution. Fertilizers leaded to "groundwater contamination, eutrophication of fresh water and estuarine ecosystems, tropospheric pollution related to emissions of nitrogen oxides and ammonia gas, and accumulation of nitrous oxide" [21]. A 10% increase in the use of nitrogen fertilizer and phosphorus fertilizer leaded to a 1.525% and 1.374% increase of the concentration of nitrogen and phosphorus in water, respectively [22]. In China, agricultural nonpoint-source pollution has exceeded industrial pollution in water. The chemical oxygen demand (COD) emission of agricultural pollution sources was 1.8 times of the industrial sources [23]. After 2004, in particular, China abolished agricultural tax and issued a series of agricultural support policies to promote agricultural production, which encouraged the excessive application of agricultural fertilizer. From 2004 to 2015, the amount of chemical fertilizer applications was increased by 30% (i.e., 1.386 million tons) [24], while the increase in agricultural produce output was far less. As a result of the formation of nitrite compounds and the

change in soil environmental conditions, chemical fertilizer loss may potentially lead to chronic diseases, nervous system diseases, and organ diseases [25-27], and eventually affect human health as well as medical cost (Fig 1). Amines and amides in chemical fertilizers and sewage could cause digestive tract diseases, esophageal cancer, liver cancer, stomach cancer, esophagus cancer, and other cancers [28-30]. The fertilizer, which were soluble in water and had stable properties, were transported by rivers over long distances and large ranges, and enter the human body via drinking water and other ways to synthesize nitrosamines, especially nitrosamines. Drinking water or eating vegetables with a high nitrate content may lead to thyroid cancer, hypertension, neural tube defects (NTDs) [31-32], and various chronic diseases, such as skin diseases, lung cancer, bladder cancer, and cardiovascular disease [33]. Based on the background of China's agricultural support policy, this paper verifies the effect of chemical fertilizer loss from the soil on the health of the rural elderly, and comprehensively analyzes the health damage from the perspectives of daily living ability, disease risk, and disease age onset. The economic cost of this health damage is further calculated. More specifically, 3 hypotheses are proposed as follow: (1) Fertilizer loss can cause physical damage, increase the health risk and alter the daily living ability of the elderly. (2) Because of the physical function, fertilizer loss may lead the individuals to be ill younger. (3) Due to the health damages, the medical cost will be higher in high-loss regions comparing the regions with lower fertilizer loss

Fig1. Effects of chemical fertilizer loss on health.

The rest of the paper is organized as follows. Section 2 provides data description and summary statistics. Section 3 presents empirical models as well as results and Section 4 concludes.

2. Data and summary statistics

2.1 Data collection and variables

The analysis is accomplished using difference-in-differences method with the national dataset Chinese Longitudinal Healthy Longevity Survey (CLHLS) and publicly published yearbooks.

The individual health data come from the Chinese Longitudinal Healthy Longevity Survey (CLHLS). CLHLS is chosen for this research due to its richness in health and demographic information, its nationwide

sampling areas, and its time span which covers the year of the agricultural policy change. The CLHLS was conducted in a randomly selected half of th[illegible]ounties and cities in 22 provinces, covering 85% of the total population in China. Its eight waves (1998, 2000, 2002, 2005, 2008, 2011,2014 and 2018) surveyed the cohort of 65 years and older. We only use the data from 2000 to 2014 because the survey question asking whether drinking water was boiled or not started in 2000; only those who drank boiled water are included in our analysis to eliminate possible health linkages to waterborne bacteria. China began to implement the "fertilizer reduction" policy in 2015. In order to eliminate this policy effect, we did not use the data after 2015. The survey combines an in-home interview and a basic physical examination. Extensive information was collected on demographic characteristics, family and household characteristics, lifestyles, diet, psychological characteristics, health, disability, socioeconomic conditions, etc. Fertilizer input data come from the China Rural Statistical Yearbook (1999-2015) and other regional variables come from the China Statistical Yearbook (1999-2015). Chemical fertilizer loss index come from Data collection of the first national survey of pollution sources (2011).

2.1.1 Health outcome variables

Multiple indicators for health status of the elderly are examined linked to fertilizer loss in the extant medical and toxicology literatures, including the index of Activities of Daily Living (ADL), the numbers of diseases related to fertilizer loss, and age of onset.

The ADL index is widely used to measure the dependence of the elderly and physically inactive people. It is also known as the "disability" index [34]. The ADL index is constructed by asking participants whether they need help with six basic activities (namely bathing, dressing, eating, going to the toilet, indoor movement, and controlling defecation). The choices for each item are "can do without help" (corresponding score is 1), "need help" (corresponding score is 2), and "need full help" (corresponding score is 3). The final score of the ADL index is the sum of all six items, ranging from 6 to 18. Higher scores mean that the health status of the elderly is worse.

The number of diseases measures how many types of diseases related to fertilizer loss are suffered by a sample individual. Although the CLHLS database investigated 16 diseases in the elderly, including hypertension, heart disease, cancer, stroke, cardiovascular and cerebrovascular diseases, bronchitis, emphysema, asthma, and pneumonia, only three diseases—hypertension, cancer, and gastrointestinal ulcer—were related to fertilizer loss in the existing medical literature.

The age of onset refers to the age at which a sample individual first suffered from any of the three diseases (i.e., hypertension, gastrointestinal ulcer, or cancer).

2.1.2 Individual and household characteristic variables

Individual and household characteristic variables include information on socio-economic characteristics, health behavior and dietary pattern. Socio-economic characteristics include age, gender, people living together, income station, basic health condition and hospitalization. Health behaviors measure whether the respondents smoke, drink or do any exercise (with options of "Yes" or "No"). Dietary patterns measure the frequency with which respondents eat meat, fish, egg, and salt-preserved vegetables. Options for each item are "rarely or never", "not every month, but occasionally", "not every week, but at least once per month", "not every day, but at least once per week", "almost every day", with scores from 1 to 5, respectively. The questionnaire also asks the sources of respondents' drinking water. Options include drinking water sources from a well, a river of lake, a spring, a pond or pool, and tap water. A binary variable (Water) is constructed, which equals one if respondents drink surface water, i.e. water from a rive or lake or a pond of pool, and equals zero otherwise.

2.1.3 Provincial level variables

Provincial level variables include fertilizer loss, fertilizer use, organic fertilizer use, pesticide use, industrial pollution index, the numbers of hospital and provincial Gross Domestic Product (GDP). The calculation method for fertilizer loss intensity is based on literature [35]. On the basis of the unit investigation method and the inventory analysis method, the fertilizer loss coefficient is used to calculate the total nitrogen and phosphorus emissions; then the total emission and emission intensity of fertilizer nonpoint-source pollution are calculated. The calculation formula is as follows:

$$E = \sum E_{ij} = \sum C_{ij} \times \eta_{ij} \quad (1)$$

$$I = E/A \quad (2)$$

where E measures the total emission of fertilizer loss; E_{ij} measures the amount of the pollutant j produced and the loss into water by province i; C_i measures the index statistics of pollutant i (compound fertilizer is converted into total nitrogen 40% and phosphorus pentoxide 32%); η_{ij} measures the loss coefficient; A represents the crop areas; and I represents pollution emission per unit area, i.e., pollution intensity (unit: kg/ha).

The industrial pollution index ($Indpollution_i$) controls industrial activities, which is defined in Equation (3) according to existing literature [36-37]. GDP measures general economy and wealth conditions. The number of hospitals controls the level of medical service. Because the health data are observed once three years, all provincial variables are assigned the average of the last three years.

$$Indpollution_i = \frac{pv_{i1} + pv_{i2} + pv_{i3}}{3}, \quad pv_{ij} = p_{ij} \Big/ \sum_{i=1}^{n} \frac{p_{ij}}{n} \quad (3)$$

where p_{ij} measures the pollutant j of province i, n is the total number of provinces, pv_{ij} measures the emission index of pollutant j in the province i relative to the national average level. The larger value means the higher pollution level. $pv_{i1}, pv_{i2}, pv_{i3}$ represents industrial wastewater, industrial sulfur dioxide and industrial dust, respectively.

2.2 Statistical analyses

2.2.1 Sample summary

The statistical descriptions of all variables used in the regression are summarized in Table 1.

Table 1. Variable description and summary statistics.

Variables	Description	Obs.	Mean	Std.Dev.	Min	Max
**Household Variables**						
Age	Age in years	39093	85.83	10.83	65	109
Male	Male=1;Female=0	39093	0.44	0.50	0	1
Co-residence	Nursing home=1; Alone or Spouse=2; Child=3; Others=4	39093	2.62	0.57	1	4
Income_cost	If income support daily cost (Yes=1; No=0)	39093	0.78	0.41	0	1

Illness	Number of 15 chronic diseases	39093	0.85	1.06	0	1
Hospitalization	The times of hospitalization in two years	39093	0.26	0.79	0	30
**Health Behaviors**						
Smoke	Currently smoke (Yes=1; No=0)	39093	0.33	0.47	0	1
Drink	Currently drink (Yes=1; No=0)	39093	0.31	0.46	0	1
Exercise	Currently exercise (Yes=1; No=0)	39093	0.27	0.44	0	1
Dietary Pattern						
Meat	The frequency of meat consumption	39093	3.95	1.03	1	5
Fish	The frequency of fish consumption	39093	3.36	1.15	1	5
Egg	The frequency of egg consumption	39093	3.92	1.07	1	5
Salt_vege	The frequency of salt-preserved vegetable consumption	39093	3.27	1.38	1	5
Boiled water	If drink boiled water (boild water=0; not boild water=1)	39093	0.057	0.23	0	1
Water	Drirking water source (Surface water=1; Tap water=0)	39093	0.14	0.35	0	1
**Provincial Variables**						
Floss	Fertilizer loss intensity (kg/ha/year)	39093	7.34	3.76	0.60	14.82
Finput	Fertilizer input intensity (kg/ha/year)	39093	354.80	92.47	128.41	704.32
Pinput	Pesticide inptut intensity (kg/ha/year)	39093	12.62	6.56	2.28	22.26
OFinput	Organic fertilizer input intensity (kg/ha/year)	39093	286.53	361.18	11.56	1997.00
Hospnum	The number of hospital per province (million)	39093	0.02	0.02	0.01	0.08
LnGDP	Logarithm of Provincial Gross Domestic Product	39093	9.10	0.91	7.32	11.04
Indpolltion	Index of industrial pollution (lower=better)	39093	1.46	0.55	0.45	2.95
**Health indicatos**						
ADL	Activities of Darly Living score (lower=better)	39093	6.66	1.80	6	18
No. of diseases	The number of illness caused by fertilizer loss (lower=better)	39093	0.25	0.48	0	3
Age	Age of suffering from illness caused by fertilzer loss (lower=worse)	9503	83.70	10.06	65	109
Mescost	Medical cost(RMB)	39093	2768.29	11676.91	0	199996

2.2.2 Fertilizer loss and health outcome after agricultural support policies

China shifted from taxing agriculture to subsidizing agricultural programs since 2004 to maintain food security and self-reliance. In 2004, authorities introduced three subsides targeted at grain producers: a direct payment for grain producers, a subsidy for improved seed varieties and a partial rebate for farm machinery purchases. Several other support programs were introduced such as a general-input subsidy, price floors for wheat and rice, reform of the grain marketing system and transfer payment to grain counties since 2004, which has been accompanied with greater fertilizer use and fertilizer loss. This set of supporting programs had bolstered farmer' production incentives and crop production increased continuously since 2004 [38-39], on the

other hand, the programs might lead to the distortion of agricultural factors and cause negative effects on soil and water environment [40]. Table 2 reports the differences of health outcomes and fertilizer loss between regions with high versus low intensity of fertilizer loss. We report the differences of ADL index, the numbers of induces diseases, intensity of fertilizer input, amount of fertilizer loss and intensity of fertilizer loss before the implementation of agricultural support policies in column (3). The differences after the agricultural support policies are in column (6). The widening gap in health outcomes and fertilizer loss and input can be observed in column (7) between regions with high and low fertilizer loss after the agricultural support policies. Preliminary data analysis shows that the loss of chemical fertilizer in high-loss areas was more serious after the agricultural subsidy policies.

Table 2. Comparison of high-loss-areas and low-loss areas by agricultural support policies.

Variables	Before agricultural Support Policies			After Agricultural Support Policies			2nd difference (7)=(6)-(3)
	Low loss (1)	High loss (2)	1st difference (3)=(2)-(1)	Low loss (4)	High loss (5)	1st difference (6)=(5)-(4)	
ADL index	6.823	7.022	0.199	6.822	7.108	0.287	0.088
No. of diseases	0.160	0.211	0.051	0.255	0.340	0.085	0.035
Input intensity (kg/ha)	239.562	358.413	118.851	317.519	457.163	139.644	20.793
Total loss (10000 tons)	1.689	6.717	5.028	2.023	7.350	5.327	0.299
Loss intensity (kg/ha)	2.905	8.599	5.694	3.491	9.226	5.735	0.041

Notes:
The fertilizer loss is continuous variable, and we dichotomized fertilizer loss by its pre-policy mean to compare the health effect in different regions. The average value of fertilizer loss is the national average of fertilizer loss; it is equal to 5.36kg/ha.
We showed the fertilizer input and fertilizer loss in S1 Fig.

2.2.3 Age of onset and fertilizer loss

The CLHLS contains three diseases (namely hypertension, gastrointestinal ulcer and cancer) induced by fertilizer loss. The age, when one sample first suffers from any of the three diseases, is defined as age of onset. The age of onset comparisons across age groups are represented by Fig 2. A significant difference of age of onset can be observed between the regions with high versus low intensity of fertilizer loss in younger-old groups (< 80 years) in Panel A of Fig 2. The age of onset is not significantly different in the oldest-old samples.

Fig 2. Chemical fertilizer loss and age of onset.

3. Empirical models and results

This section investigates the health effect of fertilizer loss on local elderly population. Preliminary data analysis reports the health outcomes are different in regions with high verse low intensity of fertilizer loss after agricultural subside policies in 2004. Further, we follow a difference-in-differences (DID) framework to compare health outcomes between people in regions with different intensities of fertilizer loss before and after 2004, when China shifted from taxing agricultural outputs to subsidizing agriculture. The medical cost is estimated as well. To facilitate comparisons across age groups, we explore heterogeneous effects by splitting the sample in various age groups and reporting estimates of α_1 and β_1 for these subsamples. Then several robustness checks are conducted to help assess the validity of our results. The difference-in-differences model is specified as:

$$Health_{it} = \alpha_0 + \alpha_1 Fertilizerloss_{it} \times Time_{it} + \alpha_2 Fertilizerloss_{it} + \alpha_3 Time_{it} + \alpha_4 X_{it} + \gamma_t + \mu_i + \varepsilon_{it} \quad (4)$$

$$Health_{it} = \beta_0 + \beta_1 Area_{it} \times Time_{it} + \beta_2 Area_{it} + \beta_3 Time_{it} + \beta_4 X_{it} + \gamma_t + \mu_i + \varepsilon_{it} \quad (5)$$

$$Age_{it} = \delta_0 + \delta_1 Fertilizerloss_{it} \times Time_{it} + \delta_2 Fertilizerloss_{it} + \delta_3 Time_{it} + \delta_4 X_{it} + \gamma_t + \mu_i + \varepsilon_{it} \quad (6)$$

$$Age_{it} = \theta_0 + \theta_1 Area_{it} \times Time_{it} + \theta_2 Area_{it} + \theta_3 Time_{it} + \theta_4 X_{it} + \gamma_t + \mu_i + \varepsilon_{it} \quad (7)$$

where $Health_{it}$ denotes the health outcome; Age_{it} denotes the age of onset; α_1, β_1, δ_1 and θ_1 identify the effects of fertilizer loss; $Fertilizerloss_{it}$ denotes the fertilizer loss of the region where individual i resides; $Area_{it}$ is a dummy variable equaling 1 if the individual is in high loss areas, and 0 otherwise; $Time_{it}$ equals 1 starting in 2004 and 0 otherwise; X_{it} is a vector of control variables including individual, household, and provincial characteristics such as age, gender, health behavior, dietary habits, industrial pollution and hospitals, etc.; μ_i is a fixed effect unique to individuals i; γ_t is a time effect common to all individuals in year t; and ε_{it} is an error term.

The relationship between medical cost and health can be identified in the following Equation:

$$Y_{it} = \alpha_0 + \alpha_1 ADL_{it} + \alpha_2 X_{it} + \gamma_t + \mu_i + \varepsilon_{it} \quad (8)$$

where Y_{it} represents medical costs, and other variables are defined as before.

3.1 Basic regression results of health

3.1.1 Effect of fertilizer loss on health

Table 3 reports the α_1 and β_1 coefficients in Equation (4) and Equation (5) for outcomes related to ADL index and No. of diseases. Estimated coefficients in column (1) of Table3 indicate that one kg/ha increase of chemical fertilizer loss is associated with increased ADL daily life index of 0.0147(0.2 percent). From column (4) one kg/ha increase of fertilizer loss is associated with 0.0057 raise in the number of diseases (0.5 percent). These results indicate that the fertilizer loss increase the elderly's health risks and support diseases significantly. For different regions, estimates for β_1 in columns (2) and column (5) of Table 3 indicate a reduction in the ADL index and the number of diseases for high-loss area verse low-loss areas. Compared with low-loss areas, the ADL index of the rural elderly is 0.1222 higher in areas with high chemical fertilizer loss, while the number of diseases is 0.0487 higher. We also use the two-way fixed effects model to estimate the health effect of fertilizer loss. Column (3) and Column (6) of Table 3 report the coefficients for outcomes related to ADL index and No. of diseases. Estimated coefficients indicate that one kg/ha of fertilizer loss increase is associated with increased ADL daily life index of 0.1088. Column (6) shows that one kg/ha of fertilizer loss increase is associated with 0.0211 raise in the number of diseases. The health effect of agricultural fertilizer input not only accrue in farmers who are likely to receive a fertilizer loss via direct exposure. Many more proportions of the population are at risk of fertilizer loss because of exposure to contaminated water and soil. Agricultural support policies play an important role in the fertilizer loss, and the agricultural support policies may cause heterogeneous fertilizer loss between regions because of the different subsides levels. These may cause the biased result of TWFE. DID model can avoid this problem, and we control the time effect and individual effect in our regressions.

Table 3. Basic estimates ADL index and diseases.

	ADL			No. of diseases		
	DID		TWFE	DID		TWFE
	(1)	(2)	(3)	(4)	(5)	(6)
Fertilizerloss*Time	0.0147**	--	--	0.0057***	--	--
	(0.0060)	--	--	(0.0014)	--	--
Higharea*Time	--	0.1222***	--	--	0.0487***	--
	--	(0.0461)	--	--	(0.0114)	--
Fertilizer loss	--	--	0.1088**	--	--	0.0211*

	--	--	(0.0432)	--	--	(0.0126)
Control	YES	YES	YES	YES	YES	YES
Year FE	YES	YES	YES	YES	YES	YES
ID FE	YES	YES	YES	YES	YES	YES
Observations	32165	32165	38908	32329	32329	38680
R-squared	0.5570	0.5582	0.3183	0.6906	0.6907	0.3243

Notes:
*, **, *** are significant at the level of 10%, 5% and 1% respectively.
The estimated results without control variables were in S1 Table.
Considering the nonlinear health factors, we further estimated the health effects of fertilizier loss using propensity score matching (PSM) difference-in-differences (DID) model. The results were in S2 Table.

3.1.2 Heterogeneous health effects

In S3 and S4 Tables, we explore the heterogeneous effects by splitting the samples in different age groups and reporting estimates of α_1 for these subsamples. First, the oldest olds (≥80years) were discussed in previous studies [41], because the oldest olds had greater risk of falling and hospitalization [42-44]. We divide the samples at age into the younger age samples (<80years) and the oldest olds samples (≥80 years). In Columns (1) and columns (2) of S3 Table, a positive and significant estimate of α_1 can be observed in the oldest olds. The estimates of α_1 are positive and significantly for both age groups (columns (3) and columns (4) of S3 Table). The fertilizer loss will increase the risk of diseases in both the younger olds and the oldest olds. The grouped estimates for effect of fertilizer loss on ADL and diseases of the oldest old are significantly distinguishable from the younger olds at the 1% level. We also divide the samples into younger olds (65years-75years), middle olds (75-85years) and the oldest olds (≥85years) [45-47]. The differences between younger and middle olds are not significant in both ADL and No. of diseases. The coefficient for oldest olds is larger than that for younger and middle olds, and the differences are significant.

3.1.3 Effect of fertilizer loss on age of onset

This study further explores whether fertilizer loss will lead to the elderly be sick in younger age. Though from Fig 2, the age of onset of elderly in areas with high-loss of chemical fertilizer are younger, in order to understand the relationship between fertilizer loss and the age of onset, we use the difference-in-differences model for empirical analysis. Estimated coefficient in column (1) of Table 4 indicates that one kg/ha increase

of fertilizer loss advances the age of onset by 0.2670 year. Compared with low-loss areas, the age of onset declined by 0.3753 in high-loss areas.

Table 4. Estimation of fertilizer loss on age of onset.

	Age of Onset	
	(1)	(2)
Fertilizerloss*Time	-0.2670*<br>(0. 0161)	
Higharea*Time		- 0.3753**<br>(0. 1258)
Control Variables	YES	YES
Year FE	YES	YES
ID FE	YES	YES
Observations	9503	9503
R-squared	0.9822	0.9822

Notes:
*, **, *** are significant at the level of 10%, 5% and 1% respectively.

3.2 Results of health conditions on medical cost

In this section, we continu[illegible]plore these results by translating estimated effects into expected monetary losses. We do this by exploiting the relation between ADL and medical cost in Equation (8). Estimated coefficient in column (1) of Table 5 indicates that each additional unit increase in ADL index increases the medical cost by CNY 244.6 (USD 34.94). Columns (2) and (3) report the λ_1 coefficient in Equation (8) in high-loss and low-loss areas, respectively. The estimates indicate that there is a significant difference in medical cost between high loss areas and low loss areas. Every one-unit ADL increase is associated with a higher medical cost of CNY 194.64 (USD 27.81) in high loss areas. The health cost per unit loss of chemical fertilizer (1kg/ha) is about CNY 3.64 (USD 0.51). Though agro-chemical use is associated with significantly greater agricultural output value a range studies showed that agro-chemical also damaged human health. Indirectly, medical cost increases and labor supply loss could be observed due to illness [48]. Similar to our study, Lai suggested that a 10%(CNY 0.2/USD 0.03) increase in rice pesticide use will add 168.8 and 55.89 million dollars to medical costs and offspring’s human capital losses, respectively [6]. According to the sixth national census, there were about 87.91 million rural elderly people (over 65 years of age) in China. We estimate the economic loss of fertilizer loss, an increase of one kg/ha of fertilizer loss and fertilizer input will add CNY 316 (USD 45) million and CNY 21.5 (USD 3.1) million to national medical cost, respectively.

Table 5. Effect of health on medical cost.

	Medical Cost		
	Overall samples	High-loss area	Low-loss area
ADL index	244.60***	331.94***	137.30**
	(55.81)	(93.54)	(63.58)
Control Variables	YES	YES	YES
Year FE	YES	YES	YES
ID FE	YES	YES	YES
Observations	23087	19875	15167
R-squared	0.0709	0.0226	0.0121
p			0.0853*

Notes:
*, **, *** are significant at the level of 10%, 5% and 1% respectively.

3.3 Robustness check

3.3.1 Parallel trend test

In order to test whether the time staggered entry events of agricultural policy are effective exogenous shocks, we conduct a parallel test bas[illegible]n Equation (9):

$$H_{it} = \alpha + \sum_{k\geq -2, k\neq -1}^{k=4} \delta_k D_{it,k} \times Fertilizerloss_{it,k} + \lambda X_{it} + \gamma_t + \mu_i + \varepsilon_{it} \quad (9)$$

where $D_{it,k}$ is a binary variable that takes value of 1 when agricultural support policies are implemented. We use $k=-1$ to denote the base year. S2 Fig, Panel A and Panel B plots estimates of ADL index and No. of diseased, respectively. The pre-period coefficients are statistically indistinguishable from zero and there is a sharp, statistically significant and sustained change after the policy implementation. The post-coefficients are significant. We also adopt a binary variable that takes value of 1 when the individuals in high loss areas. The estimates coefficients were shown in S2 Fig Panel C and Panel D.

3.3.2 Placebo test

In order to further test whether the results are driven by unobservable factors at the individual or provincial level, referring to the methods of existing literature [49-50], this study conducted a placebo test by randomly assigning high and low chemical fertilizer loss areas. Specifically, 9 out of 19 provinces are randomly selected as the experimental group, assuming that these 9 provinces are areas with high loss of chemical fertilizer, and other regions are the control group (areas with low loss of chemical fertilizer). In this study, random sampling is carried out 1000 times, and the benchmark regression is performed according to Equation (5). S3 Fig reports the average value of the ADL index and the number of induced diseases after

1000 random distributions. The mean value of the 1000 random sample regressions is almost 0. The placebo test shows that our basic estimation results are unlikely to be driven by unobservable factors at the individual or provincial level.

3.3.3 Effect of fertilizer input on health

In order to verify the robustness of the research conclusions, the application intensity of chemical fertilizer and the areas with high and low chemical fertilizer application intensities are used to test the robustness. According to the criteria of ecological counties in China (the application intensity of chemical fertilizer should not exceed 250 kg/ha), the areas with a chemical fertilizer application intensity exceeding 250 kg/ha are defined as high input areas, and the areas with a chemical fertilizer application intensity not exceeding 250 kg/ha are defined as low input areas. The basic regression of Equations (4) and (5) are carried out using the chemical fertilizer application intensity. The model results are shown in S5 Table. The model results are essentially consistent with the benchmark regression results, indicating that the research results are robust. For every 1 kg/ha increase in fertilizer input intensity, the ADL index will increase by 0.001 and the number of induced diseases will increase by 0.0001.

3.3.4 Effect in different areas

In order to verify the robustness of the analysis of this study, different samples are used This paper focuses on health effect of agricultural fertilizer loss of the rural elderly. Therefore, samples from major agricultural-production areas, major rice-producing areas and water resource-rich areas are used to carry out the basic regression of Equation (4) and Equation (5). The results (S6 and S7 Tables) show that fertilizer loss leads to a significant increase both on ADL index and the number of diseases in major agricultural-producing areas, major rice-producing areas and water resource-rich areas, which are basically the same comparing with results in Table3.

4. Conclusions and discussion

China's agricultural fertilizer input is facing a problem of "high input, high loss, and high pollution", which has been aggravated by agricultural support policies since 2004. The problem of the "Three Highs" of chemical fertilizer use has caused pollution to the environment and health damages to rural residents. In this paper, the chemical fertilizer loss data and CLHLS micro-health data are used to verify the health effect of fertilizer loss on the rural elderly in China. Nearly 60% elders live in rural areas in China and the number of the elders might increase to 450 million (nearly one third of the total population) by 2050[51]. The health effect of fertilizer maybe a greater challenge to pension and health care in rural China.

To improve the health status of elderly in rural areas, the government can consider from agricultural policy and public health policy. (1) fertilizer loss can be reduced by innovating agricultural production technology research and promoting technology adoptions. e.g. soil testing, formula fertilization technology, slow-release fertilizer technology, deep tillage machinery promotion, and straw returning technology. (2) the government should improve soil quality to increase nutrient preserving capability of soil, such as soil improvement technology, etc. (3) the government need to focus on the rural drinking water, and to improve the rural elderly's awareness of drinking safety and the risk of health damages due to chemical fertilizer loss.

The health effects of agricultural nonpoint-source pollution are estimated by taking the fertilizer as an example. Due to the lim[illegible]ions of the article space, this article does not discuss the health effect of other agricultural pollution, such as pesticides use, straw combustion and the interaction of various agricultural pollutions. The nonpoint-source pollution in animal husbandry and fishery is also worth discussing.

References

1. NBSC. 2016. China Statistical Yearbook. Beijing, National Bureau of Statistics of China.
2. Zhou K, Fan J, Liu HC. 2017. Spatiotemporal patterns and driving forces of water pollutant discharge in the Bohai Rim region. Progress in Geography. 2020; 36(02): 171-181.
3. Sun XY, Wang J, Jin YT. Advances in research on pesticide pollution to the aquatic environment and health impact in China. Journal of Environment and Health. 2009;26(07): 649-652.

4. Brainerd E, Menon N. Seasonal effects of water quality: The hidden costs of the green revolution to infant and child health in India. Journal of Development Economics. 2014; 107: 49-64. doi: 10.1016/j.jdeveco.2013.11.004.
5. Lai WY. Pesticide use and health outcomes: evidence from agricultural water pollution in China. Journal of Environmental Economics and Management. 2017; 86: 93-120. doi:10.2139/ssrn.2751458.
6. He GJ, Liu T, Zhou MG. Straw burning, PM 2.5, and death: Evidence from China. Journal of Development Economics. 2020;145: 102468. doi: 10.1016/j.jdeveco.2020.102468.
7. Wong CM, Lai HK, Tsang H, Thach TQ, Thomas GN, et al. Satellite-based estimates of long-term exposure to fine particles and association with mortality in elderly Hong Kong residents. Environmental Health Perspectives. 2015;123(11):1167-1172. doi: 10.1289/ehp.1408264. Epub 2015 Apr 24. PMID: 25910279.
8. Sacks JD, Stanek LW, Luben TJ, Johns DO, Buckley BJ, et al. Particulate matter-induced health effects: who is susceptible? Environmental Health Perspectives. 2011;119(4):446-54. doi:10.1289/ehp.1002255. PMID: 20961824.
9. Cohen, G., Gerber, Y. Air pollution and successful aging: Recent evidence and new perspectives. Current Environmental Health Reports. 2017; 4:1-11. https://doi.org/10.1007/s40572-017-0127-2. PMID: 28101729.
10. Assembly of First Nations (AFN). Environmental health older adults and seniors (elders). Ottawa, Canada. 2009.
11. Tuttle L, Meng Q, Moya J, Johns DO. Consideration of age-related changes in behavior trends in older adults in assessing risks of environmental exposures. Journal of Aging and Health. 2013; 25(2):243-273. doi:10.1177/0898264312468032. PMID: 23223208
12. Health Canada. Health of older adults and the environment discussion paper. Ottawa, Canada. 2008.
13. Tong YF. Changes and challenges of labor supply in China in the context of population ageing. Population Research. 2014; 38(02): 52-60.
14. Li JX, Li CH. Health difference of the elderly between the rural and urban districts. Population Journal. 2014; 36(05):37-47.
15. Weeks WB, Kazis LE, Shen TJ, Cong ZX, Ren XH, et al. Differences in health-related quality of life in rural and urban veterans. American Journal of Public Health. 2004; 10:1762-1767. https://doi.org/10.2105/AJPH.94.10.1762.
16. Khanal SJ, Pokhrel RP, Pokharel B, Becker S, Giri B, et al. An episode of transboundary air pollution in the central Himalayas during agricultural residue burning season in North India. Atmospheric Pollution Research. 2022; 13(1): 101270. https://doi.org/10.1016/j.apr.2021.101270.
17. Huang L, Zhu YH, Wang Q, Zhu AS, Liu ZY, et al. Assessment of the effects of straw burning bans in China: Emissions, air quality, and health impacts. Science of The Total Environment. 2021; 789: 147935. https://doi.org/10.1016/j.scitotenv.2021.147935.
18. Nganchamung T, Robson M. Chemical fertilizer use and acute health effects among Chili farmers in Ubon Ratchathani Province, Thailand. Journal of Health Research.2017; 31(6): 427-435. doi:10.14456/jhr.2017.53
19. Gorman NM, Stjernberg E, Koehoorn M, Demers PA, Winters M, et al. Fertilizer use and self-reported respiratory and dermal symptoms among tree planters. Journal of Occupational and Environmental Hygiene. 2013; 10(1): 36-45. doi: 10.1080/ 15459624.2012.740994.
20. Qiao F, Huang J, Zhang L, Rozelle S. Pesticide use and farmers' health in China's rice production. China Agricultural Economic Review. 2012; 4: 468-484. https://doi.org/10.1108/17561371211284821.
21. Zhang WD, Sohngen B. Do U.S. anglers care about harmful algal blooms? A discrete choice experiment of Lake Erie recreational anglers. American Journal of Agricultural Economics, Agricultural and Applied Economics Association.2018; 100(3): 868-888. https://doi.org/10.1111/ajae.12130
22. Paudel J, Crago CL. Environmental externalities from agriculture: Evidence from water quality in the United States. American Journal of Agricultural Economics. 2021; 103: 185-210. https://doi.org/10.1111/ajae.12130

23. China pollution source census. Technical report on China pollution source census. Beijing: China Environmental Press, 2011.
24. National Bureau of Statistics. China statistical yearbook (2019). Beijing: China Statistics Pres, 2020.
25. Zahra, Bahadoran, Asghar. Nitrate-nitrite-nitrosamines exposure and the risk of type 1 diabetes: A review of current data. World Journal of Diabetes. 2016; 7:433. doi: 10.4239/wjd.v7.i18.433. PMID: 27795817
26. Fan AM, Steinberg VE. Health implications of nitrate and nitrite in drinking water: An update on methemoglobinemia occurrence and reproductive and developmental toxicity. Regulatory Toxicology and Pharmacology.1996; 23:35-43. doi:10.1006/rtph.1996.0006.
27. Kristensen P, Andersen A, Irgens LM. Testicular cancer and parental use of fertilizers in agriculture. Cancer epidemiology, biomarkers & prevention. 1996; 5:3-9. PMID: 8770459
28. Ward MH. Too much of a good thing? Nitrate from nitrogen fertilizers and cancer. Reviews on Environmental Health. 2009; 24, 357-363. doi:10.1515/REVEH.2009.24.4.357.
29. Forman D. Are nitrates a significant risk factor in human cancer? Cancer Surveys.1989; 8(2): 443-458. PMID: 2696589
30. Bivolarska A, Gatseva P. Thyroid status in pregnant women and association with nitrates as an environmental factor stimulating the manifestation of iodine deficiency. Trace Elements and Electrolyte. 2015; 32:60-64. doi: 10.5414/tex01365.
31. Manasaram DM, Backe LC, Moll DM. A review of nitrates in drinking water: Maternal exposure and adverse reproductive and developmental outcomes. Environmental Health Perspectives. 2006; 114: 320-327. doi:10.2307/3436672.
32. Uddin M.S, Kurosawa K. Effect of chemical nitrogen fertilizer application on the release of arsenic from sediment to groundwater in Bangladesh. Procedia Environmental Sciences. 2011; 4: 294-302. doi: 10.1016/j.proenv.2011.03.034.
33. Smith AH, Hopenhayn-Rich C, Bates MN. Cancer risks from arsenic in drinking water. Environmental Health Perspectives. 1992; 97:259. doi: 10.1289/ehp.9297259.
34. Johnson RJ, Wolinsky FD. The structure of health status among older adults: disease, disability, functional limitation, and perceived health. Journal of Health Social Behavior. 1993; 34: 105-121. doi:10.2307/2137238
35. Lei J, Su SP, Yu WM. Temporal and spatial pattern evolution and grouping prediction of non-point source pollution of chemical fertilizers in China. Chinese Journal of Eco-Agriculture. 2020; 28(07): 1079-1092. doi: 10.13930/j.cnki.cjea.190923.
36. Shen KR, Jin G, Fang X. Dose environmental regulation cause pollution to transfer nearby? Economic Research Journal. 2017; 52(05): 44-59. http://www.cesgw.cn/cn/mlInfo.aspx?m=20170217100514860651&n=20170609120846613735&tip=4.
37. Zhu FP, Zhang ZY, Jiang GL. Empirical study of the relationship between FDI and environmental regulation: An intergovernmental competition perspective. Economic Research Journal. 2011; 46(06): 133-145. http://www.cesgw.cn/cn/mlInfo.aspx?m=20110125121729123417&n=20110617154203400510&tip=10
38. Chen HP, Wu LP, Wang YB. Impacts of subsidy policy on grain production: An empirical analysis based on sub-provincial data of grain production from 2004—2007. Journal of Agro technical Economics. 2010; 4: 100-106.
39. Chen F, Fan QQ, Gao TM. Agricultural policies, food production and food production-adjustment ability. Economic Research Journal. 2010; 45(11): 101-114+140.
40. Ge JH, Zhou SD. An analysis of the economic determinants to agricultural non-point source pollution: Based on data of Jiangsu province during 1978-2009. Chinese Rural Economy, 2011; (5): 72-81.
41. Callaway B, Goodman-Bacon A, and Pedro H.C. Sant'Anna. Difference-in-differences with a continuous treatment. Working Paper, 2021.
42. Brettschneider C, Hajek A, Rohr S, Fuchs A, Weeg D, et al. Determinants of health-care costs in the oldest-old in Germany. The Journal of the Economics of Ageing. 2019; 14: 100200. https://doi.org/10.1016/j.jeoa.2019.100200.

43. Winograd CH. Targeting Strategies: An overview of criteria and outcomes. Journal of the American Geriatrics Society. 1991; 39:25-35. https://doi.org/10.1111/j.1532-5415.1991.tb05930.x
44. Speechley M, Tinetti M. Falls and injuries in frail and vigorous community elderly persons. Journal of the American Geriatrics Society. 1991; 39:46-52. doi: 10.1111/j.1532-5415. 1991.tb05905. x. PMID: 1987256.
45. Rockwood K, Stadnyk K, MacKnight C, McDowell I. A brief clinical instrument to classify frailty in elderly people. The Lancet. 1999; 353:205-206. doi: 10.1016/S0140-6736(98)04402-X. PMID: 9923878.
46. Lau LK, Wee SL, Mallya JU, Yap, Pang, et al. Physiological and cognitive determinants of gait in middle-aged, older-aged and oldest-aged Asian adults – The Yishun study. Aging and Health Research. 2021; 1(3): 100030. https://doi.org/10.1016/j.ahr.2021.100030.
47. Monti A, Doulazmi M, Nguyen-Michel VH, Pautas E, Mariani J, et al. Clinical characteristics of sleep apnea in middle-old and oldest-old inpatients: symptoms and comorbidities. Sleep Medicine. 2021; 82: 179-185. https://doi.org/10.1016/j.sleep.2021.04.017.
48. Sheahan M, Barrett CB, Goldvale, C. The unintended consequences of agricultural input intensifycation: human health implications of agro-chemical use in Sub-Saharan Africa. Working Paper Series. 2016; 234. https://www.afdb.org/en/documents/document/working-paper-234-the-unintended-consequences-of-agricultural-input-intensification-human-health-implications-of-agro-chemical-use-in-sub-saharan-africa-87634.
49. Cai XQ, Lu Y, Wu MQ. Does environmental regulation drive away inbound foreign direct investment? evidence from a quasi-natural experiment in China. Journal of Development Economics. 2016; 123: 73-85. doi: 10.1016/j.jdeveco.2016.08.003.
50. Ferrara LE, Chong A, Duryea S. Soap operas and fertility: evidence from Brazil. Applied Economics. 2012; 4: 1-31. doi:10.2139/ssrn.1820921.
51. Dai BZ. The old age health security in rural China: where to go? International Journal for Equity Health. 2015; 14: 119. doi: 10.1186/s12939-015-0224-5.

Supporting information

S1 Fig. Fertilizer input and fertilizer loss in China.

S2 Fig. Parallel trend test.

S3 Fig. Placebo test.

Notes:
Panel 1 is the result of the ADL index placebo test; Panel 2 is the result of the placebo test of the number of patients.
The X-axis represents the estimated coefficients from 1000 random assignments.
The curve is the estimated kernel density distribution. The vertical type is a true estimate in columns (2) and (4) of Table 3.

S1 Table. Basica regression results without control variables.

	ADL		No. of diseases	
	(1)	(2)	(3)	(4)
Fertilizerloss*Time	0.0175***		0.0052***	
	(0.0057)		(0.0016)	
Higharea*Time		0.1608***		0.0441***
		(0.0432)		(0.0124)
Control	NO	NO	NO	NO
Year FE	YES	YES	YES	YES
ID FE	YES	YES	YES	YES
Observations	32,302	32,302	32,467	32,467
R-squared	0.5474	0.5475	0.5597	0.5597

Notes:
*, **, *** are significant at the level of 10%, 5% and 1% respectively.

S2 Table. Estimates of PSM-DID.

	Nearest neighbor		Radius		Kernel	
	ADL	No. of diseases	ADL	No. of diseases	ADL	No. of diseases
Fertilizerloss*Time	0.0695**	0.0143**	0.0267***	0.0065***	0.0267***	0.0065***
	(0.0273)	(0.0064)	(0.0085)	(0.0023)	(0.0085)	(0.0023)
Control	YES	YES	YES	YES	YES	YES
Year FE	YES	YES	YES	YES	YES	YES
ID FE	YES	YES	YES	YES	YES	YES
Observations	4532	4532	17801	17801	17801	17801
R-squared	0.5927	0.7152	0.5828	0.7049	0.5828	0.7049

Notes:
*, **, *** are significant at the level of 10%, 5% and 1% respectively.

S3 Table. Heterogeneous estimation of yonger olds and oldest olds.

	(1)	(2)	(3)	(4)
	ADL		No. of diseases	

	Younger olds	Oldest olds	Younger olds	Oldest olds
Fertilizerloss*Time	-0.0006	0.0356***	0.0033**	0. 0086***
	(0.0038)	(0.0074)	(0.0014)	(0.0114)
Control	YES	YES	YES	YES
Year FE	YES	YES	YES	YES
ID FE	YES	YES	YES	YES
Observations	20536	22367	20620	22431
R-squared	0.0761	0.1359	0.3418	0.2951
p	0.0008***		0.0204**	

Notes:
*, **, *** are significant at the level of 10%, 5% and 1% respectively.

S4 Table. Heterogeneous estimation of yonger olds, middle olds and oldest olds.

	(1)	(2)	(3)	(4)	(5)	(6)
		ADL			No. of diseases	
	Younger olds	Middle olds	Oldest olds	Younger olds	Middle olds	Oldest olds
Fertilizerloss*Time	0.0027	0.0000	0.0334***	0. 0018	0.0039*	0. 0074***
	(0.0040)	(0.0061)	(0.0071)	(0. 0022)	(0. 0021)	(0.0114)
Control	YES	YES	YES	YES	YES	YES
Year FE	YES	YES	YES	YES	YES	YES
ID FE	YES	YES	YES	YES	YES	YES
Observations	8332	10719	23852	8360	10769	23922
R-squared	0.0548	0.0885	0.1287	0.3416	0.3398	0.2954
p		P^{12}=0.7163			P^{45}=0.4969	
		P^{23}=0.0039***			P^{56}=0.1936	
		P^{13}=0.0029***			P^{46}=0.0419**	

Notes:
*, **, *** are significant at the level of 10%, 5% and 1% respectively.

S5 Table. Robustness checks of fertilizer input and fertilizer input areas.

	ADL		No. of diseases	
	(1)	(2)	(3)	(4)
Fertilizer input*Time	0.0010***		0.0001*	
	(0.0003)		(0.0001)	
Higharea*Time		0.2168***		0.0061
		(0.0547)		(0.0148)
Control	YES	YES	YES	YES
Year FE	YES	YES	YES	YES
ID FE	YES	YES	YES	YES
Observations	32443	32278	32443	32443
R-squared	0.6905	0.5646	0.6905	0.6903

Notes:
*, **, *** are significant at the level of 10%, 5% and 1% respectively.

S6 Table. Additional robustness checks of fertilizer loss in different areas.

	Major Agri-producing areas		Major Rice-producing areas		Water resource rich areas	
	ADL index	No. of diseases	ADL index	No. of diseases	ADL index	No. of diseases
Fertilizerloss*Time	0.0283***	0.00200	0.0203***	0.00500***	0.0274	0.0161***

	(0.0082)	(0.0021)	(0.0066)	(0.0017)	(0.0175)	(0.0043)
Control Variables	YES	YES	YES	YES	YES	YES
Year FE	YES	YES	YES	YES	YES	YES
ID FE	YES	YES	YES	YES	YES	YES
Observations	16880	16959	21528	21603	20526	20604
R-squared	0.572	0.695	0.549	0.677	0.533	0.667

Notes:
*, **, *** are significant at the level of 10%, 5% and 1% respectively.

S7 Table. Additional robustness checks of fertilizer loss areas in different areas in different areas.

	Major Agri-producing areas		Major Rice-producing areas		Water resource rich areas	
	ADL index	No. of diseases	ADL index	No. of diseases	ADL index	No. of diseases
Higharea*Time	0.255***	0.0389**	0.163***	0.0452***	0.144**	0.0339**
	(0.0677)	(0.0171)	(0.0567)	(0.0141)	(0.0613)	(0.0158)
Control Variables	YES	YES	YES	YES	YES	YES
Year FE	YES	YES	YES	YES	YES	YES
ID FE	YES	YES	YES	YES	YES	YES
Observations	16880	16959	21528	21603	20526	20604
R-squared	0.571	0.695	0.549	0.677	0.532	0.667

Notes:
*, **, *** are significant at the level of 10%, 5% and 1% respectively.

1. The fertilizer loss is continuous variable, and we dichotomized fertilizer loss by its pre-policy mean to compare the health effect in different regions. The average value of fertilizer loss is the national average of fertilizer loss; it is equal to 5.36kg/ha. High loss areas include Anhui, Shandong, Jiangsu, Guangdong, Hebei, Henan, Zhejiang, Hubei and Fujian, while low loss areas include Chongqing, Sichuan, Shaanxi, Guangxi, Hunan, Jiangxi, Heilongjiang, Jilin, Liaoning and Shanxi. In this paper, data from Beijing, Tianjin and Shanghai are deleted because of the small sample size and the small agricultural planting area.

2. Data source of loss coefficient: technical report of the first general survey of pollution sources.

3. The major agricultural-producing areas are Hebei Province, Inner Mongolia Autonomous Region, Heilongjiang Province, Jiangsu Province, Anhui Province, Shandong Province, Henan Province, Hubei Province, Hunan Province and Sichuan Province.

The major rice-producing areas are Heilongjiang Province, Jiangsu Province, Anhui Province, Jiangxi Province, Hubei Province, Hunan Province, Guangdong Province, Guangxi Zhuang Autonomous Region, Chongqing and Sichuan Province.

The water resource rich areas are Heilongjiang Province, Zhejiang Province, Anhui Province, Fujian Province, Jiangxi Province, Hubei Province, Hunan Province, Guangdong Province, Guangxi Zhuang Autonomous Region and Sichuan Province.

 Click here to access/download;Figure;Figures.docx

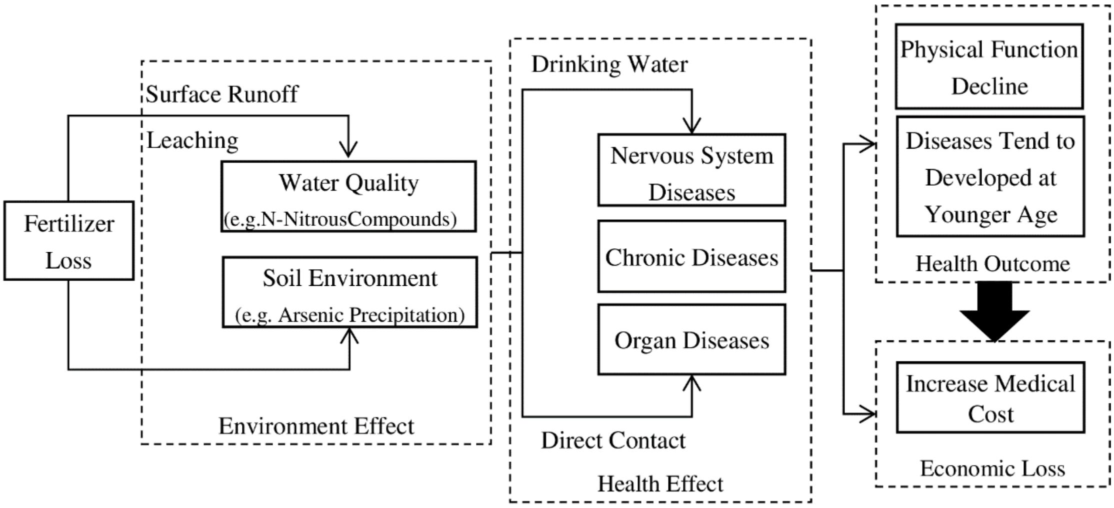

Fig1. Effects of chemical fertilizer loss on health.

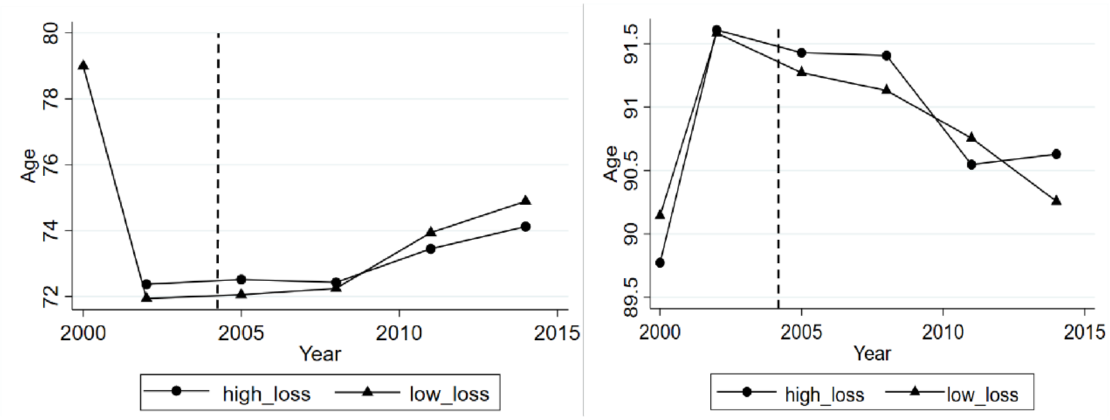

Fig 2. Chemical fertilizer loss and age of onset.

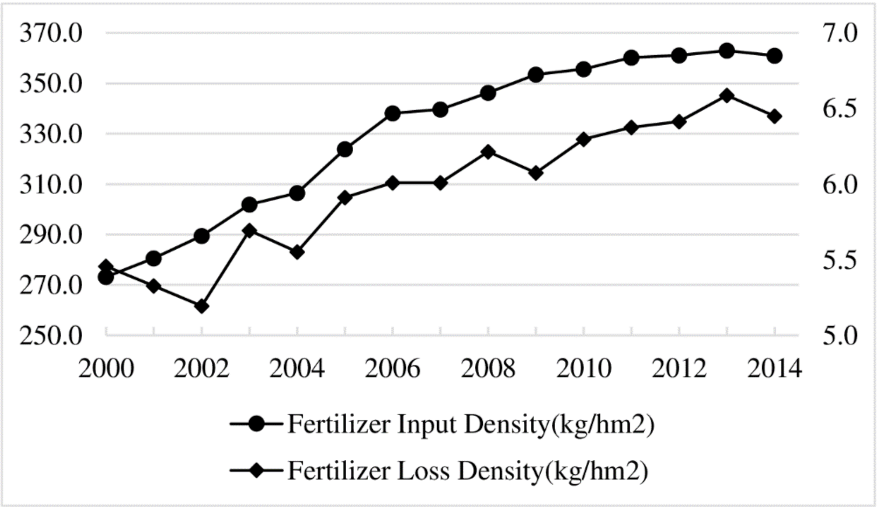

S1 Fig. Fertilizer input and fertilizer loss in China.

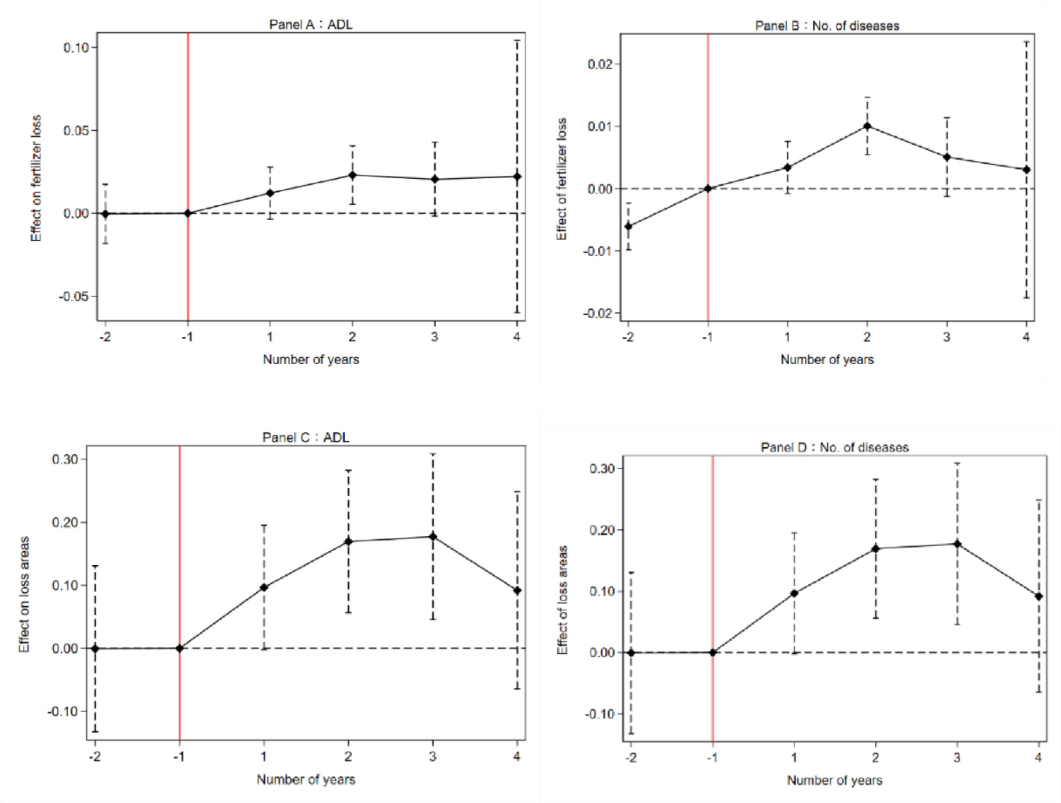

S2 Fig. Parallel trend test.

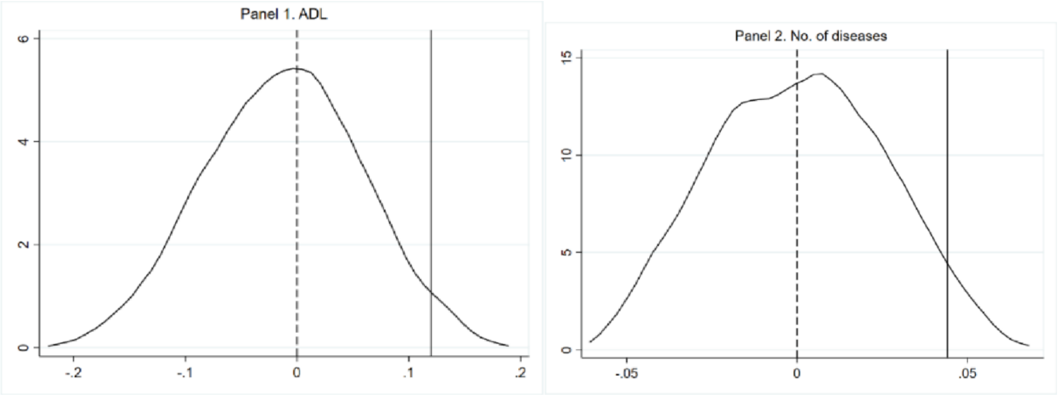

S3 Fig. Placebo test.

Notes:

Panel 1 is the result of the ADL index placebo test; Panel 2 is the result of the placebo test of the number of patients.

The X-axis represents the estimated coefficients from 1000 random assignments.

The curve is the estimated kernel density distribution. The vertical type is a true estimate in columns (2) and (4) of Table 3.

Figure1 Click here to access/download;Figure;Figures_figure 1.tif

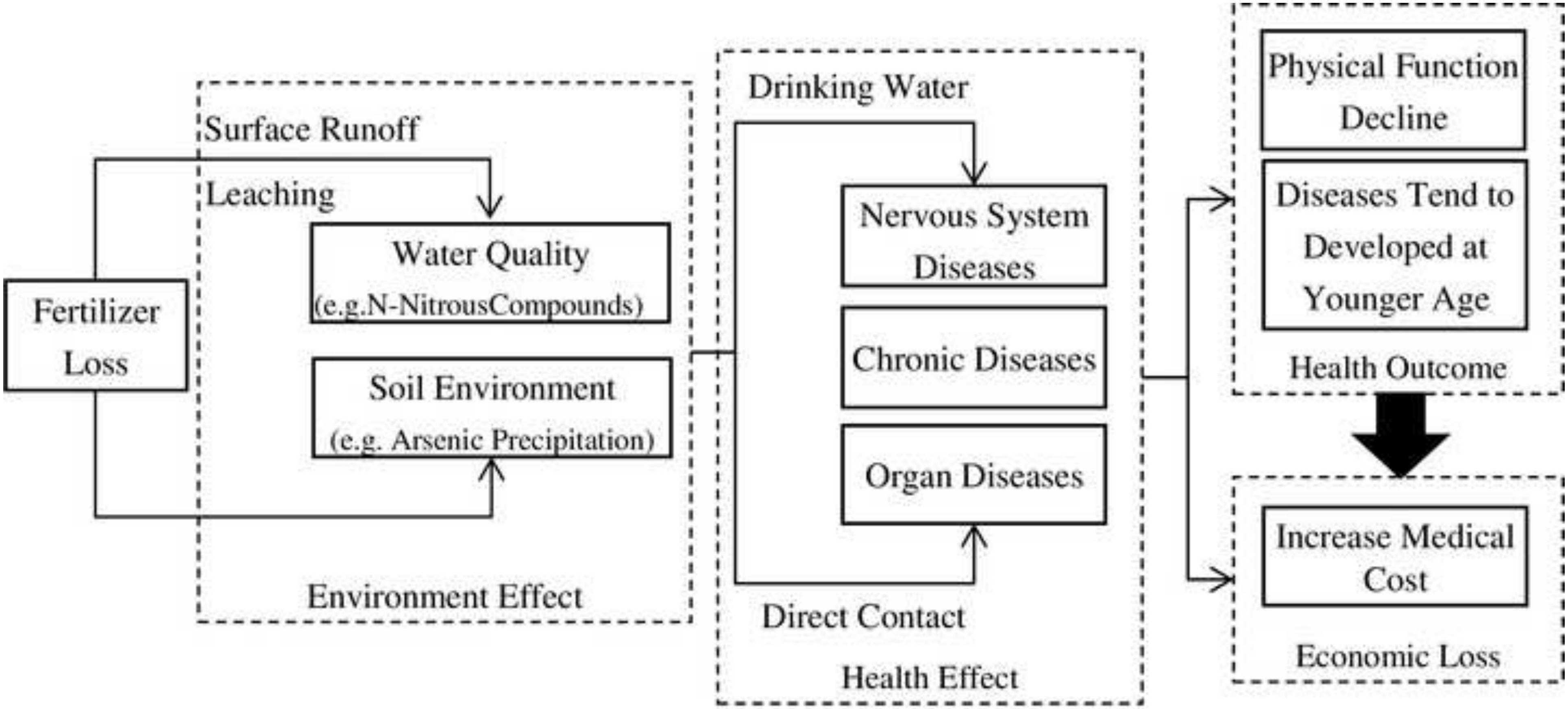

Fig1. Effects of chemical fertilizer loss on health.

Figure2 Click here to access/download;Figure;Figures_figure 2.tif

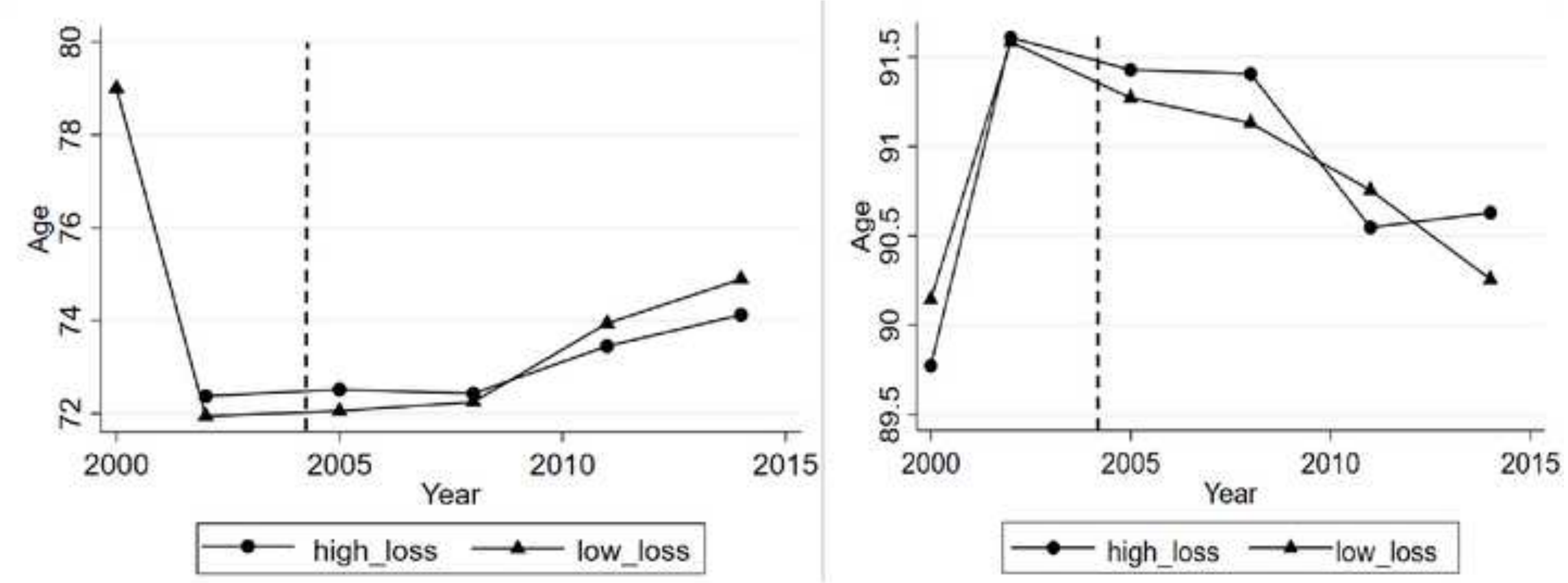

Fig 2. Chemical fertilizer loss and age of onset.

 Click here to access/download;Figure;Figures_S1 figure.tif

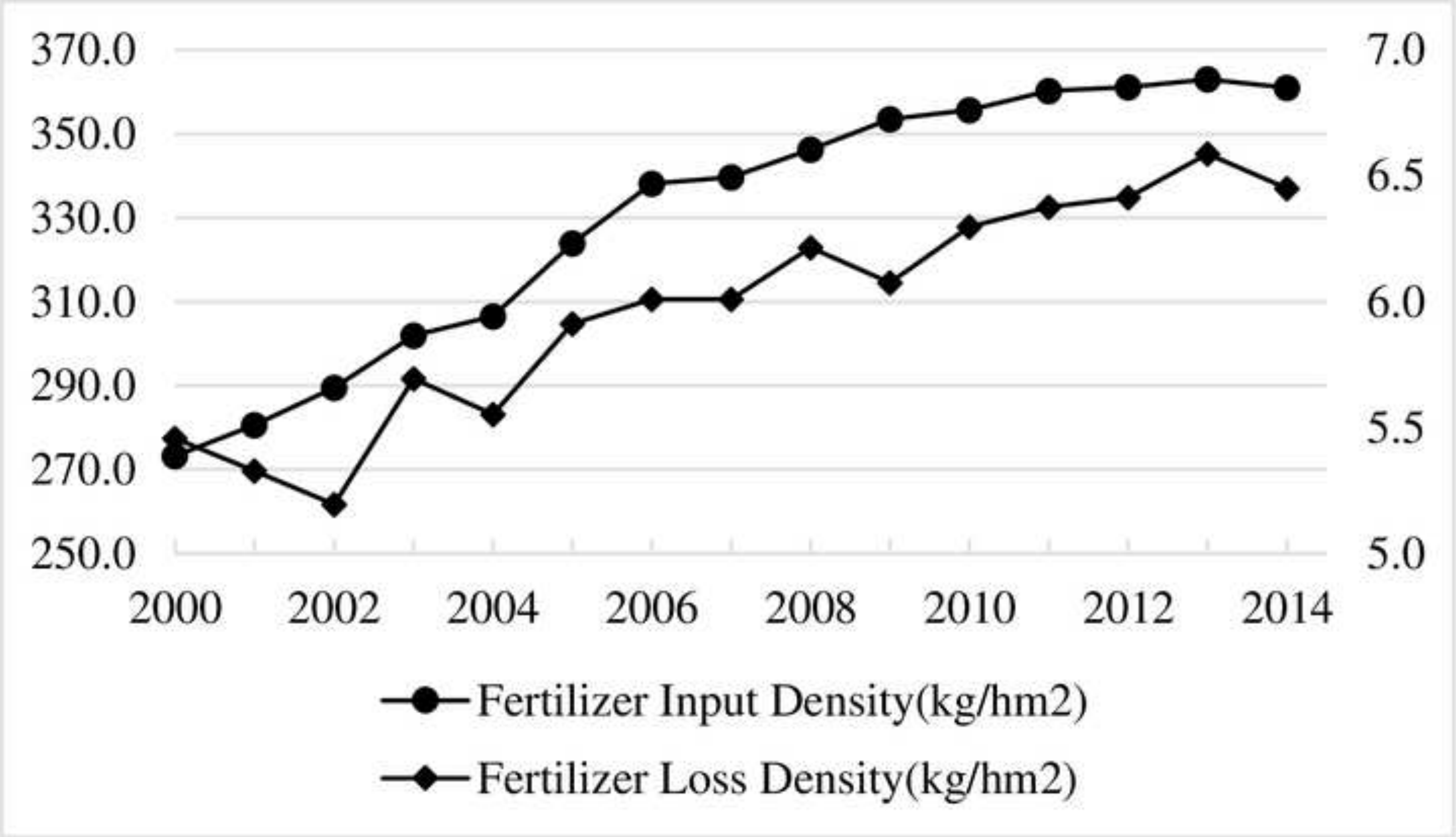

S1 Fig. Fertilizer input and fertilizer loss in China.

s2 Figure

Click here to access/download;Figure;Figures_S2 figure.tif

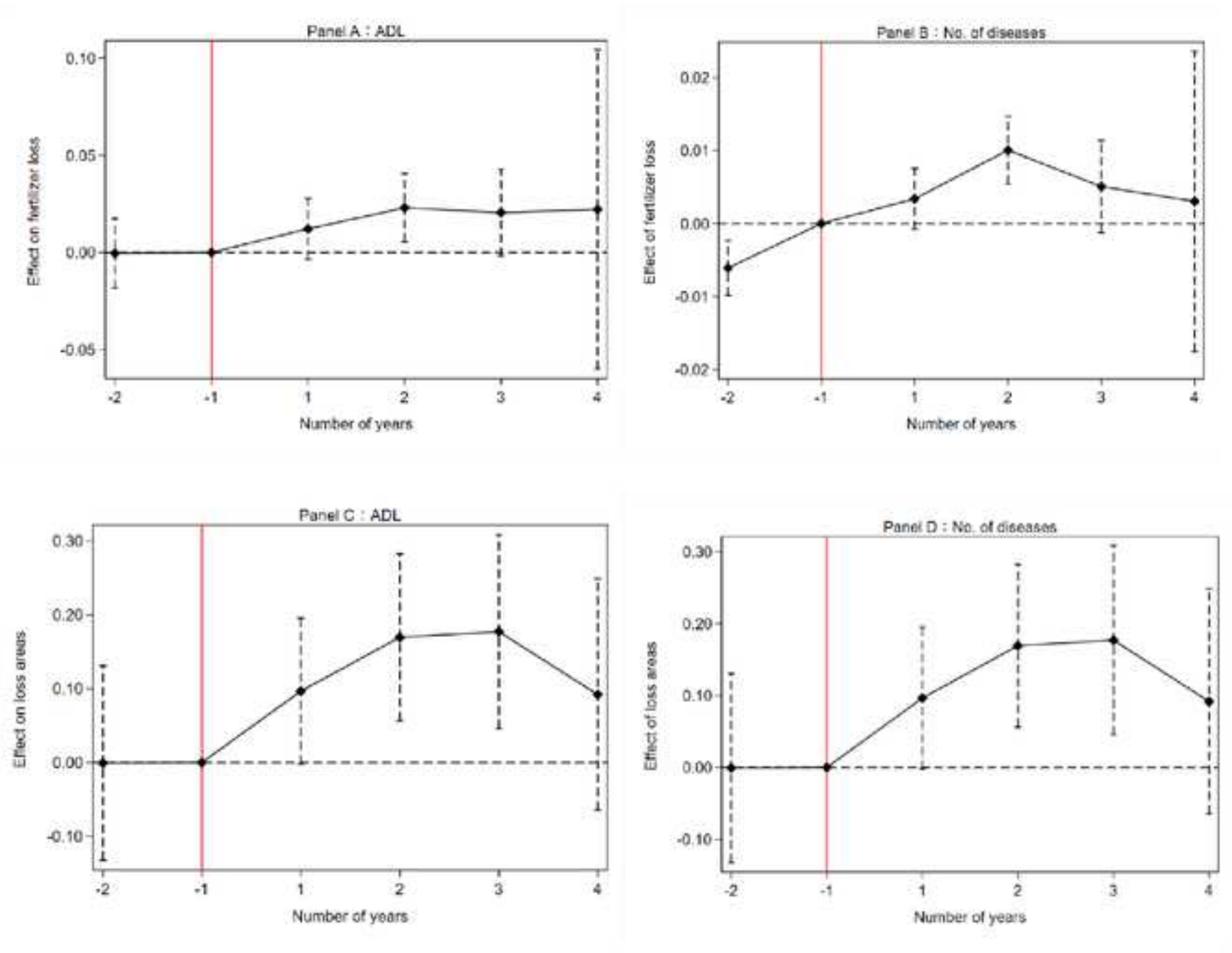

S2 Fig. Parallel trend test.

 Click here to access/download;Figure;Figures_S3 figure.tif

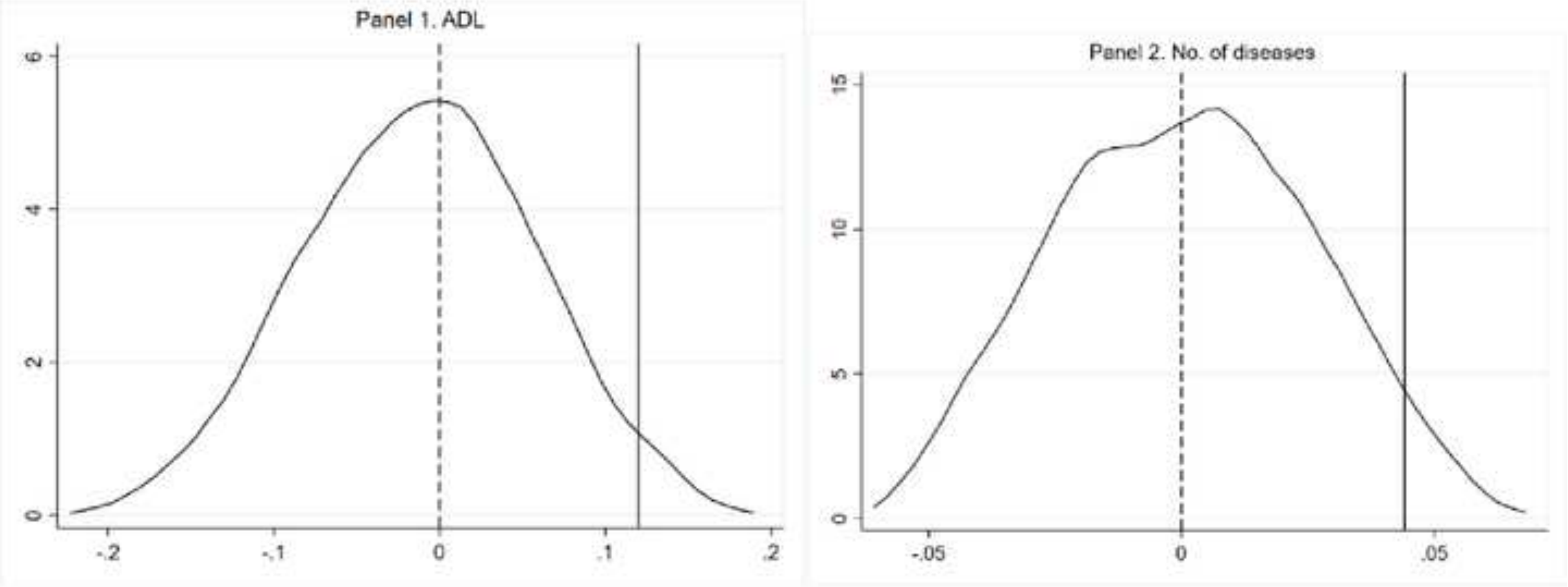

S3 Fig. Placebo test.

Agricultural Nonpoint-source Pollution and Health of the Elderly in Rural China

Ying WANG[1], Hang XIONG[2], Chao CHEN[3*]

[1] College of Economics and Management, Nanjing Agricultural University, Nanjing, Jiangsu, P.R.China

[2] College of Economics and Management, Nanjing Agricultural University, Nanjing, Jiangsu, P.R.China; China Center for Food Security Studies, Nanjing Agricultural University, Nanjing, Jiangsu, P.R.China

[3] College of Economics and Management, Nanjing Agricultural University, Nanjing, Jiangsu, P.R.China; China Center for Food Security Studies, Nanjing Agricultural University, Nanjing, Jiangsu, P.R.China

* Corresponding author

E-mail: cchen@njau.edu.cn.

Funding Information：This article was sponsored by the Priority Academic Program Development of Jiangsu Higher Education Institutions (PAPD)

Abstract

Large input and high loss of chemical fertilizer are the main causes of agricultural nonpoint-source pollution in China. Employing fertilizer loss and micro-health data, this paper analyzes the effects of chemical fertilizer loss on the health of rural elderly and the medical cost in China. Results of the difference-in-differences (DID) method indicate that one kg/ha increase in fertilizer loss alters a key medical disability index (Activities of Daily Living) by 0.0147(0.2 percent changes) and the number of diseases by 0.0057 for rural residents of 65 and older. This is equivalent to CNY 316 million (USD 45 million) in national medical cost. Furthermore, the age of onset is younger in regions with higher fertilizer loss. One kg/ha increase of fertilizer loss advances the age of onset by 0.267 year, which will cause long term effect on public health. Our results are robust to a variety of robustness checks.

1. Introduction

China experienced rapid growth in chemical fertilizer use, particular after 2004 when the Chinese government shifted from taxing agriculture to subsidizing agricultural programs. The unit area chemical fertilizer usage is nowadays 4 times more than the world average [1]. Agricultural nonpoint-source pollution is a growing concern in China because these pollutions had become the main cause of water pollution [2]. Agricultural nonpoint-source pollution has also been found to be an important factor affecting health [3-6]. Specifically, the elderly might have a higher health risk comparing with the young people when facing environmental exposure. A higher susceptibility and higher mortality rates could be observed among the adults over the age of 75 years compared with the younger ages [7-9]. The differences of health risk might due to physiological changes associated with aging [10]; the age-related behavior trends varied between younger and older adults which might affect older adults' exposures [11]; other factors such as socio-economic and nutritional status [12]. China had the most serious aging problems because~~,~~ 70% of elderly lives in rural areas

[13].~~,~~ T~~t~~he elderly residents might most be affected by agricultural nonpoint pollution. Another reason we focus on the rural elderly is that the health status, medical facility and diseases awareness of rural elderly are not as good as those in cities [14-15]. This paper complements the existing literature by proving new evidence on the effects of agricultural non-agricultural pollution. Air pollution as a leading problem for public health was broadly studied in agricultural nonpoint pollutions [16-17]. The work~~s~~ most closely related to this paper was~~were~~ Brained and Menon (2014) [4], which assessed the impact of fertilizer runoff on infant and child in India.~~,~~ W~~w~~e complement their study by emphasizing another vulnerable group, i.e., older adults, who are more likely to stay in countryside in the process of urbanization and most directly affected by the loss of chemical fertilizer. Another work by~~is~~ Lai (2017) [5]~~, which~~ assessed the health effect of pesticide by using the pesticide cost. In order to eliminate the influence of price, we use the amount of chemical input and loss, which are more accurate. Though both chemical fertilizers and pesticides can~~could~~ cause water pollution, they work~~ed~~ in different ways, fertilizer are directly spread in the soil which is more likely to affect with surface and groundwater runoff. ~~Therefore, this study to explain the health effect of fertilizer loss is valuable.~~ This paper emphasizes on non-occupational fertilizer exposure~~, which is necessary~~ because the farmers who are~~'~~ ~~occupational risk via~~ directly ~~exposure~~exposure to ~~of~~ chemical fertilizer ~~was~~ are only a small proportion of the population and had been studied [18-20]. Many more population may be at risk because of agricultural externality.

The inefficient use of chemical fertilizer has caused serious nonpoint-source pollution. Fertilizers leaded to "groundwater contamination, eutrophication of fresh water and estuarine ecosystems, tropospheric pollution related to emissions of nitrogen oxides and ammonia gas, and accumulation of nitrous oxide" [21]. A 10% increase in the use of nitrogen fertilizer ~~(kg)~~ and phosphorus fertilizer~~(kg)~~ leaded to a 1.525% and 1.374% increase of the concentration of nitrogen ~~(mg/l)~~ and phosphorus ~~(mg/l)~~ in water, respectively [22]. In China, agricultural nonpoint-source pollution has exceeded industrial pollution in water~~,~~. T~~t~~he chemical oxygen demand (COD) emission of agricultural pollution sources was 1.8 times of the industrial sources [23]. After 2004, in particular, China abolished agricultural tax and issued a series of agricultural support policies to promote agricultural production, which encouraged the excessive application of agricultural fertilizer. From

2004 to 2015, the amount of chemical fertilizer applications was increased by 30% (i.e., 1.386 million tons) [24], while the increase in agricultural produce output was far less. As a result of the formation of nitrite compounds and the change in soil environmental conditions, chemical fertilizer loss may potentially lead to chronic diseases, nervous system diseases, and organ diseases [25-27], and eventually affect human health as well as medical cost (Fig 1). Amines and amides in chemical fertilizers and sewage could cause digestive tract diseases, esophageal cancer, liver cancer, stomach cancer, esophagus cancer, and other cancers [28-30]. The fertilizer, which were soluble in water and had stable properties, were transported by rivers over long distances and large ranges, and enter the human body via drinking water and other ways to synthesize nitrosamines, especially nitrosamines. Drinking water or eating vegetables with a high nitrate content may lead to thyroid cancer, hypertension, neural tube defects (NTDs) [31-32], and various chronic diseases, such as skin diseases, lung cancer, bladder cancer, and cardiovascular disease [33]. Based on the background of China's agricultural support policy, this paper verifies the effect of chemical fertilizer loss from the soil on the health of the rural elderly, and comprehensively analyzes the health damage from the perspectives of daily living ability, disease risk, and disease age onset. The economic cost of this health damage is further calculated. More specifically, 3 hypotheses are proposed as follow: (1) Fertilizer loss can cause physical damage, increase the health risk and alter the daily living ability of the elderly. (2) Because of the physical functio[illegible] lead the individualssam [illegible] medi[illegible]er in high-loss regions [illegible]er

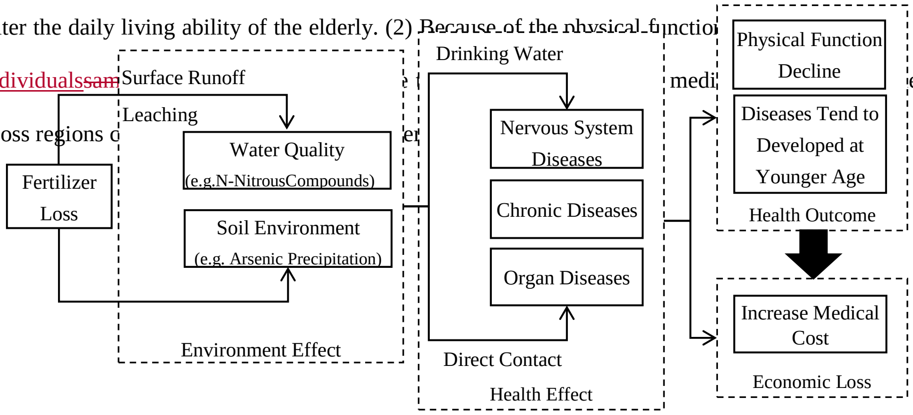

Fig1. Effects of ~~C~~chemical ~~Fertilizer~~ fertilizer ~~Loss~~ loss on ~~Health~~health.

The rest of the paper is organized as follows. Section 2 provides data description and summary statistics. Section 3 presents empirical models as well as results and Section 4 concludes.

2. Data and summary statistics

2.1 Data collection and variables

The analysis is accomplished using ~~difference~~ difference-~~in~~ in-differences method with the national dataset Chinese Longitudinal Healthy Longevity Survey (CLHLS) and publicly published yearbooks.

The individual health data come from the Chinese Longitudinal Healthy Longevity Survey (CLHLS). CLHLS is chosen for this research ~~owning~~ due to its richness in health and demographic information, its nationwide sampling areas, and its time span which covers the year of the agricultural policy change. The CLHLS was conducted in a randomly selected half of the counties and cities in 22 provinces, covering 85% of the total population in China. Its eight waves (1998, 2000, 2002, 2005, 2008, 2011,2014 and 2018) surveyed the cohort of 65 years and older. We only use the data from 2000 to 2014 because the survey question asking whether drinking water was boiled or not started in 2000; only those who drank boiled water are included in our analysis to eliminate possible health linkages to waterborne bacteria. China began to implement the "fertilizer reduction" policy in 2015.~~,~~ I~~i~~n order to eliminate th~~i~~ise policy effect, we did not use the data after 2015. The survey combines an in-home interview and a basic physical examination. Extensive information was collected on demographic characteristics, family and household characteristics, lifestyles, diet, psychological characteristics, health, disability, socioeconomic conditions, etc. Fertilizer input data come from the China Rural Statistical Yearbook (1999-2015) and other regional variables come from the China Statistical Yearbook (1999-2015). Chemical fertilizer loss index come from ~~the Technical report on China pollution source census~~ Data collection of the first national survey of pollution sources (2011).

2.1.1 Health outcome~~s~~ variables

Multiple indicators for health status of the elderly are examined linked to fertilizer loss in the extant medical and toxicology literatures, including the index of Activities of Daily Living (ADL), the numbers of diseases related to fertilizer loss, and age of onset.

The ADL index is widely used to measure the dependence of the elderly and physically inactive people. It is also known as the "disability" index [34]. The ADL index is constructed by asking participants whether they need help with six basic activities (namely bathing, dressing, eating, going to the toilet, indoor movement, and controlling defecation). The choices for each item are "can do without help" (corresponding score is 1), "need help" (corresponding score is 2), and "need full help" (corresponding score is 3). The final score of the ADL index is the sum of all six items, ranging from 6 to 18. Higher scores mean that the health status of the elderly is worse.

The number of diseases measures how many types of diseases related to fertilizer loss are suffered by a sample individual. Although the CLHLS database investigated 16 diseases in the elderly, including hypertension, heart disease, cancer, stroke, cardiovascular and cerebrovascular diseases, bronchitis, emphysema, asthma, and pneumonia, only three diseases—hypertension, cancer, and gastrointestinal ulcer—were related to fertilizer loss in the existing medical literature.

The age of onset refers to the age at which a sample individual first suffered from any of the three diseases (i.e., hypertension, gastrointestinal ulcer, or cancer).

2.1.2 Individual and household characteristic variables

Individual and household characteristic variables include information on socio-economic characteristics, health behavior and dietary pattern. Socio-economic characteristics include age, gender, people living together, income station, basic health condition and hospitalization. Health behaviors measure whether the respondents smoke, drink or do any exercise (with options of "Yes" or "No"). Dietary patterns measure the frequency with which respondents eat meat, fish, egg, and salt-preserved vegetables. Options for each item are "rarely or never", "not every month, but occasionally", "not every week, but at least once per month", "not every day, but at least once per week", "almost every day", with scores from 1 to 5, respectively. The questionnaire also

asks the sources of respondents' drinking water. Options include drinking water sources from a well, a river of lake, a spring, a pond or pool, and tap water. A binary variable (Water) is constructed, which equals one if respondents drink surface water, i.e. water from a rive or lake or a pond of pool, and equals zero otherwise.

2.1.3 Provincial level variables

Provincial level variables include fertilizer loss, fertilizer use, organic fertilizer use, pesticide use, industrial pollution index, the numbers of hospital and provincial Gross Domestic Product (GDP). The calculation method for fertilizer loss intensity is based on literature [35]. On the basis of the unit investigation method and the inventory analysis method, the fertilizer loss coefficient ~~method~~ is used to calculate the total nitrogen and phosphorus emissions; then the total emission and emission intensity of fertilizer nonpoint-source pollution are calculated. The calculation formula is as follows:

$$E=\sum E_{ij}=\sum C_{ij}\times\eta_{ij} \qquad (1)$$

$$I=E/A \qquad (2)$$

where E measures the total emission of fertilizer loss; E_{ij} measures the amount of the pollutant j produced and the loss into water by province~~pollutant~~ i; C_i measures the index statistics of pollutant i (compound fertilizer is converted into total nitrogen 40% and phosphorus pentoxide 32%); η_{ij} measures the loss coefficient; A represents the crop areas; and I represents pollution emission per unit area, i.e., pollution intensity (unit: kg/ha).

The industrial pollution index ($Indpollution_i$) controls industrial activities, which is defined in Equation (3) according to existing literature [36-37]. GDP measures general economy and wealth conditions. The number of hospitals controls the level of medical service. Because the health data are observed once three years, all provincial variables are assigned the average of the last three years.

$$Indpollution_i=\frac{pv_{i1}+pv_{i2}+pv_{i3}}{3},\quad pv_{ij}=p_{ij}\Big/\sum_{i=1}^{n}\frac{p_{ij}}{n} \qquad (3)$$

where p_{ij} measures the pollutant j of province i, n is the ~~amount~~ total number of provinces, pv_{ij} measures the emission index of pollutant j in the province i relative to the national average level. The larger

value means the higher pollution level. $pv_{i1}, pv_{i2}, pv_{i3}$ represents industrial wastewater, industrial sulfur dioxide and industrial dust, respectively.

2.2 Statistical analyses

2.2.1 Sample summary

The statistical descriptions of all variables used in the regression are summarized in Table 1.

Table 1. Variable description and summary statistics.

Variables	Description	Obs.	Mean	Std.Dev.	Min	Max
**Household Variables**						
Age	Age in years	39093	85.83	10.83	65	109
Male	Male=1;Female=0	39093	0.44	0.50	0	1
Co-residence	Nursing home=1; Alone or Spouse=2; Child=3; Others=4	39093	2.62	0.57	1	4
Income_cost	If income support daily cost (Yes=1; No=0)	39093	0.78	0.41	0	1
Illness	Number of 15 chronic diseases	39093	0.85	1.06	0	1
Hospitalization	The times of hospitalization in two years	39093	0.26	0.79	0	30
**Health Behaviors**						
Smoke	Currently smoke (Yes=1; No=0)	39093	0.33	0.47	0	1
Drink	Currently drink (Yes=1; No=0)	39093	0.31	0.46	0	1
Exercise	Currently exercise (Yes=1; No=0)	39093	0.27	0.44	0	1
Dietary Pattern						
Meat	The frequency of meat consumption	39093	3.95	1.03	1	5
Fish	The frequency of fish consumption	39093	3.36	1.15	1	5
Egg	The frequency of egg consumption	39093	3.92	1.07	1	5
Salt_vege	The frequency of salt-preserved vegetable consumption	39093	3.27	1.38	1	5
Boiled water	If drink boiled water (boild water=0; not boild water=1)	39093	0.057	0.23	0	1
Water	Drirking water source (Surface water=1; Tap water=0)	39093	0.14	0.35	0	1
**Provincial Variables**						
Floss	Fertilizer loss intensity (kg/ha/year)	39093	7.34	3.76	0.60	14.82
Finput	Fertilizer input intensity (kg/ha/year)	39093	354.80	92.47	128.41	704.32
Pinput	Pesticide inptut intensity (kg/ha/year)	39093	12.62	6.56	2.28	22.26
OFinput	Organic fertilizer input intensity (kg/ha/year)	39093	286.53	361.18	11.56	1997.00
Hospnum	The number of hospital per province (million)	39093	0.02	0.02	0.01	0.08
LnGDP	Logarithm of Provincial Gross Domestic Product	39093	9.10	0.91	7.32	11.04
Indpolltion	Index of industrial pollution (lower=better)	39093	1.46	0.55	0.45	2.95
**Health indicatos**						
ADL	Activities of Darly Living score (lower=better)	39093	6.66	1.80	6	18
No. of diseases	The number of illness caused by fertilizer loss (lower=better)	39093	0.25	0.48	0	3
Age_ill	Age of suffering from illness caused by fertilzer loss (lower=worse)	9503	83.70	10.06	65	109

Mescost	Medical cost(RMB)	39093	2768.29	11676.91	0	199996

2.2.2 Fertilizer loss and health outcome after agricultural support policies

China shifted from taxing agriculture to subsidizing agricultural programs since 2004 to maintain food security and self-reliance. In 2004, authorities introduced three subsides targeted at grain producers: a direct payment for grain producers, a subsidy for improved seed varieties and a partial rebate for farm machinery purchases. Several other support programs were introduced such as a general-input subsidy, price floors for wheat and rice, reform of the grain marketing system and transfer payment to grain counties since 2004, which has been accompanied with greater fertilizer use and fertilizer loss. This set of supporting programs had bolstered farmer' production incentives and crop production increased continuously since 2004 [38-39], on the other hand, the programs might lead to the distortion of agricultural factors and cause ~~the~~ negative effects on~~of~~ soil and water environment [40]. Table 2 reports the differences of health outcomes and fertilizer loss between regions with high versus low intensity of fertilizer loss. We report the differences of ADL index, the numbers of induces diseases, intensity of fertilizer input, amount of fertilizer loss and intensity of fertilizer loss before the implementation of agricultural support policies in column (3). The differences after the agricultural support policies are in column (6). The widening gap in health outcomes and fertilizer loss and input can be observed in column (7) between regions with high and low fertilizer loss after the agricultural support policies. Preliminary data analysis shows that the loss of chemical fertilizer in high-loss areas was more serious after the agricultural subsidy policies.

Table 2. Comparison of high-loss-areas and low-loss areas by agricultural support policies.

Variables	Before agricultural Support Policies			After Agricultural Support Policies			2nd difference (7)=(6)-(3)
	Low loss (1)	High loss (2)	1st difference (3)=(2)-(1)	Low loss (4)	High loss (5)	1st difference (6)=(5)-(4)	
ADL index	6.823	7.022	0.199	6.822	7.108	0.287	0.088
No. of diseases	0.160	0.211	0.051	0.255	0.340	0.085	0.035
Input intensity (kg/ha)	239.562	358.413	118.851	317.519	457.163	139.644	20.793
Total loss (10000 tons)	1.689	6.717	5.028	2.023	7.350	5.327	0.299
Loss intensity (kg/ha)	2.905	8.599	5.694	3.491	9.226	5.735	0.041

Notes:
The fertilizer loss is continuous variable, and we dichotomized fertilizer loss by its pre-policy mean to compare the health effect in different regions. The average value of fertilizer loss is the national average of fertilizer loss; it is equal to 5.36kg/ha.
We showed the fertilizer input and fertilizer loss in S1 Fig.

2.2.3 Age of onset and fertilizer loss

The CLHLS contains three diseases (namely hypertension, gastrointestinal ulcer and cancer) induced by fertilizer loss. The age, when one sample first suffers from any of the three diseases, is defined as age of onset. The age of onset comparisons across age groups are represented by Fig 2. A significant difference of age of onset can be observed between the regions with high versus low intensity of fertilizer loss in younger-old groups (< 80 years) in Panel A of Fig ~~3~~2. The age of onset is not significantly different in the oldest-old samples.

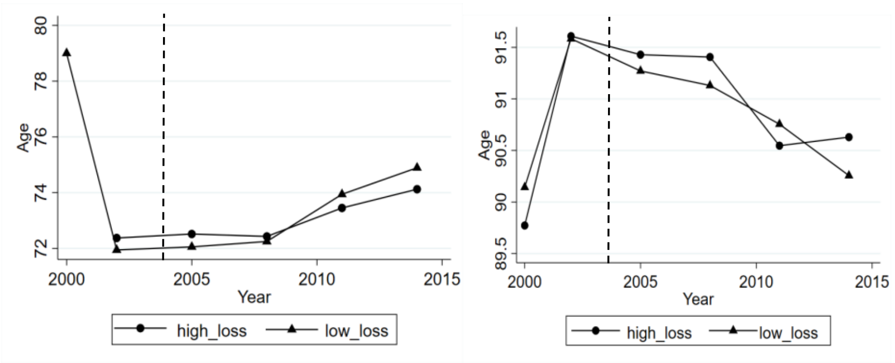

Fig 2. Chemical ~~f~~Fertilizer ~~Loss~~ loss and ~~Age~~ age of ~~Onset~~onset.

3. Empirical models and results

This section investigates the health effect of fertilizer loss on local elderly population. Preliminary data analysis reports the health outcomes are different ~~the differences~~ in regions with high verse low intensity of fertilizer loss after agricultural subside policies in 2004. Further, we follow a difference-in-differences (DID) framework to compare health outcomes between people in regions with different intensities of fertilizer loss before and after 2004, when China shifted from taxing agricultural outputs to subsidizing agriculture. The medical cost is~~are~~ estimated as well. To facilitate comparisons across age groups, we explore heterogeneous effects by splitting the sample in various age groups and reporting estimates of α_1 and β_1 for these subsamples. Then several robustness checks are conducted to help assess the validity of our results. The difference-in-differences model is specified as:

$$Health_{it} = \alpha_0 + \alpha_1 Fertilizerloss_{it} \times Time_{it} + \alpha_2 Fertilizerloss_{it} + \alpha_3 Time_{it} + \alpha_4 X_{it} + \gamma_t + \mu_i + \varepsilon_{it} \quad (4)$$

$$Health_{it} = \beta_0 + \beta_1 Area_{it} \times Time_{it} + \beta_2 Area_{it} + \beta_3 Time_{it} + \beta_4 X_{it} + \gamma_t + \mu_i + \varepsilon_{it} \quad (5)$$

$$Age_{it} = \delta_0 + \delta_1 Fertilizerloss_{it} \times Time_{it} + \delta_2 Fertilizerloss_{it} + \delta_3 Time_{it} + \delta_4 X_{it} + \gamma_t + \mu_i + \varepsilon_{it} \quad (6)$$

$$Age_{it} = \theta_0 + \theta_1 Area_{it} \times Time_{it} + \theta_2 Area_{it} + \theta_3 Time_{it} + \theta_4 X_{it} + \gamma_t + \mu_i + \varepsilon_{it} \quad (7)$$

where $Health_{it}$ denotes the health outcome; Age_{it} denotes the age of ~~-~~onset; α_1, β_1, δ_1 and θ_1 identify the effects of fertilizer loss; $Fertilizerloss_{it}$ denotes the fertilizer loss of the region where individual~~s~~ i resides; $Area_{it}$ is a dummy variable equaling 1 if the individual is in high loss areas, and 0 otherwise; $Time_{it}$ equals 1 starting in 2004 and 0 otherwise; X_{it} is a vector of control variables including individual, household, and provincial characteristics such as age, gender, health behavior, dietary habits, industrial pollution and hospitals, etc.; μ_i is a fixed effect unique to individuals i; γ_t is a time effect common to all individuals in year t; and ε_{it} is an error term.

The relationship between medical cost and health can be identified in the following Equation:

$$Y_{it} = \alpha_0 + \alpha_1 ADL_{it} + \alpha_2 X_{it} + \gamma_t + \mu_i + \varepsilon_{it} \quad (8)$$

where Y_{it} represents medical costs, and other variables are ~~as~~ defined as before.

3.1 Basic regression results of health

3.1.1 Effect of fertilizer loss on health

Table 3 reports the α_1 and β_1 coefficients in Equation (4) and Equation (5) for outcomes related to ADL index and No. of diseases. Estimated coefficients in column (1) of Table3 indicate that one kg/ha increase of chemical fertilizer loss is associated with increased ADL daily life index of 0.0147(0.2 percent). From column (4) one kg/ha increase of fertilizer loss is associated with 0.0057 raise in the number of diseases (0.5 percent). These results indicate that the fertilizer loss increase the elderly's health risks and support diseases significantly. ~~-~~For different regions, estimates for β_1 in columns (2) and column (5) of Table 3 indicate a reduction in the ADL index and the number of diseases for high-loss area verse low-loss areas. Compared with low-loss areas, the ADL index of the rural elderly is 0.1222 higher in areas with high chemical fertilizer

loss, while the number of diseases is 0.0487 higher. We also use the two-way fixed effects model to estimate the health effect of fertilizer loss. Column (3) and Column (6) of Table 3 report the coefficients for outcomes related to ADL index and No. of diseases. Estimated coefficients indicate that one kg/ha of fertilizer loss increase is associated with increased ADL daily life index of 0.1088. ~~From c~~Column (6) shows that one kg/ha of fertilizer loss increase is associated with 0.0211 raise in the number of diseases. The health effect of agricultural fertilizer input not only accrue in farmers who are likely to receive a fertilizer loss via direct exposure. Many more proportions of the population are at risk of fertilizer loss because of exposure to contaminated water and soil. Agricultural support policies play an important role in the fertilizer loss, and the agricultural support policies may cause heterogeneous fertilizer loss between regions because of the different subsides levels. These may cause the biased result of TWFE.~~,~~ DID model can avoid this problem, and we control the time effect and individual effect in our regressions.

Table 3. Basic ~~Estimates~~ estimates ADL index and ~~Diseases~~diseases.

	ADL			No. of diseases		
	DID		TWFE	DID		TWFE
	(1)	(2)	(3)	(4)	(5)	(6)
Fertilizerloss*Time	0.0147**	--	--	0.0057***	--	--
	(0.0060)	--	--	(0.0014)	--	--
Higharea*Time	--	0.1222***	--	--	0.0487***	--
	--	(0.0461)	--	--	(0.0114)	--
Fertilizer loss	--	--	0.1088**	--	--	0.0211*
	--	--	(0.0432)	--	--	(0.0126)
Control	YES	YES	YES	YES	YES	YES
Year FE	YES	YES	YES	YES	YES	YES
ID FE	YES	YES	YES	YES	YES	YES
Observations	32165	32165	38908	32329	32329	38680
R-squared	0.5570	0.5582	0.~~0843~~3183	0.6906	0.6907	0.3243

Notes:

~~[a] robust standard errors for clustering at the individual level are reported in brackets;~~ *, **, *** are significant at the level of 10%, 5% and 1% respectively.

The estimated results without control variables were in S1 Table.

Considering the nonlinear health factors, we further estimated the health effects of fertilizier loss using propensity score matching (PSM) difference-in-differences (DID) model. The results were in S2 Table.

Formatted: Indent: First line: 0 ch

3.1.2 Heterogeneous health effects

In ~~S1~~ S3 and ~~S2~~ S4 Tables, we explore the heterogeneous effects by splitting the samples in different age groups and reporting estimates of α_1 for these subsamples. First, the oldest olds (≥80years) were discussed in

previous studies [41], because the oldest olds had ~~the~~ greater risk of falling and hospitalization [42-44]. We divide the samples at age into the younger age samples (<80years) and the oldest olds samples (≥80 years). In Columns (1) and columns (2) of ~~S1~~ S3 Table, a positive and significant~~ly~~ estimate of α_1 can be observed in the oldest olds. The estimates of α_1 are positive and significantly for both age groups (columns (3) and columns (4) of ~~S1~~ S3 Table).~~,~~ T~~t~~he fertilizer loss will increase the risk of diseases in both the younger olds and the oldest olds. The grouped estimates for effect of fertilizer loss on ADL and diseases of the oldest old are significantly distinguishable from the younger olds at the 1% level. We also divide the samples into younger olds (65years-75years), middle olds (75-85years) and the oldest olds (≥85years) [45-47]. The differences between younger and middle olds are not significant in both ADL and No. of diseases. The coefficient for oldest olds is larger than that for younger and middle olds, and the differences are significant.

3.1.3 Effect of fertilizer loss on ~~Age~~ age of ~~Onset~~onset

~~Table 4 reports the δ_1 and θ_1 coefficients in Equation (6) and Equation (7) for outcomes related to age of onset. Fertilizer loss increases the risk of disease in the rural elderly.~~ This study further explores whether fertilizer loss will lead to the elderly be sick in younger age. Though from Fig 2, the age of onset of elderly in areas with high-loss of chemical fertilizer are younger, in order to understand the relationship between fertilizer loss and the age of onset, we use the difference-in-differences model for empirical analysis. Estimated coefficient in column (1) of Table 4 indicates that one kg/ha increase of fertilizer loss advances the age of onset by 0.2670 year. ~~Comparing~~ Compared with low-loss areas, the age of onset ~~is~~ decline~~ds~~ by 0.3753 in high-loss areas.

Table 4. Estimation of fertilizer loss on age of onset.

	Age of Onset	
	(1)	(2)
Fertilizerloss*Time	-0.2670*<br>(0. 0161)	
Higharea*Time		- 0.3753**<br>(0. 1258)
Control Variables	YES	YES
Year FE	YES	YES
ID~~ndividual~~ FE	YES	YES
Observations	9503	9503

R-squared	0.9822	0.9822

Notes:
a *, **, *** are significant at the level of 10%, 5% and 1% respectively.

3.2 Results of health conditions on medical cost

In this section, we further continue explore these results by translating estimated effects into expected monetary losses. We do this by exploiting the relation between ADL and medical cost in Equation (8). Estimated coefficient in column (1) of Table 5 indicates that each additional unit increase in ADL index increases the medical cost by CNY 244.6 (USD 34.94). Columns (2) and (3) report the λ_1 coefficient in Equation (8) in high-loss and low-loss areas, respectively. The estimates indicate that there is a significant difference in medical cost between high loss areas and low loss areas., Eevery one-unit ADL increase is associated with a higherthe medical cost of CNY 194.64 (USD 27.81) higher in high loss areas. The health cost per unit loss of chemical fertilizer (1kg/ha) is about CNY 3.64 (USD 0.51). According to the sixth census, there were about 87.91 million rural elderly people (over 65 years of age) in China, and the loss of chemical fertilizer per unit will add medical costs by CNY 316 (USD 45) million. Though agro-chemical use is associated with significantly greater agricultural output value a range studies showed that agro-chemical also damaged human health. Indirectly, medical cost increases and labor supply loss could be observed due to illness [48]. Similar to our study, Lai suggested that a 10%(CNY 0.2/USD 0.03) increase in rice pesticide use will add 168.8 and 55.89 million dollars to medical costs and offspring's human capital losses, respectively [6]. According to the sixth national census, there were about 87.91 million rural elderly people (over 65 years of age) in China. Compared with the pesticide use, we We estimate the economic loss of fertilizer loss, an increase of one 1kg/ha of fertilizer loss and fertilizer input will add CNY 316 (USD 45) million and CNY 21.5 (USD 3.1) million to national medical cost, respectively.

Table 5. Effect of health on medical cost.

	Medical Cost		
	Overall samples	High-loss area	Low-loss area
ADL index	244.60***	331.94***	137.30**
	(55.81)	(93.54)	(63.58)
Control Variables	YES	YES	YES
Year FE	YES	YES	YES
Individual ID FE	YES	YES	YES
Observations	23087	19875	15167
R-squared	0.0709	0.0226	0.0121
p		0.0853*	

Notes:
*, **, *** are significant at the level of 10%, 5% and 1% respectively.
~~*, **, *** are significant at the level of 10%, 5% and 1% respectively.~~

3.3 Robustness check

3.3.1 Parallel trend test

In order to test ~~that~~ whether the time staggered entry events of agricultural policy are effective exogenous shocks, ~~this study makes~~we conduct a parallel test~~. We~~ base on Equation (9):

$$H_{it} = \alpha + \sum_{k\geq -2, k\neq -1}^{k=4} \delta_k D_{it,k} \times Fertilizerloss_{it,k} + \lambda X_{it} + \gamma_t + \mu_i + \varepsilon_{it} \quad (9)$$

where $D_{it,k}$ is a binary variable that takes value of 1 when agricultural support policies are implemented. We use $k=-1$ to denote the base year. S2 Fig, Panel A and Panel B plots estimates of ADL index and No. of diseased, respectively. The pre-period coefficients are statistically indistinguishable from zero and there is a sharp, statistically significant and sustained change after the policy implementation. The post-coefficients are significant. We also adopt a binary variable that takes value of 1 when the individuals in high loss areas. The estimates coefficients were shown in S2 Fig Panel C and Panel D.

~~divide the samples to five sections: the first period before the policy implementation, the second period after policy, the second two periods after the policy implementation to the end of sample period. Thus, five indicator variables pre_1, post_1, post_2, post_3 and post_4 are constructed. S1 Fig, Panel (1) plots estimates of ADL index and Panel plots estimates of No. of diseases. The set of pre_1 coefficient is statistically indistinguishable from zero, but there is a sharp, statistically significant and sustained change after the policy implementation.~~

3.3.2 Placebo test

In order to further test whether the results are driven by unobservable factors at~~of~~ the individual or ~~and~~ provincial level, referring to the methods of existing literature [49-50], this study conducted a placebo test by

Field Code Changed

Field Code Changed

Field Code Changed

randomly assigning high and low chemical fertilizer loss areas. Specifically, 9 out of 19 provinces are randomly selected as the experimental group, assuming that these 9 provinces are areas with high loss of chemical fertilizer, and other regions are the control group (areas with low loss of chemical fertilizer).~~ Random sampling ensures that the model constructed in this paper has no effect on the health level of the elderly.~~ In this study, random sampling is carried out 1000 times, and the benchmark regression is performed according to Equation (5). S3~~2~~ Fig reports the average value of the ADL index and the number of induced diseases after 1000 random distributions. The mean value of the 1000 random sample regressions is almost 0. The placebo test shows that our basic estimation results are unlikely to be driven by unobservable factors at the individual or~~and~~ provincial level.

3.3.3 Effect of fertilizer input on health

In order to verify the robustness of the research conclusions, the application intensity of chemical fertilizer and the areas with high and low chemical fertilizer application intensities are used to test the robustness. According to the criteria ~~construction index~~ of ecological counties in China (the application intensity of chemical fertilizer should not exceed 250 kg/ha), the areas with a chemical fertilizer application intensity exceeding 250 kg/ha are defined as high input areas, and the areas with a chemical fertilizer application intensity not exceeding 250 kg/ha are defined as low input areas. The basic regression of Equations (4) and (5) are carried out using the chemical fertilizer application intensity. The model results are shown in ~~S3~~ S5 Table. The model results are essentially consistent with the benchmark regression results, indicating that the research results are robust. For every 1 kg/ha increase in fertilizer input intensity, the ADL index will increase by 0.001 and the number of induced diseases will increase by 0.0001.

3.3.4 Effect in different areas

In order to verify the robustness of the ~~conclusions~~ analysis of this study, different samples are used~~ to test the robustness.~~ This paper focuses on health effect of agricultural fertilizer loss of the rural elderly. Therefore, samples from major agricultural-~~producing~~ production areas, major rice-producing areas and water

resource-rich areas are used to carry out the basic regression of Equation (4) and Equation (5). The results (S65 and S6 S7 Tables) show that fertilizer loss leads to a significant increase both on ADL index and the number of diseases in major agricultural-producing areas, major rice-producing areas and water resource-rich areas, which are basically the same comparing with the basic regression results in Ttable3. The basic regression results are robust to a variety of robustness checks.

4. Conclusions and discussion

China's agricultural fertilizer input is facing a problem of "high input, high loss, and high pollution", which hasd been aggravated by agricultural support policies since 2004. The problem of the "Three Highs" of chemical fertilizer use has caused pollution to the environment and health damages to, many more rural residents may be at health risk because of fertilizer loss. In this paper, the chemical fertilizer loss data and CLHLS micro-health data are used to verify the health effect of fertilizer loss of on the rural elderly in China. Nearly 60% elders live in rural areas in China had the most serious aging problem;and it was estimated that the number of the elders might increase to 450 million (nearly one third of the total population) by 2050. Nearly 60% elders live in rural areas [51]. The health decline caused by fertilizer loss not only increased the medical cost but also affected the economics, e.g. reducing labor time, increasing family care time and so on [52-53]. The health effect of fertilizer maybe a greater challenge to pension and health care in rural China.

To improve the health statusion of elderly in rural areas, the government can consider from agricultural policy and public health policy. (1) reduce fertilizer loss can be reduced by innovating agricultural production technology research and promoting technology adoptions. e.g. soil testing, formula fertilization technology, slow-release fertilizer technology, deep tillage machinery promotion, and straw returning technology. (2) as the loss of chemical fertilizer includes not only the loss from the current year, but also the basic loss of fertilizer, the government should improve soil quality to increase nutrient preserving capability of soil,, such as soil improvement technology, etc. (3) drinking water is an important way of fertilizer loss affect the public health, so the government need to focus on the rural drinking water, and to improve the rural elderly's awareness of drinking safety and the risk of health damages due toof chemical fertilizer loss.

The health effects of agricultural nonpoint-source pollution are estimated by taking the fertilizer as an example. Due to the limitations of the article space, this article does not discuss the health effect of other agricultural pollution, such as pesticides use, straw combustion and the interaction of various agricultural pollutions. The nonpoint-source pollution in animal husbandry and fishery is also worth ~~to~~ discussing.

References

1. NBSC. 2016. China Statistical Yearbook. Beijing, National Bureau of Statistics of China.
2. Zhou K, Fan J, Liu HC. 2017. Spatiotemporal patterns and driving forces of water pollutant discharge in the Bohai Rim region. Progress in Geography. 2020; 36(02): 171-181.
3. Sun XY, Wang J, Jin YT. Advances in research on pesticide pollution to the aquatic environment and health impact in China. Journal of Environment and Health. 2009;26(07): 649-652.
4. Brainerd E, Menon N. Seasonal effects of water quality: The hidden costs of the green revolution to infant and child health in India. Journal of Development Economics. 2014; 107: 49-64. doi: 10.1016/j.jdeveco.2013.11.004.
5. Lai WY. Pesticide use and health outcomes: evidence from agricultural water pollution in China. Journal of Environmental Economics and Management. 2017; 86: 93-120. doi:10.2139/ssrn.2751458.
6. He GJ, Liu T, Zhou MG. Straw burning, PM 2.5, and death: Evidence from China. Journal of Development Economics. 2020;145: 102468. doi: 10.1016/j.jdeveco.2020.102468.
7. Wong CM, Lai HK, Tsang H, Thach TQ, Thomas GN, et al. Satellite-based estimates of long-term exposure to fine particles and association with mortality in elderly Hong Kong residents. Environmental Health Perspectives. 2015;123(11):1167–1172. doi: 10.1289/ehp.1408264. Epub 2015 Apr 24. PMID: 25910279.
8. Sacks JD, Stanek LW, Luben TJ, Johns DO, Buckley BJ, et al. Particulate matter-induced health effects: who is susceptible? Environmental Health Perspectives. 2011;119(4):446-54. doi:10.1289/ehp.1002255. PMID: 20961824.
9. Cohen, G., Gerber, Y. Air pollution and successful aging: Recent evidence and new perspectives. Current Environmental Health Reports. 2017; 4:1-11. https://doi.org/10.1007/s40572-017-0127-2. PMID: 28101729.
10. Assembly of First Nations (AFN). Environmental health older adults and seniors (elders). Ottawa, Canada. 2009.
11. Tuttle L, Meng Q, Moya J, Johns DO. Consideration of age-related changes in behavior trends in older adults in assessing risks of environmental exposures. Journal of Aging and Health. 2013; 25(2):243-273. doi:10.1177/0898264312468032. PMID: 23223208
12. Health Canada. Health of older adults and the environment discussion paper. Ottawa, Canada. 2008.
13. Tong YF. Changes and challenges of labor supply in China in the context of population ageing. Population Research. 2014; 38(02): 52-60.
14. Li JX, Li CH. Health difference of the elderly between the rural and urban districts. Population Journal. 2014; 36(05):37-47.
15. Weeks WB, Kazis LE, Shen TJ, Cong ZX, Ren XH, et al. Differences in health-related quality of life in rural and urban veterans. American Journal of Public Health. 2004; 10:1762-1767. https://doi.org/10.2105/AJPH.94.10.1762.

16. Khanal SJ, Pokhrel RP, Pokharel B, Becker S, Giri B, et al. An episode of transboundary air pollution in the central Himalayas during agricultural residue burning season in North India. Atmospheric Pollution Research. 2022; 13(1): 101270. https://doi.org/10.1016/j.apr.2021.101270.
17. Huang L, Zhu YH, Wang Q, Zhu AS, Liu ZY, et al. Assessment of the effects of straw burning bans in China: Emissions, air quality, and health impacts. Science of The Total Environment. 2021; 789: 147935. https://doi.org/10.1016/j.scitotenv.2021.147935.
18. Nganchamung T, Robson M. Chemical fertilizer use and acute health effects among Chili farmers in Ubon Ratchathani Province, Thailand. Journal of Health Research.2017; 31(6): 427-435. doi:10.14456/jhr.2017.53
19. Gorman NM, Stjernberg E, Koehoorn M, Demers PA, Winters M, et al. Fertilizer use and self-reported respiratory and dermal symptoms among tree planters. Journal of Occupational and Environmental Hygiene. 2013; 10(1): 36-45. doi: 10.1080/ 15459624.2012.740994.
20. Qiao F, Huang J, Zhang L, Rozelle S. Pesticide use and farmers' health in China's rice production. China Agricultural Economic Review. 2012; 4: 468-484. https://doi.org/10.1108/17561371211284821.
21. Zhang WD, Sohngen B. Do U.S. anglers care about harmful algal blooms? A discrete choice experiment of Lake Erie recreational anglers. American Journal of Agricultural Economics, Agricultural and Applied Economics Association.2018; 100(3): 868-888. https://doi.org/10.1111/ajae.12130
22. Paudel J, Crago CL. Environmental externalities from agriculture: Evidence from water quality in the United States. American Journal of Agricultural Economics. 2021; 103: 185-210. https://doi.org/10.1111/ajae.12130
23. China pollution source census. Technical report on China pollution source census. Beijing: China Environmental Press, 2011.
24. National Bureau of Statistics. China statistical yearbook (2019). Beijing: China Statistics Pres, 2020.
25. Zahra, Bahadoran, Asghar. Nitrate-nitrite-nitrosamines exposure and the risk of type 1 diabetes: A review of current data. World Journal of Diabetes. 2016; 7:433. doi: 10.4239/wjd.v7.i18.433. PMID: 27795817
26. Fan AM, Steinberg VE. Health implications of nitrate and nitrite in drinking water: An update on methemoglobinemia occurrence and reproductive and developmental toxicity. Regulatory Toxicology and Pharmacology.1996; 23:35-43. doi:10.1006/rtph.1996.0006.
27. Kristensen P, Andersen A, Irgens LM. Testicular cancer and parental use of fertilizers in agriculture. Cancer epidemiology, biomarkers & prevention. 1996; 5:3-9. PMID: 8770459
28. Ward MH. Too much of a good thing? Nitrate from nitrogen fertilizers and cancer. Reviews on Environmental Health. 2009; 24, 357-363. doi:10.1515/REVEH.2009.24.4.357.
29. Forman D. Are nitrates a significant risk factor in human cancer? Cancer Surveys.1989; 8(2): 443-458. PMID: 2696589
30. Bivolarska A, Gatseva P. Thyroid status in pregnant women and association with nitrates as an environmental factor stimulating the manifestation of iodine deficiency. Trace Elements and Electrolyte. 2015; 32:60-64. doi: 10.5414/tex01365.
31. Manasaram DM, Backe LC, Moll DM. A review of nitrates in drinking water: Maternal exposure and adverse reproductive and developmental outcomes. Environmental Health Perspectives. 2006; 114: 320-327. doi:10.2307/3436672.
32. Uddin M.S, Kurosawa K. Effect of chemical nitrogen fertilizer application on the release of arsenic from sediment to groundwater in Bangladesh. Procedia Environmental Sciences. 2011; 4: 294-302. doi: 10.1016/j.proenv.2011.03.034.
33. Smith AH, Hopenhayn-Rich C, Bates MN. Cancer risks from arsenic in drinking water. Environmental Health Perspectives. 1992; 97:259. doi: 10.1289/ehp.9297259.
34. Johnson RJ, Wolinsky FD. The structure of health status among older adults: disease, disability, functional limitation, and perceived health. Journal of Health Social Behavior. 1993; 34: 105-121. doi:10.2307/2137238
35. Lei J, Su SP, Yu WM. Temporal and spatial pattern evolution and grouping prediction of non-point source pollution of chemical fertilizers in China. Chinese Journal of Eco-Agriculture. 2020; 28(07): 1079-1092. doi: 10.13930/j.cnki.cjea.190923.

36. Shen KR, Jin G, Fang X. Dose environmental regulation cause pollution to transfer nearby? Economic Research Journal. 2017; 52(05): 44-59. http://www.cesgw.cn/cn/mlInfo.aspx?m=20170217100514860651&n=20170609120846613735&tip=4.
37. Zhu FP, Zhang ZY, Jiang GL. Empirical study of the relationship between FDI and environmental regulation: An intergovernmental competition perspective. Economic Research Journal. 2011; 46(06): 133-145. http://www.cesgw.cn/cn/mlInfo.aspx?m=20110125121729123417&n=20110617154203400510&tip=10
38. Chen HP, Wu LP, Wang YB. Impacts of subsidy policy on grain production: An empirical analysis based on sub-provincial data of grain production from 2004—2007. Journal of Agro technical Economics. 2010; 4: 100-106.
39. Chen F, Fan QQ, Gao TM. Agricultural policies, food production and food production-adjustment ability. Economic Research Journal. 2010; 45(11): 101-114+140.
40. Ge JH, Zhou SD. An analysis of the economic determinants to agricultural non-point source pollution: Based on data of Jiangsu province during 1978-2009. Chinese Rural Economy, 2011; (5): 72-81.
41. Callaway B, Goodman-Bacon A, and Pedro H.C. Sant'Anna. Difference-in-differences with a continuous treatment. Working Paper, 2021.
42. Brettschneider C, Hajek A, Rohr S, Fuchs A, Weeg D, et al. Determinants of health-care costs in the oldest-old in Germany. The Journal of the Economics of Ageing. 2019; 14: 100200. https://doi.org/10.1016/j.jeoa.2019.100200.
43. Winograd CH. Targeting Strategies: An overview of criteria and outcomes. Journal of the American Geriatrics Society. 1991; 39:25-35. https://doi.org/10.1111/j.1532-5415.1991.tb05930.x
44. Speechley M, Tinetti M. Falls and injuries in frail and vigorous community elderly persons. Journal of the American Geriatrics Society. 1991; 39:46-52. doi: 10.1111/j.1532-5415. 1991.tb05905. x. PMID: 1987256.
45. Rockwood K, Stadnyk K, MacKnight C, McDowell I. A brief clinical instrument to classify frailty in elderly people. The Lancet. 1999; 353:205-206. doi: 10.1016/S0140-6736(98)04402-X. PMID: 9923878.
46. Lau LK, Wee SL, Mallya JU, Yap, Pang, et al. Physiological and cognitive determinants of gait in middle-aged, older-aged and oldest-aged Asian adults – The Yishun study. Aging and Health Research. 2021; 1(3): 100030. https://doi.org/10.1016/j.ahr.2021.100030.
47. Monti A, Doulazmi M, Nguyen-Michel VH, Pautas E, Mariani J, et al. Clinical characteristics of sleep apnea in middle-old and oldest-old inpatients: symptoms and comorbidities. Sleep Medicine. 2021; 82: 179-185. https://doi.org/10.1016/j.sleep.2021.04.017.
48. Sheahan M, Barrett CB, Goldvale, C. The unintended consequences of agricultural input intensifycation: human health implications of agro-chemical use in Sub-Saharan Africa. Working Paper Series. 2016; 234. https://www.afdb.org/en/documents/document/working-paper-234-the-unintended-consequences-of-agricultural-input-intensification-human-health-implications-of-agro-chemical-use-in-sub-saharan-africa-87634.
49. Cai XQ, Lu Y, Wu MQ. Does environmental regulation drive away inbound foreign direct investment? evidence from a quasi-natural experiment in China. Journal of Development Economics. 2016; 123: 73-85. doi: 10.1016/j.jdeveco.2016.08.003.
50. Ferrara LE, Chong A, Duryea S. Soap operas and fertility: evidence from Brazil. Applied Economics. 2012; 4: 1-31. doi:10.2139/ssrn.1820921.
51. Dai BZ. The old age health security in rural China: where to go? International Journal for Equity Health. 2015; 14: 119. doi: 10.1186/s12939-015-0224-5.
52. Mitra S, Gao Q, Chen W, Zhang YL. Health, work, and income among middle-aged and older adults: a panel analysis for China. The Journal of the Economics of Ageing. 2020; 17:100255. doi: 10.1016/j.jeoa.2020.100255.
53. Behncke, S. Does retirement trigger ill health? Health Economics. 2012;21: 282-300. doi:10.1002/hec.1712.

Supporting information

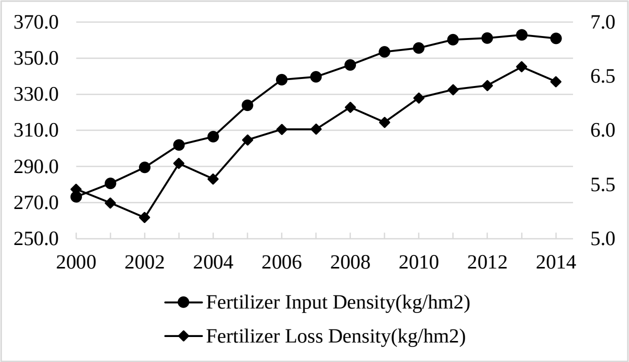

S1 Fig. Fertilizer input and fertilizer loss in China.

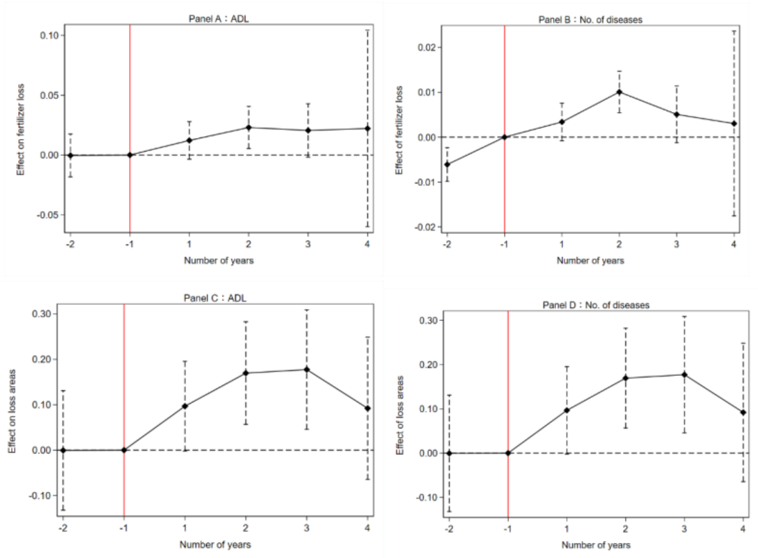

S2~~1~~ Fig. Parallel t~~T~~rend t~~T~~est.

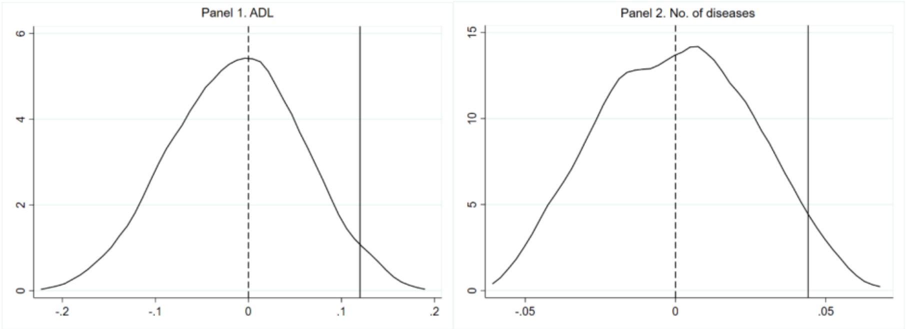

S32 Fig. Placebo Testtest.

Notes:
Panel 1 is the result of the ADL index placebo test; Panel 2 is the result of the placebo test of the number of patients.
The X-axis represents the estimated coefficients from 1000 random assignments.
The curve is the estimated kernel density distribution. The vertical type is a true estimate in columns (2) and (4) of Table 3.

Formatted: Indent: First line: 0", Line spacing: single

S1 Table. Basica regression results without control variables.

	ADL		No. of diseases	
	(1)	(2)	(3)	(4)
Fertilizerloss*Time	0.0175***		0.0052***	
	(0.0057)		(0.0016)	
Higharea*Time		0.1608***		0.0441***
		(0.0432)		(0.0124)
Control	NO	NO	NO	NO
Year FE	YES	YES	YES	YES
ID FE	YES	YES	YES	YES
Observations	32,302	32,302	32,467	32,467
R-squared	0.5474	0.5475	0.5597	0.5597

Notes:
*, **, *** are significant at the level of 10%, 5% and 1% respectively.
*, **, *** are significant at the level of 10%, 5% and 1% respectively.

S2 Table. Estimates of PSM-DID.

	Nearest neighbor		Radius		Kernel	
	ADL	No. of diseases	ADL	No. of diseases	ADL	No. of diseases
Fertilizerloss*Time	0.0695**	0.0143**	0.0267***	0.0065***	0.0267***	0.0065***
	(0.0273)	(0.0064)	(0.0085)	(0.0023)	(0.0085)	(0.0023)
Control	YES	YES	YES	YES	YES	YES
Year FE	YES	YES	YES	YES	YES	YES
ID FE	YES	YES	YES	YES	YES	YES
Observations	4532	4532	17801	17801	17801	17801
R-squared	0.5927	0.7152	0.5828	0.7049	0.5828	0.7049

Notes:
*, **, *** are significant at the level of 10%, 5% and 1% respectively.

S2 S3 Table. Heterogeneous estimation of yonger olds and oldest olds.

	(1)	(2)	(3)	(4)
	ADL		No. of diseases	
	Younger olds	Oldest olds	Younger olds	Oldest olds
Fertilizerloss*Time	-0.0006	0.0356***	0.0033**	0. 0086***
	(0.0038)	(0.0074)	(0.0014)	(0.0114)
Control	YES	YES	YES	YES
Year FE	YES	YES	YES	YES
ID FE	YES	YES	YES	YES
Observations	20536	22367	20620	22431
R-squared	0.0761	0.1359	0.3418	0.2951
p	0.0008***		0.0204**	

Notes:
*, **, *** are significant at the level of 10%, 5% and 1% respectively.
a*, **, *** are significant at the level of 10%, 5% and 1% respectively.

S3 S4 Table. Heterogeneous estimation of yonger olds, middle olds and oldest olds.

	(1)	(2)	(3)	(4)	(5)	(6)
		ADL			No. of diseases	
	Younger olds	Middle olds	Oldest olds	Younger olds	Middle olds	Oldest olds
Fertilizerloss*Time	0.0027	0.0000	0.0334***	0. 0018	0.0039*	0. 0074***
	(0.0040)	(0.0061)	(0.0071)	(0. 0022)	(0. 0021)	(0.0114)
Control	YES	YES	YES	YES	YES	YES
Year FE	YES	YES	YES	YES	YES	YES
ID FE	YES	YES	YES	YES	YES	YES
Observations	8332	10719	23852	8360	10769	23922
R-squared	0.0548	0.0885	0.1287	0.3416	0.3398	0.2954
p	P^{12}=0.7163			P^{45}=0.4969		
	P^{23}=0.0039***			P^{56}=0.1936		
	P^{13}=0.0029***			P^{46}=0.0419**		

Notes:
*, **, *** are significant at the level of 10%, 5% and 1% respectively.
a*, **, *** are significant at the level of 10%, 5% and 1% respectively.

S4 S5 Table. Robustness checks of fertilizer input and fertilizer input areas.

	ADL		No. of diseases	
	(1)	(2)	(3)	(4)
Fertilizer input*Time	0.0010***		0.0001*	
	(0.0003)		(0.0001)	
Higharea*Time		0.2168***		0.0061
		(0.0547)		(0.0148)
Control	YES	YES	YES	YES
Year FE	YES	YES	YES	YES
ID FE	YES	YES	YES	YES
Observations	32443	32278	32443	32443
R-squared	0.6905	0.5646	0.6905	0.6903

Notes:

*, **, *** are significant at the level of 10%, 5% and 1% respectively.

a*, **, *** are significant at the level of 10%, 5% and 1% respectively.

S5 S6. Table Additional robustness checks of fertilizer loss in different areas.

	Major Agri-producing areas		Major Rice-producing areas		Water resource rich areas	
	ADL index	No. of diseases	ADL index	No. of diseases	ADL index	No. of diseases
Fertilizerloss*Time	0.0283***	0.00200	0.0203***	0.00500***	0.0274	0.0161***
	(0.0082)	(0.0021)	(0.0066)	(0.0017)	(0.0175)	(0.0043)
Control Variables	YES	YES	YES	YES	YES	YES
Year FE	YES	YES	YES	YES	YES	YES
Individual ID FE	YES	YES	YES	YES	YES	YES
Observations	16880	16959	21528	21603	20526	20604
R-squared	0.572	0.695	0.549	0.677	0.533	0.667

Notes:

*, **, *** are significant at the level of 10%, 5% and 1% respectively.

a*, **, *** are significant at the level of 10%, 5% and 1% respectively.

S6 S7 Table. Additional robustness checks of fertilizer loss areas in different areas in different areas.

	Major Agri-producing areas		Major Rice-producing areas		Water resource rich areas	
	ADL index	No. of diseases	ADL index	No. of diseases	ADL index	No. of diseases
Higharea*Time	0.255***	0.0389**	0.163***	0.0452***	0.144**	0.0339**
	(0.0677)	(0.0171)	(0.0567)	(0.0141)	(0.0613)	(0.0158)
Control Variables	YES	YES	YES	YES	YES	YES
Year FE	YES	YES	YES	YES	YES	YES
Individual ID FE	YES	YES	YES	YES	YES	YES
Observations	16880	16959	21528	21603	20526	20604
R-squared	0.571	0.695	0.549	0.677	0.532	0.667

Notes:

*, **, *** are significant at the level of 10%, 5% and 1% respectively.

a*, **, *** are significant at the level of 10%, 5% and 1% respectively.

1. The fertilizer loss is continuous variable, and we dichotomized fertilizer loss by its pre-policy mean to compare the health effect in different regions. The average value of fertilizer loss is the national average of fertilizer loss; it is equal to 5.36kg/ha. High loss areas include Anhui, Shandong, Jiangsu, Guangdong, Hebei, Henan, Zhejiang, Hubei and Fujian, while low loss areas include Chongqing, Sichuan, Shaanxi, Guangxi, Hunan, Jiangxi, Heilongjiang, Jilin, Liaoning and Shanxi. In this paper, data from Beijing, Tianjin and Shanghai are deleted because of the small sample size and the small agricultural planting area.

2. Data source of loss coefficient: technical report of the first general survey of pollution sources.

3. The major agricultural-producing areas are Hebei Province, Inner Mongolia Autonomous Region, Heilongjiang Province, Jiangsu Province, Anhui Province, Shandong Province, Henan Province, Hubei Province, Hunan Province and Sichuan Province.

The major rice-producing areas are Heilongjiang Province, Jiangsu Province, Anhui Province, Jiangxi Province, Hubei Province, Hunan Province, Guangdong Province, Guangxi Zhuang Autonomous Region, Chongqing and Sichuan Province.

The water resource rich areas are Heilongjiang Province, Zhejiang Province, Anhui Province, Fujian Province, Jiangxi Province, Hubei Province, Hunan Province, Guangdong Province, Guangxi Zhuang Autonomous Region and Sichuan Province.

Response to Editor

Dear Dr. Qiu,

Thank you for coordinating, reviewing, and extending the invitation to revise our manuscript. We have treated yours as well as the reviewers' comments seriously and made significant modification to the manuscript. As follows, we will summarize revisions implemented following the two reviewers' comments before point by point responses.

For Reviewer1, we have:

1. Improved writing and readability by proving a better expression and revising Figure 1 and Figure 2.
2. Added the regression results of percentage changes and the details of TWFE model.

For Reviewer2, we have:

1. Revised the section of "parallel trend analysis" and explained the regression results in more details.
2. Added the regression results of propensity score matching (PSM) difference-in-differences (DID).
3. Offered the binary robustness check by changing the cutoff for the binary treatment effect.

We hope you find our revised manuscript acceptable for publication in PLOS ONE.

Sincerely,

Ying Wang

Cc. Hang Xiong and Chao Chen

Reviewer: 1

Thank you for the constructive comments on our manuscript. We have taken your comments seriously and substantially modified the manuscript. We believe our work has been significantly improved thanks to your comments. Please find below our response to each point you made with your original comments in italic.

The manuscript is much improved. I have the following comments before this paper can be published. I have also highlighted some minor comments such as grammar mistakes, typos, and formatting errors in the revised manuscript (see pdf if applicable).

1.Abstract: "ADL daily life index of 0.0147.." How much is this change in percentage?
Response： Thank you very much for your question. ADL daily index of 0.0147 is 0.2 percent change. We have added the percentages in the abstract. The empirical model results are in Table1. The explained variables are the logarithmic of health outcome variables.

Table 1 Empirical result of the logarithmic of health outcome variables.

	ADL		No. of diseases	
	(1)	(2)	(3)	(4)
Fertilizerloss*Time	0.0020***	0.0017**	0.0024	0.0047*
	(0.0006)	(0.0007)	(0.0030)	(0.0028)
Control	NO	YES	NO	YES
Year FE	YES	YES	YES	YES
ID FE	YES	YES	YES	YES
Observations	32189	32165	4426	4395
R-squared	0.5618	0.5719	0.5245	0.6093

Notes:
Robust standard errors for clustering at the individual level are reported in brackets.
*, **, *** are significant at the level of 10%, 5% and 1% respectively.

2.Figure 1: Can you add something about the pathways, such as through drinking water?
Response： Following your advice, we have revised the Figure 1.

3.I did not see a figure showing the Fertilizer loss in China (maybe you can put it into the Supporting information)
Response： Thank you very much for your suggestion. We have added S1 figure to demonstrate the Fertilizer Input and Fertilizer Loss in China in the Supporting information.

4.Figure 2: Could you add a vertical line to show treatment time?

Response： Following your advice, we have added a vertical line to show treatment line and we have replaced the figure2.

5.Table 3 Missing variable name for coefficients. Please add. Also, could you check the R2 for column (3)?

Response： Sorry about these errors. In our revised manuscript, we have carefully corrects these errors.

6.What are the model specifications for TWFE?

Response： Thanks for your question. We use two-way fixed effect model to estimate the health effect of fertilizer loss directly. We fix the individual and time effects both.

$$Health_{it} = \alpha_0 + \alpha_1 Fertilizerloss_{it} + \alpha_2 X_{it} + \gamma_t + \mu_i + \varepsilon_{it} \quad (1)$$

where $Health_{it}$ demotes the health outcome; α_1 identifies the marginal effect of fertilizer loss; X_{it} is a vector of control variables including individual, household, and provincial characteristics such as age, gender, health behavior, dietary habits, industrial pollution and hospitals, etc.; μ_i is a fixed effect unique to individuals i; γ_t is a time effect common to all individuals in year t; and ε_{it} is an error term.

7.Table 4 Do individual fixed effects mean the individual person (survey participants) fixed effects or province fixed effects?

Response： In this paper, individual fixed effects mean the individual person (survey participants) fixed effects. And we have revised by "ID FE" to make the expression clearer.

8.Section 3.2.1 "The differences between younger and middle olds are not significant in both ADL and No. of diseases". Do you expect that the effects are homogenous across ages? Are there any possible explanations if they are the same?

Response： Thank you for the questions. The health effects of pollution expose are complex and does not follow a simple linear or near-linear relationship. The elderly often has a higher susceptibility and a higher mortality rate (Wong et al., 2015; Cohen and Gerber, 2017). The heterogeneous effects are because of the age-related behaviors and the physical functions (Tuttle et al., 2013; Rockwood et al., 1999). Increasing variability in diseases and disabilities could be found when people aged and the aging people were more likely to fall and be hospitalized (Speechley and Tinetti, 1991; Winograd, 1991). The younger elderly had better physical functions, so effects on ADL

and No. of diseases are not significant. However, the younger elderly also has health risks. The elderly becomes suffered from the diseases related to fertilizer loss as the age growing. So a significant effect on No. of diseases could be observed among the middle olds elderly.

9.Table 5 what are the p values?

Response： P-values estimate the likelihood of obtaining the observed differences in coefficient estimates. The original hypothesis is there is no difference between groups (H_0: $\alpha_{group1} = \alpha_{group2}$).The model results reject the original hypothesis. It means that there is a significant difference between groups.

10.Section 3.3.1 Parallel trend test Why are they divided into five periods?

Response： Thank you very much for your question. There are six waves (2000, 2002, 2005, 2008, 2011, 2014) survey data. Two periods before the agricultural support policies, and four periods after treatment time. We choose the first period before the treatment time point as the benchmark group, so there are five periods. We have restructured this part.

11.Table S3 What do that superscripts for p values mean?

Response： P-values estimate the likelihood of obtaining the observed differences in coefficient estimates.

Response for comments in the PDF documents:

1. Thank you very much for the suggestion about typographical and grammatical errors. We have corrected the errors. And we have checked publications in the journal and reconfirmed that the supporting information citations as “S1 Fig” and “S1 Table”.

2. We have revised the section of conclusions and discussion and shortened the expression of Line344-347.

3. For response 9: We have added the details about the average values of fertilizer loss in note of Table 2 in our revised manuscript.

4. For response 17: There is a double-direction causality relationship between medical supply and health. And the medical supply is only a control variable, so we did not discuss the double-direction causality relationship. Further, we estimated health effect of fertilizer loss by deleting the medial supply variable (the results in Table 2). Also the negative effects between medical supply and health were found in other literatures (Lai, 2017; Ju,2022), when they controlled medical supply.

Table 2 Estimates without variable of medical supply

	ADL	No. of diseases
Fertilizerloss*Time	0.0173***	0.0051***
	(0.0061)	(0.0014)
Control	YES	YES
Year FE	YES	YES
ID FE	YES	YES
Observations	32165	32329
R-squared	0.5564	0.6903

Notes:
*, **, *** are significant at the level of 10%, 5% and 1% respectively.

Reviewer: 2

6. Your parallel trend analysis is appreciated. However, you might want to add an explanation on which figures are for low fertilizer regions and which are for the high fertilizer regions. Otherwise, I cannot understand. Also, the explanation "We divide the samples to five sections" is also confusing. You could consider rewriting this part.

Response： Thank you very much for your suggestion. In order to test whether the time staggered entry events of agricultural policy are effective exogenous shocks, we conduct a parallel test base on Equation (9):

$$H_{it} = \alpha + \sum_{k\geq -2, k\neq -1}^{k=4} \delta_k D_{it,k} \times Fertilizerloss_{it,k} + \lambda X_{it} + \gamma_t + \mu_i + \varepsilon_{it} \quad (9)$$

where $D_{it,k}$ is a binary variable that takes value of 1 when agricultural support policies are implemented. We use $k=-1$ to denote the base year. Panel A and Panel B of S2 Fig were the estimate results of fertilizer loss and Panel C and Panel showed the estimated coefficients when adopting a binary variable that takes values 1 as high loss areas. Following your advice, we have restructured the Section of Parallel trend test. We added the Equation (9) to explain how we do the parallel trend, and we explained S2 Fig in more detail.

7. I am concerned about this treatment. Although in some cases, adding linear coefficients could work, health factors are sometimes not linear. Do the authors have considered matching? If so, how does it go?

Response： Following your advice, three matching techniques are utilized to assess the health effect of fertilizer loss, namely nearest neighbor matching, the radius matching and the kernel matching. The matching results are reported in Table 3. The estimated coefficients are significant and it means that the basic results are robust. We have added the Table 3 in support information as S2 Table in our revised manuscript.

Table 3 Estimates of Matching

	Nearest neighbor		Radius		Kernel	
	ADL	No. of diseases	ADL	No. of diseases	ADL	No. of diseases
Fertilizerloss*Time	0.0695**	0.0143**	0.0267***	0.0065***	0.0267***	0.0065***
	(0.0273)	(0.0064)	(0.0085)	(0.0023)	(0.0085)	(0.0023)
Control	YES	YES	YES	YES	YES	YES
Year FE	YES	YES	YES	YES	YES	YES
ID FE	YES	YES	YES	YES	YES	YES
Observations	4532	4532	17801	17801	17801	17801
R-squared	0.5927	0.7152	0.5828	0.7049	0.5828	0.7049

[a] *, **, *** are significant at the level of 10%, 5% and 1% respectively.

8. I know it makes our life difficult if no package is available. However, assuming the level of fertilizer loss does not have different impacts is too strong. After all, this is the main argument of this study. I would suggest, that given the authors already adopt binary treatment in the robustness check, the authors should repeat the binary robustness check by calibrating the cutoff for the binary treatment effect.

Response： Following your advice, we have offered the binary robustness check by changing the cutoff for the binary treatment effect. We defined the high loss areas with the top 10%, 20%, 30% and 40% of the highest fertilizer loss intensity respectively. Table 4 reports the estimate coefficients in Equation (5), the basic regression results of binary treatment are robust to a variety of robustness checks.

Table 4 The Robustness Checks of Binary Treatment

	ADL				No. of diseases			
	10%	20%	30%	40%	10%	20%	30%	40%
Higharea*Time	0.1193**	0.1278**	0.1946***	0.1890***	0.0239*	0.0621***	0.0500***	0.0671***
	(0.0596)	(0.0525)	(0.0500)	(0.0491)	(0.0142)	(0.0122)	(0.0119)	(0.0120)
Control	YES	YES	YES	YES	YES	YES	YES	YES
Year FE	YES	YES	YES	YES	YES	YES	YES	YES
ID FE	YES	YES	YES	YES	YES	YES	YES	YES
Observations	32,189	32,189	32,189	32,189	32,353	32,353	32,353	32,353
R-squared	0.5620	0.5621	0.5623	0.5623	0.6898	0.6901	0.6900	0.6902

[a] robust standard error for clustering at the individual level are reported in brackets; *, **, *** are significant at the level of 10%, 5% and 1% respectively

Reference

1. Wong CM, Lai HK, Tsang H, et al. Satellite-based estimates of long-term exposure to fine particles and association with mortality in elderly Hong Kong residents. Environmental Health Perspectives. 2015;123(11):1167-1172
2. Cohen, G., Gerber, Y. Air pollution and successful aging: Recent evidence and new perspectives. Current Environmental Health Reports. 2017; 4:1-11. https://doi.org/10.1007/s40572-017-0127-2. PMID: 28101729.
3. Tuttle L, Meng Q, Moya J, Johns DO. Consideration of age-related changes in behavior trends in older adults in assessing risks of environmental exposures. Journal of Aging and Health. 2013; 25(2):243-273.
4. Rockwood K., Stadnyk K., MacKnight C., et al. A Brief Clinical Instrument to Classify Frailty in Elderly People. The Lancet. 1999; 353: 205-206.
5. Speechley M. and Tinetti M. Falls and Injuries in Frail and Vigorous Community Elderly Persons. Journal of the American Geriatrics Society.1991; 39: 46-52.
6. Winograd C.H. Targeting Strategies：An Overview of Criteria and Outcomes. Journal of the American Geriatrics Society. 1991; 39: 25S-35S.
7. Lai WY. Pesticide use and health outcomes: evidence from agricultural water pollution in China. Journal of Environmental Economics and Management. 2017; 86: 93-120.
8. Ju K, Lu LY, Chen T, et al. Does long-term exposure to air pollution impair physical and mental health in the middle-aged and older adults? — A causal empirical analysis based on a longitudinal nationwide cohort in China. Science of The Total Environment. 2022; 87: 154312.
