# Supplementary material for: Agricultural non-point source pollution and health of the elderly in rural China
Source: PLoS One. 2022 Oct 14;17(10):e0274027. doi: 10.1371/journal.pone.0274027 (PMC9565375; doi:10.1371/journal.pone.0274027)
Supplement: Attachment — Submitted filename: Review.docx [file pone.0274027.s005.docx]

Review

This is an important study that covers an important question. Previous studies have shown that fertilizer pollution damage residents’ health and this study intends to show whether the elder residents are more vulnerable to fertilizer pollution. However, there are many major problems with the current manuscript.

1. Although it is important to examine the regional inequality in the medical outcome, the most important question is: why aged people? Previous studies show that fertilizer loss damages residents, then the natural steps should be who are more subjected to harm from fertilizer loss. Therefore, citing a study to show the difference between elder residents and younger residents is appreciated.
2. Therefore, hypothesis 1 needs transition. If fertilizer loss is harmful to health, in general, is known, why do we have to explore its effect on aged residents, before showing whether there is a difference between elder and younger residents? Are they more vulnerable or more resilient than the younger group? This problem can be solved by citing studies showing fertilizer overuse has a different effect on elder and younger people.
3. For hypothesis 2, you might want to add more explanation on how the outcome variables are correlated with the “rejuvenation of disease”. How do you define the term?
4. Hypothesis 3 makes two points, and should be separated; their contents are also made sense by common wisdom, but the literature in the manuscript seems not to correlate with them.
5. The linear regression coefficients are correlations, not “impact”. The impact has a causal implication.
6. This research uses DID, but the research did not check the parallel trends before 2004. In this study, the main analysis uses data after 2004 and separates the provinces into high-loss and low-loss. However, as far as I am aware, there is no mention of how the health outcomes trend before 2004. For DID, parallel trends assumption is essential. I randomly search for a blog from the world bank that could help the authors to have the idea (<https://blogs.worldbank.org/impactevaluations/revisiting-difference-differences-parallel-trends-assumption-part-i-pre-trend>). Your placebo test does not correlate with this problem.
7. Even if the parallel trend assumption holds, another problem is how to support the assumption that all the residents are treated to high fertilizer overuse randomly. High fertilizer loss could also imply a lack of management or facility, which also leads to high medical loss. For example, it can be argued that the aged person exposed to extra use of fertilizer tend to live in provinces with lower medical resources (agriculture province such as Hubei are also subjected to fertilizer overuse). In short, the author should consider blocking potential causal paths.
8. Another problem that comes with DID is the treatment effect. In formula (4), it seems the treatment effect is continuous. However, the traditional DID does not allow for the continuous treatment effect and could miscalculate S.D and perform the wrong t-test. I suggest reading this paper and reconsidering this part:

Callaway, B., Goodman-Bacon, A., & Sant’Anna, P. H. C. (2021). Difference-in-Differences with a Continuous Treatment. ArXiv:2107.02637 [Econ]. <http://arxiv.org/abs/2107.02637>

Minor problems are listed as follows:

1. Replacing the word “rejuvenation of disease”. Many definitions can be found on the internet, so it might be helpful to address this word.
2. The research can be more specific on the unit of analysis. “Matches micro-level individual health data and provincial-level chemical fertilizer” is confusing.
3. No need to discuss policy implications in basic regression results of health. I was referring to the line(L)229-L235, L261-257,
4. When you use “1% level is significant”, do you mean “significant at 99% CI”?
5. L316-L319: Are you referring to table 6 or table 7?
6. Results of the placebo test, the Effect of fertilizer input on health, and the Effect in different areas can be moved to the appendix.

To sum up, in its current form, this manuscript is not ready to publish. However, if the author is willing to address these mentioned problems, then the topic is interesting and contributes to the existing literature.
