# Supplementary material for: Agricultural non-point source pollution and health of the elderly in rural China
Source: PLoS One. 2022 Oct 14;17(10):e0274027. doi: 10.1371/journal.pone.0274027 (PMC9565375; doi:10.1371/journal.pone.0274027)
Supplement: Attachment — Submitted filename: Response to Reviewers.docx [file pone.0274027.s008.docx]

### Response to Editor

Dear Dr. Qiu,

Thank you for coordinating, reviewing, and extending the invitation to revise our manuscript. We have treated yours as well as the reviewers’ comments seriously and made significant modification to the manuscript. As follows, we will summarize revisions implemented following the two reviewers’ comments before point by point responses.

For Reviewer1, we have:

1. Improved writing and readability by proving a better expression and revising Figure 1 and Figure 2.
2. Added the regression results of percentage changes and the details of TWFE model.

For Reviewer2, we have:

1. Revised the section of “parallel trend analysis” and explained the regression results in more details.
2. Added the regression results of propensity score matching (PSM) difference-in-differences (DID).
3. Offered the binary robustness check by changing the cutoff for the binary treatment effect.

We hope you find our revised manuscript acceptable for publication in PLOS ONE.

Sincerely,

Ying Wang

Cc. Hang Xiong and Chao Chen

### Reviewer: 1

Thank you for the constructive comments on our manuscript. We have taken your comments seriously and substantially modified the manuscript. We believe our work has been significantly improved thanks to your comments. Please find below our response to each point you made with your original comments in italic.

*The manuscript is much improved. I have the following comments before this paper can be published. I have also highlighted some minor comments such as grammar mistakes, typos, and formatting errors in the revised manuscript (see pdf if applicable).*

*1.Abstract: “ADL daily life index of 0.0147..” How much is this change in percentage?*

***Response：***Thank you very much for your question. ADL daily index of 0.0147 is 0.2 percent change. We have added the percentages in the abstract. The empirical model results are in Table1. The explained variables are the logarithmic of health outcome variables.

**Table 1 Empirical result of the logarithmic of health outcome variables.**

|  | ADL | | No. of diseases | |
| --- | --- | --- | --- | --- |
|  | (1) | (2) | (3) | (4) |
| Fertilizerloss*Time | 0.0020*** | 0.0017** | 0.0024 | 0.0047* |
|  | (0.0006) | (0.0007) | (0.0030) | (0.0028) |
| Control | NO | YES | NO | YES |
| Year FE | YES | YES | YES | YES |
| ID FE | YES | YES | YES | YES |
| Observations | 32189 | 32165 | 4426 | 4395 |
| R-squared | 0.5618 | 0.5719 | 0.5245 | 0.6093 |

Notes:

Robust standard errors for clustering at the individual level are reported in brackets.

*, **, *** are significant at the level of 10%, 5% and 1% respectively.

*2.Figure 1: Can you add something about the pathways, such as through drinking water?*

***Response：***Following your advice, we have revised the Figure 1.

*3.I did not see a figure showing the Fertilizer loss in China (maybe you can put it into the Supporting information)*

***Response：***Thank you very much for your suggestion. We have added S1 figure to demonstrate the Fertilizer Input and Fertilizer Loss in China in the Supporting information.

*4.Figure 2: Could you add a vertical line to show treatment time?*

***Response：***Following your advice, we have added a vertical line to show treatment line and we have replaced the figure2.

*5.Table 3 Missing variable name for coefficients. Please add. Also, could you check the R2 for column (3)?*

***Response：***Sorry about these errors. In our revised manuscript, we have carefully corrects these errors.

*6.What are the model specifications for TWFE?*

***Response：***Thanks for your question. We use two-way fixed effect model to estimate the health effect of fertilizer loss directly. We fix the individual and time effects both.

$Health_{it}=\alpha_{0}+\alpha_{1}Fertilizerloss_{it}+\alpha_{2}X_{it}+\gamma_{t}+\mu_{i}+\varepsilon_{it}$ (1)

where$Health_{it}$ demotes the health outcome; $\alpha_{1}$identifies the marginal effect of fertilizer loss; $X_{it}$is a vector of control variables including individual, household, and provincial characteristics such as age, gender, health behavior, dietary habits, industrial pollution and hospitals, etc.;$\mu_{i}$is a fixed effect unique to individuals$i$; $\gamma_{t}$is a time effect common to all individuals in year $t$; and$\varepsilon_{it}$ is an error term.

*7.Table 4 Do individual fixed effects mean the individual person (survey participants) fixed effects or province fixed effects?*

***Response：***In this paper, individual fixed effects mean the individual person (survey participants) fixed effects. And we have revised by “ID FE” to make the expression clearer.

*8.Section 3.2.1 “The differences between younger and middle olds are not significant in both ADL and No. of diseases”. Do you expect that the effects are homogenous across ages? Are there any possible explanations if they are the same?*

***Response：***Thank you for the questions. The health effects of pollution expose are complex and does not follow a simple linear or near-linear relationship. The elderly often has a higher susceptibility and a higher mortality rate (Wong et al., 2015; Cohen and Gerber, 2017). The heterogeneous effects are because of the age-related behaviors and the physical functions (Tuttle et al., 2013; Rockwood et al., 1999). Increasing variability in diseases and disabilities could be found when people aged and the aging people were more likely to fall and be hospitalized (Speechley and Tinetti, 1991; Winograd, 1991). The younger elderly had better physical functions, so effects on ADL and No. of diseases are not significant. However, the younger elderly also has health risks. The elderly becomes suffered from the diseases related to fertilizer loss as the age growing. So a significant effect on No. of diseases could be observed among the middle olds elderly.

*9.Table 5 what are the p values?*

***Response：***P-values estimate the likelihood of obtaining the observed differences in coefficient estimates. The original hypothesis is there is no difference between groups ($\text{H}_{0}\text{：}\alpha_{group1}=\alpha_{group2}$).The model results reject the original hypothesis. It means that there is a significant difference between groups.

*10.Section 3.3.1 Parallel trend test Why are they divided into five periods?*

***Response：***Thank you very much for your question. There are six waves (2000, 2002, 2005, 2008, 2011, 2014) survey data. Two periods before the agricultural support policies, and four periods after treatment time. We choose the first period before the treatment time point as the benchmark group, so there are five periods. We have restructured this part.

*11.Table S3 What do that superscripts for p values mean?*

***Response：***P-values estimate the likelihood of obtaining the observed differences in coefficient estimates.

**Response for comments in the PDF documents:**

1. Thank you very much for the suggestion about typographical and grammatical errors. We have corrected the errors. And we have checked publications in the journal and reconfirmed that the supporting information citations as “S1 Fig” and “S1 Table”.
2. We have revised the section of conclusions and discussion and shortened the expression of Line344-347.
3. For response 9: We have added the details about the average values of fertilizer loss in note of Table 2 in our revised manuscript.
4. For response 17: There is a double-direction causality relationship between medical supply and health. And the medical supply is only a control variable, so we did not discuss the double-direction causality relationship. Further, we estimated health effect of fertilizer loss by deleting the medial supply variable (the results in Table 2). Also the negative effects between medical supply and health were found in other literatures (Lai, 2017; Ju,2022), when they controlled medical supply.

**Table 2 Estimates without variable of medical supply**

|  | ADL | No. of diseases |
| --- | --- | --- |
| Fertilizerloss*Time | 0.0173*** | 0.0051*** |
|  | (0.0061) | (0.0014) |
| Control | YES | YES |
| Year FE | YES | YES |
| ID FE | YES | YES |
| Observations | 32165 | 32329 |
| R-squared | 0.5564 | 0.6903 |

Notes:

*, **, *** are significant at the level of 10%, 5% and 1% respectively.

### Reviewer: 2

*6. Your parallel trend analysis is appreciated. However, you might want to add an explanation on which figures are for low fertilizer regions and which are for the high fertilizer regions. Otherwise, I cannot understand. Also, the explanation "We divide the samples to five sections" is also confusing. You could consider rewriting this part.*

***Response：***Thank you very much for your suggestion. In order to test whether the time staggered entry events of agricultural policy are effective exogenous shocks, we conduct a parallel test base on Equation (9):

$H_{it}=\alpha\text{+}\sum_{k\geq-2,k\neq-1}^{k=4} \delta_{k}D_{it,k}\times Fertilizerloss_{it,k}+\lambda X_{it}+\gamma_{t}+\mu_{i}+\varepsilon_{it}$ (9)

where$D_{it,k}$ is a binary variable that takes value of 1 when agricultural support policies are implemented. We use $k=-1$to denote the base year. Panel A and Panel B of S2 Fig were the estimate results of fertilizer loss and Panel C and Panel showed the estimated coefficients when adopting a binary variable that takes values 1 as high loss areas. Following your advice, we have restructured the Section of Parallel trend test. We added the Equation (9) to explain how we do the parallel trend, and we explained S2 Fig in more detail.

*7. I am concerned about this treatment. Although in some cases, adding linear coefficients could work, health factors are sometimes not linear. Do the authors have considered matching? If so, how does it go?*

***Response：***Following your advice, three matching techniques are utilized to assess the health effect of fertilizer loss, namely nearest neighbor matching, the radius matching and the kernel matching. The matching results are reported in Table 3. The estimated coefficients are significant and it means that the basic results are robust. We have added the Table 3 in support information as S2 Table in our revised manuscript.

**Table 3 Estimates of Matching**

|  | Nearest neighbor | | Radius | | Kernel | |
| --- | --- | --- | --- | --- | --- | --- |
|  | ADL | No. of diseases | ADL | No. of diseases | ADL | No. of diseases |
| Fertilizerloss*Time | 0.0695** | 0.0143** | 0.0267*** | 0.0065*** | 0.0267*** | 0.0065*** |
|  | (0.0273) | (0.0064) | (0.0085) | (0.0023) | (0.0085) | (0.0023) |
| Control | YES | YES | YES | YES | YES | YES |
| Year FE | YES | YES | YES | YES | YES | YES |
| ID FE | YES | YES | YES | YES | YES | YES |
| Observations | 4532 | 4532 | 17801 | 17801 | 17801 | 17801 |
| R-squared | 0.5927 | 0.7152 | 0.5828 | 0.7049 | 0.5828 | 0.7049 |

^a^ *, **, *** are significant at the level of 10%, 5% and 1% respectively.

*8. I know it makes our life difficult if no package is available. However, assuming the level of fertilizer loss does not have different impacts is too strong. After all, this is the main argument of this study. I would suggest, that given the authors already adopt binary treatment in the robustness check, the authors should repeat the binary robustness check by calibrating the cutoff for the binary treatment effect.*

***Response：***Following your advice, we have offered the binary robustness check by changing the cutoff for the binary treatment effect. We defined the high loss areas with the top 10%, 20%, 30% and 40% of the highest fertilizer loss intensity respectively. Table 4 reports the estimate coefficients in Equation (5), the basic regression results of binary treatment are robust to a variety of robustness checks.

**Table 4 The Robustness Checks of Binary Treatment**

|  | ADL | | | | No. of diseases | | | |
| --- | --- | --- | --- | --- | --- | --- | --- | --- |
|  | 10% | 20% | 30% | 40% | 10% | 20% | 30% | 40% |
| Higharea*Time | 0.1193** | 0.1278** | 0.1946*** | 0.1890*** | 0.0239* | 0.0621*** | 0.0500*** | 0.0671*** |
|  | (0.0596) | (0.0525) | (0.0500) | (0.0491) | (0.0142) | (0.0122) | (0.0119) | (0.0120) |
| Control | YES | YES | YES | YES | YES | YES | YES | YES |
| Year FE | YES | YES | YES | YES | YES | YES | YES | YES |
| ID FE | YES | YES | YES | YES | YES | YES | YES | YES |
| Observations | 32,189 | 32,189 | 32,189 | 32,189 | 32,353 | 32,353 | 32,353 | 32,353 |
| R-squared | 0.5620 | 0.5621 | 0.5623 | 0.5623 | 0.6898 | 0.6901 | 0.6900 | 0.6902 |

^a^ robust standard error for clustering at the individual level are reported in brackets; *, **, *** are significant at the level of 10%, 5% and 1% respectively

### Reference

1. Wong CM, Lai HK, Tsang H, et al. Satellite-based estimates of long-term exposure to fine particles and association with mortality in elderly Hong Kong residents. Environmental Health Perspectives. 2015;123(11):1167-1172
2. Cohen, G., Gerber, Y. Air pollution and successful aging: Recent evidence and new perspectives. Current Environmental Health Reports. 2017; 4:1-11. https://doi.org/10.1007/s40572-017-0127-2. PMID: 28101729.
3. Tuttle L, Meng Q, Moya J, Johns DO. Consideration of age-related changes in behavior trends in older adults in assessing risks of environmental exposures. Journal of Aging and Health. 2013; 25(2):243-273.
4. Rockwood K., Stadnyk K., MacKnight C., et al. A Brief Clinical Instrument to Classify Frailty in Elderly People. The Lancet. 1999; 353: 205-206.
5. Speechley M. and Tinetti M. Falls and Injuries in Frail and Vigorous Community Elderly Persons. Journal of the American Geriatrics Society.1991; 39: 46-52.
6. Winograd C.H. Targeting Strategies：An Overview of Criteria and Outcomes. Journal of the American Geriatrics Society. 1991; 39: 25S-35S.
7. Lai WY. Pesticide use and health outcomes: evidence from agricultural water pollution in China. Journal of Environmental Economics and Management. 2017; 86: 93-120.
8. Ju K, Lu LY, Chen T, et al. Does long-term exposure to air pollution impair physical and mental health in the middle-aged and older adults? — A causal empirical analysis based on a longitudinal nationwide cohort in China. Science of The Total Environment. 2022; 87: 154312.
