# Supplementary material for: Agricultural non-point source pollution and health of the elderly in rural China
Source: PLoS One. 2022 Oct 14;17(10):e0274027. doi: 10.1371/journal.pone.0274027 (PMC9565375; doi:10.1371/journal.pone.0274027)
Supplement: Attachment — Submitted filename: Response to Reviewers.docx [file pone.0274027.s010.docx]

### Response to Editor

Dear Dr. Qiu,

Thank you for coordinating, reviewing, and inviting revise our manuscript. We have treated yours as well as the reviewers’ comments seriously and made significant modification to the manuscript. As follows, we will summarize revisions implemented following the two reviewers’ comments before point by point responses.

For Reviewer1, we have:

1. Improved writing and readability by proving a better expression.
2. Revised the section of “conclusions and discussion” by explaining the data limitations.

We hope you find our revised manuscript acceptable for publication in PLOS ONE.

Sincerely,

Ying Wang

Cc. Hang Xiong and Chao Chen

### Reviewer: 1

Thank you for the constructive comments on our manuscript. We have taken your comments seriously and substantially modified the manuscript. We believe our work has been significantly improved thanks to your comments. Please find below our response to each point you made with your original comments in italic.

*The manuscript has been improved compared to last version. The “conclusion and discussion” section can be further improved. Please also go over the whole draft to make sure there are no grammar mistakes and typos. Please see more comments in the attached pdf.*

*1. The “conclusion and discussion” section can be further improved.*

***Response:*** Thank you very much for your suggestion. We have revised the section of “conclusion and discussion” by explaining the data limitations. We explored the effect of agricultural non-point source pollution on public health using fertilizer loss as a representative. We regarded animal husbandry pollution as point source pollution, but there was a small part of free range animal husbandry in rural China, which might cause non-point source pollution. Because of the limitation of data, we ignored this part of non-point source pollution. Also, loss of pesticides would also cause non-point source pollution, but there was no pesticide loss data, so we only controlled pesticides use in the empirical model. The non-point source pollution in animal husbandry and pesticides is also worth discussing if there is more wanted data.

***2.****Comments in the PDF documents:*

***Response:*** Thank you very much for the suggestion about typographical and grammatical errors. We have corrected the errors. We have checked the whole draft about the grammar mistakes and typos.
